# Copper-mediated oxidative C−H/N−H activations with alkynes by removable hydrazides

**DOI:** 10.3762/bjoc.17.113

**Published:** 2021-07-08

**Authors:** Feng Xiong, Bo Li, Chenrui Yang, Liang Zou, Wenbo Ma, Linghui Gu, Ruhuai Mei, Lutz Ackermann

**Affiliations:** 1 Key Laboratory of Coarse Cereal Processing, Ministry of Agriculture and Rural Affairs, College of Food and Biological Engineering, Chengdu University, Chengdu 610106, P.R. China; 2 Antibiotics Research and Re-evaluation Key Laboratory of Sichuan Province, Sichuan Industrial Institute of Antibiotics, School of Pharmacy, Chengdu University, Chengdu 610052, P.R. China; 3 Institut für Organische und Biomolekulare Chemie, Georg-August-Universität Göttingen, Tammannstraße 2, 37077 Göttingen, Germany and 4Wöhler Research Institute for Sustainable Chemistry (WISCh), Georg-August-Universität Göttingen, Tammannstraße 2, 37077 Göttingen, Germany

**Keywords:** benzhydrazides, copper, 3-methyleneisoindolin-1-one, removable directing group

## Abstract

The efficient copper-mediated oxidative C–H alkynylation of benzhydrazides was accomplished with terminal alkynes. Thus, a heteroaromatic removable *N*-2-pyridylhydrazide allowed for domino C–H/N–H functionalization. The approach featured remarkable functional group compatibility and ample substrate scope. Thereby, highly functionalized aromatic and heteroaromatic isoindolin-1-ones were accessed with high efficacy with rate-limiting C–H cleavage.

## Introduction

Inexpensive copper-promoted oxidative C−H activations [1–11] have been recognized as competent tools for the efficient assembly and late-stage functionalization of organic molecules due to the natural abundance and versatile reactivity. Early examples of copper-promoted C−H activation of 2-arylpyridines were disclosed by Yu et al. [12] and Chatami et al. [13] independently. Inspired by these studies, various copper-induced C−H functionalizations, such as arylations, alkynylations, cyanations, aminations, nitrations, oxygenations, thiolations, halogenations, and phosphorylations, among others, were accomplished [14–19].

**Figure 1:**
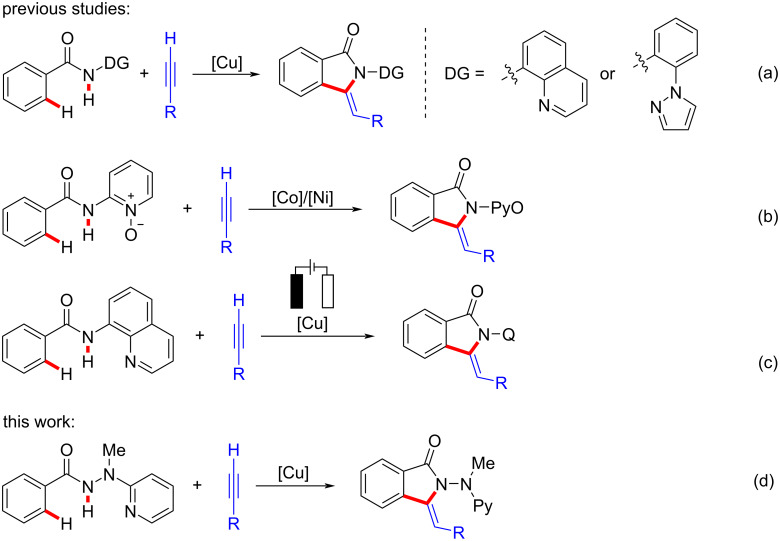
Assembly of 3-methyleneisoindolin-1-one via 3d transition metal-mediated/catalyzed oxidative C−H/N−H activation.

The 3-methyleneisoindolin-1-one moiety represents a key structure motif in natural products [20–23] or important pharmacophores [24]. In this context, You [25], Huang [26], Liu [27], Li [28], and co-workers elegantly disclosed copper-mediated/catalyzed cascade C−H alkynylation and annulation with terminal alkynes to afford 3-methyleneisoindolinone derivatives, through the assistance of 8-aminoquinoline [29] or 2-aminophenyl-1*H*-pyrazole [30] auxiliaries (Figure 1a). Besides, the cobalt(II)- [31] or nickel(II)-catalyzed [32–33], pyridine oxide (PyO)-directed tandem alkynylation/annulation was realized by Niu and Song et al., which also provided the 3-methyleneisoindolin-1-one scaffolds (Figure 1b). Notably, a sustainable cupraelectro-catalyzed alkyne annulation was very recently achieved by Ackermann et al., which gave rapid access to synthetically meaningful isoindolones (Figure 1c) [34]. In spite of these indisputable advances, the successful removal of the directing groups to deliver the free-NH 3-methyleneisoindolin-1-one has thus far unfortunately proven elusive [35].

2-(1-Methylhydrazinyl)pyridine (MHP) [36] was identified as a powerful removable bidentate directing group, which found widespread application in various cobalt-catalyzed C−H activations [37–40]. Thus, our group also accomplished a set of electrochemical cobalt-catalyzed C−H activations with the MHP auxiliary [41–44]. In continuation of studies on sustainable 3d transition metal-catalyzed C−H activation [41–49], we have now discovered a robust copper-promoted oxidative C−H/N−H functionalization with terminal alkynes (Figure 1d). Notable advantages of our protocol include: 1) removable MHP auxiliary used for copper-mediated oxidative C–H activations, 2) excellent functional group tolerance and compatibility with valuable heterocycles, and 3) mechanistic studies toward copper-mediated oxidative C−H alkynylations.

## Results and Discussion

We initiated our investigation by utilizing benzhydrazide **1a** and ethynylbenzene (**2a**) as the standard substrates (Table 1). After preliminary solvent optimization, we discovered that the desired *ortho*-selective C−H activation occurred efficiently by the treatment of hydrazide **1a** with terminal alkyne **2a** and a stoichiometric amount of Cu(OAc)_2_ in DMSO (Table 1, entries 1–3). Reaction optimization revealed that the most appropriate temperature was 90 °C (Table 1, entries 3–6). An evaluation of bases showed that Na_2_CO_3_ was optimal (Table 1, entries 7–11). The best result was obtained when Cu(OAc)_2_ (1.3 equiv) was utilized in DMSO (6.0 mL, Table 1, entries 12–14). A similar result was obtained when Cu(OAc)_2_⋅H_2_O was used instead of Cu(OAc)_2_ (Table 1, entry 15). Only a trace amount of product **3aa** was observed in the absence of either Cu(OAc)_2_ or Na_2_CO_3_ (Table 1, entries 16 and 17). When the reaction was performed under a nitrogen atmosphere, the efficacy was significantly decreased (Table 1, entry 18).

**Table 1:** Optimization of the copper-mediated C−H/N−H functionalization with terminal alkyne **2a**.^a^

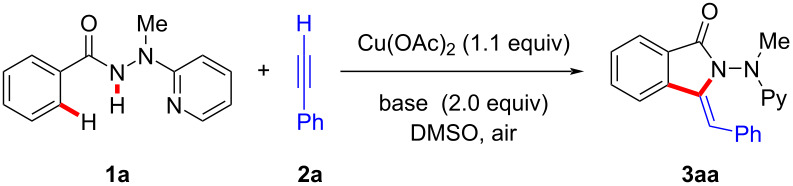					

entry	solvent	base	*T* (°C)	*Z*/*E*	yield (%)

1	DMF	Na_2_CO_3_	90	—	trace
2	NMP	Na_2_CO_3_	90	—	trace
3	DMSO	Na_2_CO_3_	90	12:1	67
4	DMSO	Na_2_CO_3_	110	8:1	57
5	DMSO	Na_2_CO_3_	80	15:1	41
6	DMSO	Na_2_CO_3_	60	—	27
7	DMSO	NaOAc	90	—	25
8	DMSO	NaOPiv	90	—	30
9	DMSO	K_2_CO_3_	90	18:1	58
10	DMSO	Cs_2_CO_3_	90	20:1	44
11	DMSO	DBU	90	—	13
12^b^	DMSO	Na_2_CO_3_	90	12:1	42
13^c^	DMSO	Na_2_CO_3_	90	9:1	83
14^c,d^	DMSO	Na_2_CO_3_	90	13:1	89
15^d,e^	DMSO	Na_2_CO_3_	90	12:1	86
16	DMSO	—	90	—	trace
17^f^	DMSO	Na_2_CO_3_	90	—	trace
18^g^	DMSO	Na_2_CO_3_	90	—	37

^a^Reaction conditions: **1a** (0.30 mmol), **2a** (0.90 mmol), Cu(OAc)_2_ (1.1 equiv), base (2.0 equiv), solvent (3.0 mL), 15 h, under air. ^b^Cu(OAc)_2_ (0.8 equiv). ^c^Cu(OAc)_2_ (1.3 equiv). ^d^DMSO (6.0 mL). ^e^Cu(OAc)_2_⋅H_2_O (1.3 equiv). ^f^Without Cu(OAc)_2_. ^g^Under N_2_.

**Scheme 1:**
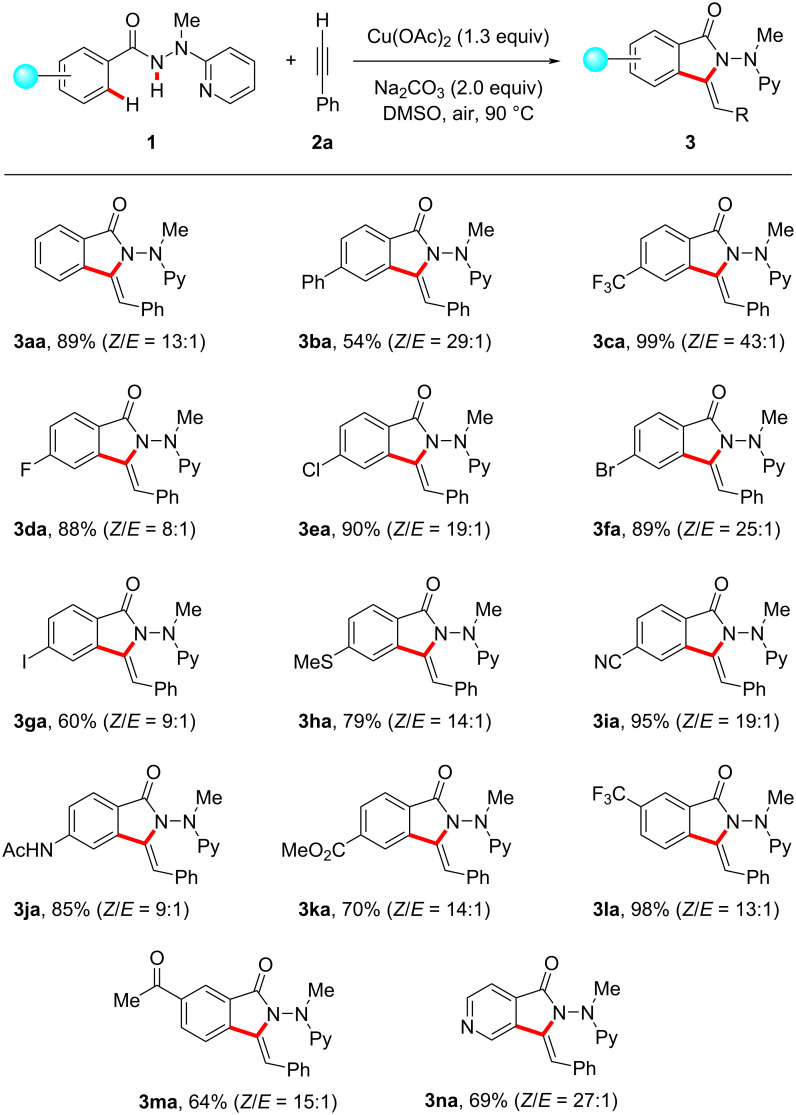
Copper-mediated oxidative C−H/N−H functionalization of hydrazides **1** with ethynylbenzene (**2a**).

We next examined the versatility of the copper-promoted ethynylbenzene (**2a**) annulation with various benzhydrazides **1** under the optimized reaction conditions (Scheme 1). To our delight, hydrazides **1** with electron-donating or electron-withdrawing substituents were efficiently converted in the C–H/N–H activation annulation process. Notably, a wide range of valuable electrophilic functional groups, such as halogen, methylthio, cyano, amino, and ester groups, were well compatible, which should prove instrumental for the further diversification of the thus obtained 3-methyleneisoindolin-1-ones **3da**–**ka**. For substrates bearing two potential reactive sites, the annulation selectively took place at the less congested *ortho*-C−H bond (see **3la** and **3ma**). Moreover, the challenging isonicotinic acid hydrazide **1n** was also amenable to this protocol and delivered the desired product **3na** with high regioselectivity.

**Scheme 2:**
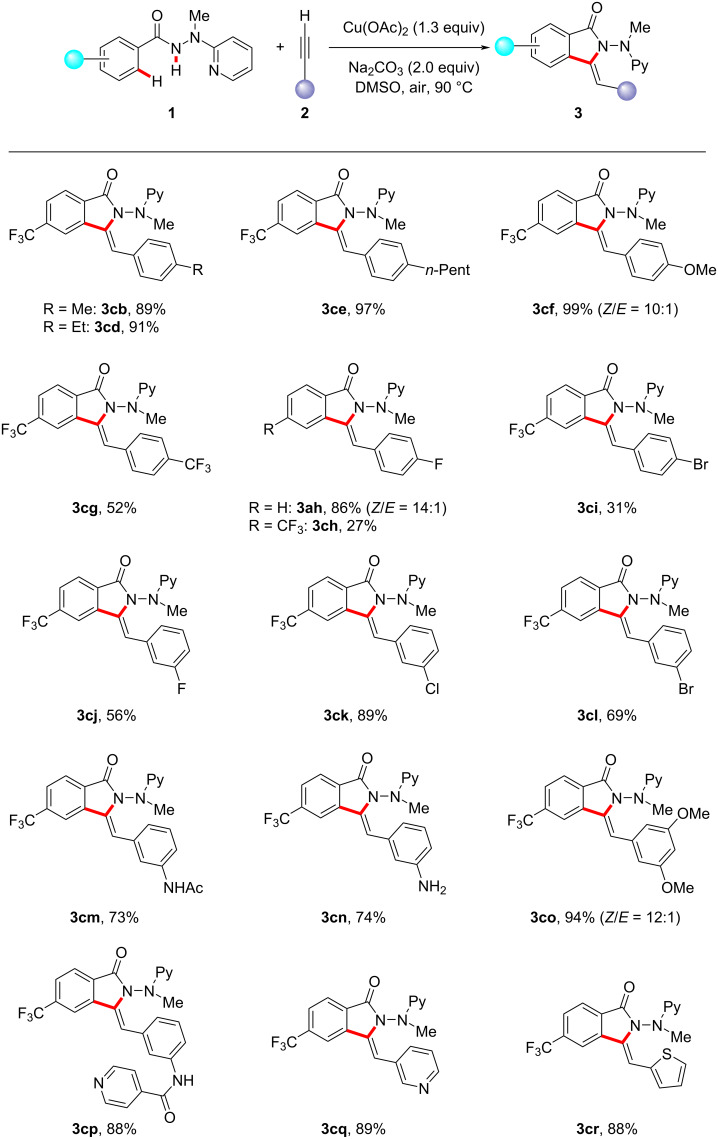
Copper-mediated oxidative C−H/N−H functionalization of **1** with alkynes **2**.

We further investigated the viable scope of differently substituted terminal alkynes **2** as the general coupling partners for this transformation. As shown in Scheme 2, a variety of valuable electrophilic substitutes were well tolerated. Moreover, substrates with a highly reactive unprotected amino group also delivered the corresponding product **3cn** with good yield. The robustness of this protocol was further highlighted by the excellent reactivity of heterocyclic acetylenes (see **2p**–**r**). However, a complex mixture was observed when an aliphatic terminal alkyne was used, and no annulation product was detected for internal alkynes.

**Scheme 3:**
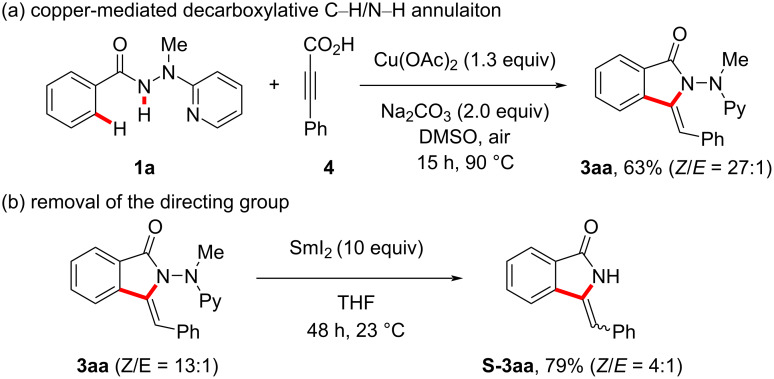
Decaboxylative C−H/N−H activation and cleavage of the directing group.

Our copper-promoted C−H annulation protocol was not restricted to terminal alkynes. Under identical reaction conditions, commercially available alkynylcarboxylic acid **4** also proved to be a viable substrate. Thus, the corresponding isoindolone **3aa** was assembled via a tandem decarboxylative C−H/C−C sequence (Scheme 3a). The practical relevance of our approach was reflected by the cleavage of the *N*-2-pyridylhydrazide group, yielding **S-3aa** (Scheme 3b).

**Scheme 4:**
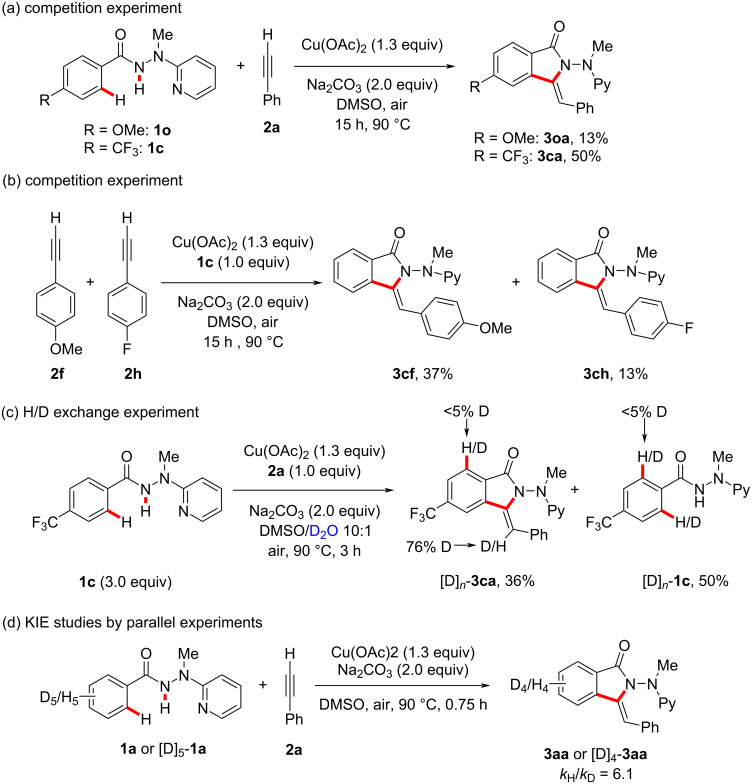
Summary of key mechanistic findings.

Inspired by the remarkable robustness of the copper-promoted C−H activations with alkynes, we became interested to explore the working mode by a set of experiments. To this end, electron-poor arenes inherently reacted preferentially in intermolecular competition experiments (Scheme 4a). This observation could be explained in terms of a concerted metalation deprotonation (CMD) mechanism [50]. Interestingly, electron-rich alkyne **2f** displayed a higher reactivity in the copper-promoted C−H activations as compared to the electron-poor analog **2h** (Scheme 4b). A significant H/D scrambling was not detected in the *ortho*-position of the reisolated benzhydrazide **1c** and product **3ca** when the reaction was conducted with the isotopically labeled D_2_O as cosolvent (Scheme 4c). This observation indicated that the C−H cleavage is irreversible. In accordance with this finding, a kinetic isotope effect (KIE) of *k*_H_/*k*_D_ ≈ 6.1 was observed by parallel experiments, again suggesting that the C‒H activation is kinetically relevant (Scheme 4d).

**Scheme 5:**
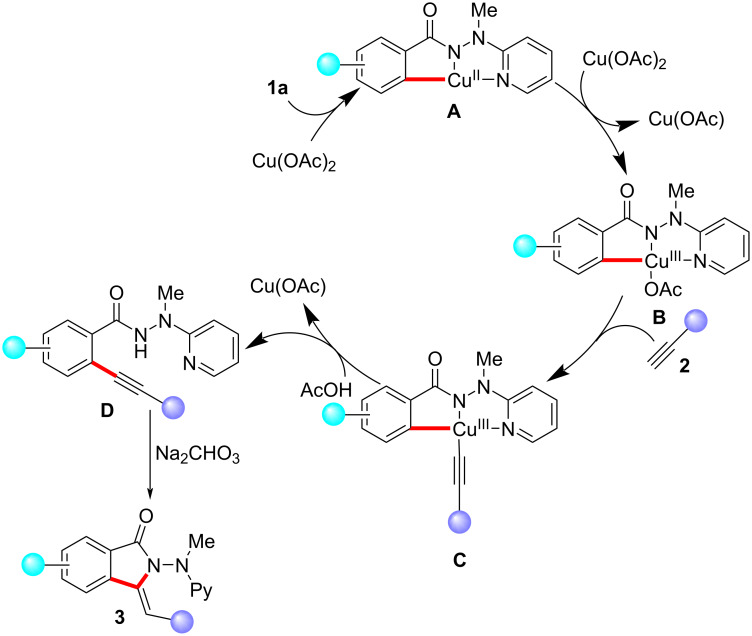
Proposed reaction pathway.

Based on our mechanistic findings and previous studies, we propose a tentative plausible reaction pathway in Scheme 5. The transformation commences with substrate coordination and subsequent carboxylate-assisted C−H cleavage to deliver copper(II) intermediate **A**. Next, the copper(III) carboxylate species **B** is generated. Thereafter, a facile base-assisted ligand exchange is followed by reductive elimination to afford the alkynylated benzamide **D**. Finally, the desired isoindolone **3** is formed via an intramolecular hydroamination in the presence of base.

## Conclusion

In conclusion, we have reported on the chelation-assisted oxidative copper-promoted cascade C−H alkynylation and intramolecular annulation. The removable *N*-2-pyridylhydrazide was utilized to facilitate copper(II)-promoted C−H activations. Thus, the robust copper-mediated C−H activation featured remarkable compatibility of synthetically meaningful functional groups, giving facile access to valuable 3-methyleneisoindolin-1-one scaffolds.

## Experimental

### General information

Yields refer to isolated compounds, estimated to be >95% pure as determined by ^1^H NMR spectroscopy. Chromatographic separation was carried out on silica gel 60H (200–300 mesh) manufactured by Qingdao Haiyang Chemical Group Co. (China). High-resolution mass spectrometry (HRMS) was measured on a Thermo-DFS mass spectrometer. NMR spectra were recorded on a JEOL 600 NMR device (^1^H: 600 MHz; ^13^C: 150 MHz; ^19^F: 565 MHz) in CDCl_3_. If not otherwise specified, the chemical shift (δ) is given in ppm.

### Materials

Reactions were carried out under an argon atmosphere using predried glassware, if not noted otherwise. Benzhydrazides **1** were synthesized according to a previously described method [36,44]. Other chemicals were obtained from commercial sources and were used without further purification.

### General procedure for the copper-promoted oxidative C−H/N−H activation with alkynes

To a 25 mL Schlenk tube were added benzhydrazide **1** (0.30 mmol, 1.00 equiv), the alkyne (0.90 mmol, 3.0 equiv), Cu(OAc)_2_ (71 mg, 0.39 mmol, 1.30 equiv), and Na_2_CO_3_ (64 mg, 0.60 mmol, 2.00 equiv) under an air atmosphere. The mixture was stirred at 90 °C for 15 h. At ambient temperature, H_2_O (15 mL) and Et_3_N (0.5 mL) were added, and a suspension was formed immediately. After filtrated through a Celite^®^ pad, the reaction mixture was extracted with EtOAc (3 × 20 mL). The combined organic phase was washed with brine (20 mL) and dried over Na_2_SO_4_. Then, Et_3_N (0.5 mL) and silica gel (0.8 g) were added, and the combined solvent was removed under reduced pressure. The residue solid sample was purified by column chromatography on silica gel (petroleum/EtOAc 5:1 to 2:1, with 1% Et_3_N), yielding the desired product **3**.

### (*Z*)-3-Benzylidene-2-(methyl[pyridin-2-yl]amino)isoindolin-1-one (**3aa**)

The general procedure was followed using hydrazide **1a** (68.2 mg, 0.30 mmol) and alkyne **2a** (91.9 mg, 0.90 mmol). Purification by column chromatography on silica gel (petroleum/EtOAc 20:1, with 1% Et_3_N) yielded **3aa** (87.4 mg, 89%, *Z*/*E* = 13:1) as a light yellow solid. mp 67–68 °C; ^1^H NMR (CDCl_3_, 600 MHz) δ 8.13 (ddd, *J* = 5.0; 1.9; 0.9 Hz, 1H), 7.90 (dd, *J* = 7.6; 1.0 Hz, 1H), 7.85–7.82 (m, 1H), 7.70 (d, *J* = 1.2 Hz, 1H), 7.56 (dd, *J* = 7.6; 0.9 Hz, 1H), 7.44 (ddd, *J* = 8.8; 7.1; 1.9 Hz, 1H), 7.17–7.05 (m, 5H), 6.85 (d, *J* = 0.9 Hz, 1H), 6.67 (ddd, *J* = 7.2; 5.0; 0.9 Hz, 1H), 6.44–6.41 (m, 1H), 3.01 (s, 3H); ^13^C{^1^H} NMR (CDCl_3_, 150 MHz) δ 165.7 (C_q_), 157.6 (C_q_), 147.7 (CH), 137.4 (CH), 136.2 (C_q_), 133.2 (C_q_), 132.8 (CH), 132.1 (C_q_), 129.3 (CH), 128.7 (CH), 127.3 (CH), 127.3 (CH), 126.5 (C_q_), 123.8 (CH), 119.8 (CH), 114.3 (CH), 107.8 (CH), 106.4 (CH), 36.7 (CH_3_); HRESIMS (*m*∕*z*): [M + H]^+^ calcd for C_21_H_18_N_3_O, 328.1444; found, 328.1439.

## Supporting Information

File 1 Characterization data for **3** and copies of ^1^H, ^13^C, and ^19^F NMR spectra.
