# Supplementary material for: Copper-mediated oxidative C−H/N−H activations with alkynes by removable hydrazides
Source: Beilstein J Org Chem. 2021 Jul 8;17:1591–9. doi: 10.3762/bjoc.17.113 (PMC8275871; doi:10.3762/bjoc.17.113)
Supplement: File 1 — Characterization data for 3 and copies of 1H, 13C, and 19F NMR spectra. [file Beilstein_J_Org_Chem-17-1591-s001.pdf]

BEILSTEIN JOURNAL OF ORGANIC CHEMISTRY

Supporting Information

for

Copper-mediated oxidative C−H/N−H activations with alkynes by removable hydrazides

Feng Xiong, Bo Li, Chenrui Yang, Liang Zou, Wenbo Ma, Linghui Gu, Ruhuai Mei and Lutz Ackermann

Beilstein J. Org. Chem. **2021,** *17,* 1591–1599. doi:10.3762/bjoc.17.113

Characterization data for 3 and copies of ^{1}H, ^{13}C, and ^{19}F NMR spectra

License and Terms: This is a supporting information file under the terms of the Creative Commons Attribution License (https://creativecommons.org/licenses/by/4.0). Please note that the reuse, redistribution and reproduction in particular requires that the author(s) and source are credited and that individual graphics may be subject to special legal provisions.
The license is subject to the *Beilstein Journal of Organic Chemistry* terms and conditions: (https://www.beilstein-journals.org/bjoc/terms)

Table of contents

General remarks S-2

Optimization of the reaction conditions S-3

General procedure for the copper-promoted oxidative C−H/N−H activation S-5

Characterization data of products **3** S-6

Copper-mediated decarboxylative C−H/N−H activation S-29

Removal of the directing group S-30

Mechanistic studies S-31

References S-38

NMR spectra S-39

General remarks

Reactions were carried out under an argon atmosphere using predried glassware, if not noted otherwise. Benzhydrazides **1** were synthesized according to a previously described method [1,2]. Other chemicals were obtained from commercial sources and were used without further purification. Yields refer to isolated compounds, estimated to be >95% pure as determined by ^{1}H NMR. Chromatography separations were carried out on silica gel 60H (200–300 mesh) manufactured by Qingdao Haiyang Chemical Group Co. (China). High-resolution mass spectrometry (HRMS) was measured on Thermo-DFS mass spectrometer. NMR spectra were recorded on a JEOL 600 NMR spectrometer (^{1}H: 600 MHz; ^{13}C: 150 MHz; ^{19}F: 565 MHz) in $CDCl_3$. If not otherwise specified, chemical shifts (δ) are given in ppm.

Optimization of the reaction conditions

Table S-1: Optimization of the copper-promoted oxidative C−H/N−H activation with alkynes.[a]

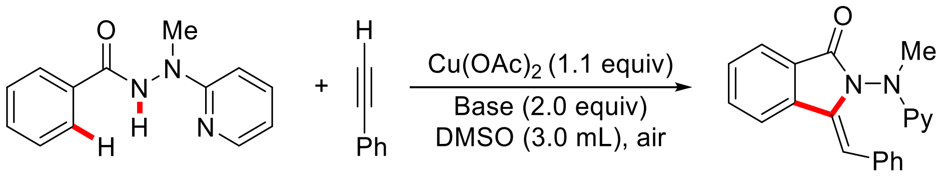

entry	solvent	base	T (°C)	*Z*/*E*	yield (%)
1	DMF	Na_2CO_3	90	—	trace
2	NMP	Na_2CO_3	90	—	trace
3	DMSO	Na_2CO_3	90	12:1	67
4	DMSO	Na_2CO_3	110	8:1	57
5	DMSO	Na_2CO_3	80	15:1	41
6	DMSO	Na_2CO_3	60	—	27
7	DMSO	NaOAc	90	—	25
8	DMSO	NaOPiv	90	—	30
9	DMSO	K_2CO_3	90	18:1	58
10	DMSO	Cs_2CO_3	90	20:1	44
11	DMSO	DBU	90	—	13
12	DMSO	Na_2CO_3	90	12:1	42[b]
13	DMSO	Na_2CO_3	90	9:1	83[c]
14	DMSO	Na_2CO_3	90	13:1	89[c,d]
15	DMSO	Na_2CO_3	90	12:1	86[d,e]
16	DMSO	—	90	—	trace
17	DMSO	Na_2CO_3	90	—	trace[e]
18	DMSO	Na_2CO_3	90	—	37[f]

[a]Reaction conditions: 25-mL Schlenk tube, **1a** (0.30 mmol), **2a** (0.90 mmol), $Cu(OAc)_2$ (1.1 equiv), base (2.0 equiv), solvent (3.0 mL), 15 h, under air. [b]$Cu(OAc)_2$ (0.8 equiv).

[c]$Cu(OAc)_2$ (1.3 equiv). [d]DMSO (6.0 mL). [e]$Cu(OAc)_2 \cdot H_2O$ (1.3 equiv). [f]Without $Cu(OAc)_2$. [g]Under N_2.

General procedure for the copper-promoted oxidative C−H/N−H activation with alkynes

To a 25-mL Schlenk tube were added benzhydrazide **1** (0.30 mmol, 1.00 equiv), alkyne **2** (0.90 mmol, 3.0 equiv), $Cu(OAc)_2$ (71 mg, 0.39 mmol, 1.30 equiv), and Na_2CO_3 (64 mg, 0.60 mmol, 2.00 equiv) under an air atmosphere. The mixture was stirred at 90 °C for 15 h. At ambient temperature, H_2O (15 mL) and Et_3N (0.5 mL) were added, and a suspension was formed immediately. After being filtered through a Celite® pad, the reaction mixture was extracted with EtOAc (3×20 mL). The combined organic phase was washed with brine (20 mL) and dried over Na_2SO_4. Then, Et_3N (0.5 mL) and silica gel (0.8 g) were added, and the combined solvent was removed under reduced pressure. The residue solid sample was purified by column chromatography on silica gel (petroleum/EtOAc 5:1 to 2:1, with 1% Et_3N), which yielded the desired product **3**.

Characterization data of 3

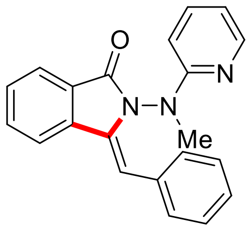

(*Z*)-3-Benzylidene-2-(methyl[pyridin-2-yl]amino)isoindolin-1-one (**3aa**)

The general procedure was followed using hydrazide **1a** (68.2 mg, 0.30 mmol) and alkyne **2a** (91.9 mg, 0.90 mmol). Purification by column chromatography on silica gel (petroleum/EtOAc 20:1, with 1% Et_3N) yielded **3aa** (87.4 mg, 89%, *Z*/*E* = 13:1) as a light yellow solid. mp 67–68 °C. **^{1}H NMR** (600 MHz, $CDCl_3$) δ = 8.13 (ddd, *J* = 5.0, 1.9, 0.9 Hz, 1H), 7.90 (dd, *J* = 7.6, 1.0 Hz, 1H), 7.85–7.82 (m, 1H), 7.70 (d, *J* = 1.2 Hz, 1H), 7.56 (dd, *J* = 7.6, 0.9 Hz, 1H), 7.44 (ddd, *J* = 8.8, 7.1, 1.9 Hz, 1H), 7.17–7.05 (m, 5H), 6.85 (d, *J* = 0.9 Hz, 1H), 6.67 (ddd, *J* = 7.2, 5.0, 0.9 Hz, 1H), 6.44–6.41 (m, 1H), 3.01 (s, 3H). **^{13}C NMR** (150 MHz, $CDCl_3$) δ = 165.7 (C_q), 157.6 (C_q), 147.7 (CH), 137.4 (CH), 136.2 (C_q), 133.2 (C_q), 132.8 (CH), 132.1 (C_q), 129.3 (CH), 128.7 (CH), 127.3 (CH), 127.3 (CH), 126.5 (C_q), 123.8 (CH), 119.8 (CH), 114.3 (CH), 107.8 (CH), 106.4 (CH), 36.7 (CH_3). **HR-MS** (ESI) *m/z* calcd for $C_{21}H_{18}N_3O$ [M+H$^+$] 328.1444, found 328.1439.

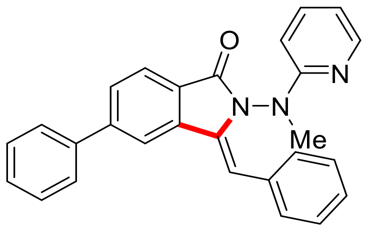

(*Z*)-3-Benzylidene-2-(methyl[pyridin-2-yl]amino)-5-phenylisoindolin-1-one (3ba)

The general procedure was followed using hydrazide **1b** (91.0 mg, 0.30 mmol) and alkyne **2a** (91.9 mg, 0.90 mmol). Purification by column chromatography on silica gel (petroleum/EtOAc 10:1) yielded **3ba** (65.3 mg, 54%, *Z*/*E* = 29:1) as a light yellow solid.

mp 135–136 °C. **^{1}H NMR** (600 MHz, $CDCl_3$) δ = 8.15 (ddd, J = 5.0, 1.9, 0.9 Hz, 1H), 8.01 (dd, J = 1.6, 0.7 Hz, 1H), 7.97 (dd, J = 7.9, 0.7 Hz, 1H), 7.77 (dd, J = 7.9, 1.5 Hz, 1H), 7.71–7.68 (m, 2H), 7.55–7.51 (m, 2H), 7.48–7.44 (m, 2H), 7.19–7.07 (m, 5H), 6.92 (s, 1H), 6.68 (ddd, J = 7.1, 5.0, 0.9 Hz, 1H), 6.46 (dt, J = 8.5, 0.9 Hz, 1H), 3.03 (s, 3H). **^{13}C NMR** (150 MHz, $CDCl_3$) δ = 165.6 (C_q), 157.6 (C_q), 147.8 (CH), 146.3 (C_q), 140.2 (C_q), 137.5 (CH), 136.9 (C_q), 133.2 (C_q), 132.2 (C_q), 129.0 (CH), 128.8 (CH), 128.6 (CH), 128.4 (CH), 127.4 (CH), 127.4 (CH), 127.3 (CH), 125.3 (C_q), 124.2 (CH), 118.5 (CH), 114.3 (CH), 107.9 (CH), 106.5 (CH), 36.7 (CH_3). **HR-MS** (ESI) m/z calcd for $C_{27}H_{22}N_3O$ [M+H$^+$] 404.1757, found 404.1755.

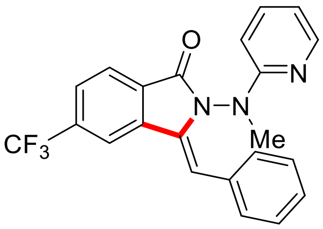

(*Z*)-3-Benzylidene-2-(methyl[pyridin-2-yl]amino)-5-(trifluoromethyl)isoindolin-1-one (3ca)

The general procedure was followed using hydrazide **1c** (88.6 mg, 0.30 mmol) and alkyne **2a** (91.0 mg, 0.90 mmol). Purification by column chromatography on silica gel (petroleum/EtOAc 10:1) yielded **3ca** (117.4 mg, 99%, *Z*/*E* = 43:1) as a light yellow solid. mp 121–122 °C. **^{1}H NMR** (600 MHz, $CDCl_3$) δ = 8.14 (ddd, J = 5.0, 1.9, 0.9 Hz, 1H), 8.10 (d, J = 1.5 Hz, 1H), 8.03 (d, J = 7.9 Hz, 1H), 7.83–7.79 (m, 1H), 7.46 (ddd, J = 8.7, 7.1, 1.9 Hz, 1H), 7.19–7.15 (m, 1H), 7.13–7.06 (m, 4H), 6.94 (s, 1H), 6.70 (ddd, J = 7.2, 5.0, 0.9 Hz, 1H), 6.38 (dd, J = 8.5, 1.0 Hz, 1H), 3.02 (s, 3H). **^{13}C NMR** (150 MHz, $CDCl_3$) δ = 164.5 (C_q), 157.1 (C_q), 147.9 (CH), 137.6 (CH), 136.5 (C_q), 134.7 (q, $^2J_{C\text{-}F}$ = 32.6 Hz, C_q), 132.7 (C_q), 131.4 (C_q), 129.3 (C_q), 128.7 (CH), 127.7 (CH), 127.4 (CH), 126.0 (q, $^3J_{C\text{-}F}$ = 3.8 Hz, CH), 124.5 (CH), 123.6 (q, $^1J_{C\text{-}F}$ = 271.0 Hz, C_q), 117.3 (q, $^3J_{C\text{-}F}$ = 4.0 Hz, CH), 114.7 (CH), 109.7 (CH), 106.3 (CH), 36.9 (CH_3). **^{19}F NMR** (565 MHz, $CDCl_3$) δ = -62.49. **HR-MS** (ESI) m/z calcd for $C_{22}H_{17}F_3N_3O$ [M+H$^+$] 396.1318, found 396.1316.

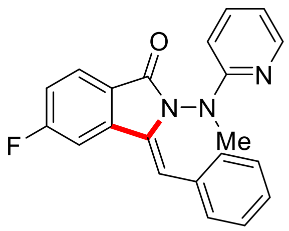

(*Z*)-3-Benzylidene-5-fluoro-2-(methyl[pyridin-2-yl]amino)isoindolin-1-one (3da)

The general procedure was followed using hydrazide **1d** (73.6 mg, 0.30 mmol) and alkyne **2a** (91.0 mg, 0.90 mmol). Purification by column chromatography on silica gel (petroleum/EtOAc 10:1) yielded **3da** (91.2 mg, 88%, *Z*/*E* = 8:1) as a light yellow solid. mp 119–120 °C. **^{1}H NMR** (600 MHz, $CDCl_3$) δ = 8.13 (ddd, J = 5.0, 1.9, 0.9 Hz, 1H), 7.89 (dd, J = 8.4, 4.9 Hz, 1H), 7.51–7.42 (m, 3H), 7.19–7.14 (m, 1H), 7.11 –7.07 (m, 4H), 6.80 (s, 1H), 6.68 (ddd, J = 7.1, 5.0, 0.9 Hz, 1H), 6.41 (dd, J = 8.5, 1.0 Hz, 1H), 3.00 (s, 3H). **^{13}C NMR** (150 MHz, $CDCl_3$) δ = 166.0 (d, $^1J_{C\text{-}F}$ = 252.7 Hz, C_q), 164.9 (C_q), 157.4 (C_q), 147.8 (CH), 138.7 (d, $^3J_{C\text{-}F}$ = 10.3 Hz, C_q), 137.5 (CH), 132.8 (C_q), 131.5 (d, $^4J_{C\text{-}F}$ = 3.5 Hz, C_q), 128.8 (CH), 127.6 (CH), 127.3 (CH), 126.2 (d, $^3J_{C\text{-}F}$ = 10.0 Hz, CH), 122.7 (C_q), 117.3 (d, $^2J_{C\text{-}F}$ = 23.8 Hz, CH), 114.5 (CH), 108.9 (CH), 107.0 (d, $^2J_{C\text{-}F}$ = 24.7 Hz, CH), 106.4 (CH), 36.8 (CH_3). **^{19}F NMR** (565 MHz, $CDCl_3$) δ = -(104.47–104.43, m, 1F). **HR-MS** (ESI) *m/z* calcd for $C_{21}H_{17}N_3O$ [M+H$^+$] 346.1346, found 346.1350.

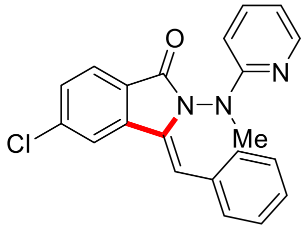

(*Z*)-3-Benzylidene-5-chloro-2-(methyl[pyridin-2-yl]amino)isoindolin-1-one (3ea)

The general procedure was followed using hydrazide **1e** (78.5 mg, 0.30 mmol) and alkyne **2a** (91.0 mg, 0.90 mmol). Purification by column chromatography on silica gel (petroleum/EtOAc 10:1) yielded **3ea** (97.7 mg, 90%, *Z*/*E* = 19:1) as a light yellow solid. mp 131–132 °C. **^{1}H NMR** (600 MHz, $CDCl_3$) δ = 8.13 (ddd, J = 5.0, 1.9, 0.9 Hz, 1H),

7.85–7.80 (m, 2H), 7.52 (dd, J = 8.1, 1.7 Hz, 1H), 7.45 (ddd, J = 8.7, 7.2, 1.9 Hz, 1H), 7.18–7.13 (m, 1H), 7.09 (d, J = 5.8 Hz, 4H), 6.82 (s, 1H), 6.70–6.66 (m, 1H), 6.39 (dd, J = 8.5, 1.0 Hz, 1H), 3.00 (s, 3H). **^{13}C NMR** (150 MHz, $CDCl_3$) δ = 164.9 (C_q), 157.3 (C_q), 147.8 (CH), 139.4 (C_q), 137.7 (C_q), 137.5 (CH), 132.8 (C_q), 131.3 (C_q), 129.7 (CH), 128.8 (CH), 128.8 (CH), 127.6 (CH), 127.3 (CH), 127.3 (CH), 125.1 (CH), 124.9 (C_q), 120.2 (CH), 114.5 (CH), 109.0 (CH), 106.3 (CH), 36.8 (CH_3). **HR-MS** (ESI) *m/z* calcd for $C_{21}H_{17}{}^{35}ClN_3O$ [M+H$^+$] 362.1055, found362.1054.

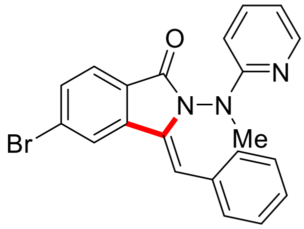

(*Z*)-3-Benzylidene-5-bromo-2-(methyl[pyridin-2-yl]amino)isoindolin-1-one (3fa)

The general procedure was followed using hydrazide **1f** (91.8 mg, 0.30 mmol) and alkyne **2a** (91.0 mg, 0.90 mmol). Purification by column chromatography on silica gel (petroleum/EtOAc 10:1) yielded **3fa** (108.5 mg, 89%, *Z*/*E* = 25:1) as a light yellow solid. mp 140–141 °C. **^{1}H NMR** (600 MHz, $CDCl_3$) δ = 8.13 (ddd, J = 5.0, 2.1, 1.0 Hz, 1H), 7.98 (d, J = 1.3 Hz, 1H), 7.76 (dd, J = 8.1, 1.1 Hz, 1H), 7.68 (dd, J = 8.1, 1.4 Hz, 1H), 7.45 (ddd, J = 8.4, 7.2, 1.5 Hz, 1H), 7.18–7.13 (m, 1H), 7.12–7.05 (m, 4H), 6.82 (d, J = 1.2 Hz, 1H), 6.68 (ddd, J = 7.1, 5.0, 1.1 Hz, 1H), 6.39 (dd, J = 8.5, 1.0 Hz, 1H), 2.99 (s, 3H). **^{13}C NMR** (150 MHz, $CDCl_3$) δ = 165.0 (C_q), 157.3 (C_q), 147.8 (CH), 137.9 (C_q), 137.5 (CH), 132.8 (C_q), 132.5 (CH), 131.1 (C_q), 128.8 (CH), 128.8 (CH), 127.7 (C_q), 127.6 (CH), 127.4 (CH), 127.4 (CH), 125.3 (C_q), 125.2 (CH), 123.2 (CH), 114.5 (CH), 109.1 (CH), 106.4 (CH), 36.8 (CH_3). **HR-MS** (ESI) *m/z* calcd for $C_{21}H_{17}BrN_3O$ [M+H$^+$] 406.0550, found 406.0552.

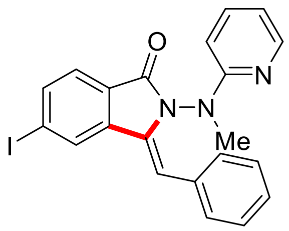

(*Z*)-3-Benzylidene-5-iodo-2-(methyl[pyridin-2-yl]amino)isoindolin-1-one (3ga)

The general procedure was followed using hydrazide **1g** (105.9 mg, 0.30 mmol) and alkyne **2a** (91.0 mg, 0.90 mmol). Purification by column chromatography on silica gel (petroleum/EtOAc 10:1) yielded **3ga** (81.6 mg, 60%, *Z*/*E* = 9:1) as a light yellow solid. mp 132–133 °C. **^{1}H NMR** (600 MHz, $CDCl_3$) δ = 8.19 (d, *J* = 1.3 Hz, 1H), 8.12 (ddd, *J* = 5.0, 2.0, 0.9 Hz, 1H), 7.88 (dd, *J* = 8.0, 1.3 Hz, 1H), 7.61 (d, *J* = 8.0 Hz, 1H), 7.47–7.40 (m, 1H), 7.15 (p, *J* = 4.3 Hz, 1H), 7.07 (d, *J* = 4.5 Hz, 4H), 6.80 (s, 1H), 6.69–6.65 (m, 1H), 6.37 (d, *J* = 8.5 Hz, 1H), 2.98 (s, 3H). **^{13}C NMR** (150 MHz, $CDCl_3$) δ = 165.2 (C_q), 157.3 (C_q), 147.8 (CH), 138.3 (CH), 137.7 (C_q), 137.5 (CH), 132.9 (C_q), 130.9 (C_q), 129.1 (CH), 128.7 (CH), 127.6 (CH), 127.3 (CH), 125.8 (C_q), 125.1 (CH), 114.5 (CH), 109.0 (CH), 106.3 (CH), 99.8 (C_q), 36.8 (CH_3). **HR-MS** (ESI) *m/z* calcd for $C_{21}H_{17}IN_3O$ [M+H$^+$] 454.0411, found 454.0408.

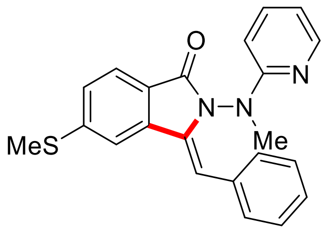

(*Z*)-3-Benzylidene-2-(methyl(pyridin-2-yl)amino)-5-(methylthio)isoindolin-1-one (3ha)

The general procedure was followed using hydrazide **1h** (82.0 mg, 0.30 mmol) and alkyne **2a** (91.0 mg, 0.90 mmol). Purification by column chromatography on silica gel (petroleum/EtOAc 10:1) yielded **3ha** (88.5 mg, 79%, *Z*/*E* = 14:1) as a light yellow solid. mp 71–72 °C. **^{1}H NMR** (600 MHz, $CDCl_3$) δ = 8.70 (s, 1H), 8.36 (s, 1H), 8.10 (d, *J* = 4.5 Hz, 1H), 7.68 (d, *J* = 8.2 Hz, 1H), 7.45 – 7.41 (m, 1H), 7.34 (dd, *J* = 8.3, 1.2 Hz,

1H), 7.14 – 7.08 (m, 1H), 7.07 – 7.01 (m, 4H), 6.79 (s, 1H), 6.66 (dd, *J* = 6.8, 5.5 Hz, 1H), 6.39 (d, *J* = 8.4 Hz, 1H), 2.95 (s, 3H), 2.09 (s, 3H). **^{13}C NMR** (150 MHz, $CDCl_3$) δ = 169.3 (C_q), 165.8 (C_q), 157.5 (C_q), 147.6 (CH), 143.0 (C_q), 137.7 (CH), 133.1 (C_q), 131.9 (C_q), 128.8 (CH), 128.8 (CH), 127.4 (CH), 127.3 (CH), 127.3 (CH), 124.3 (CH), 121.4 (C_q), 120.4 (CH), 114.5 (CH), 110.4 (CH), 108.6 (CH), 106.5 (CH), 36.8 (CH_3), 24.5 (CH_3). **HR-MS** (ESI) *m/z* calcd for $C_{22}H_{20}N_3OS$ [M+H$^+$] 374.1322, found 374.1319.

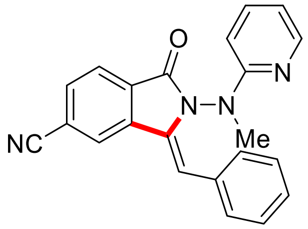

(*Z*)-3-Benzylidene-2-(methyl[pyridin-2-yl]amino)-1-oxoisoindoline-5-carbonitrile (3ia)

The general procedure was followed using hydrazide **1i** (75.7 mg, 0.30 mmol) and alkyne **2a** (91.0 mg, 0.90 mmol). Purification by column chromatography on silica gel (petroleum/EtOAc 10:1) yielded **3ia** (100.4 mg, 95%, *Z*/*E* = 19:1) as a light yellow solid. mp 168–169 °C. **^{1}H NMR** (600 MHz, $CDCl_3$) δ = 8.12 (dd, *J* = 4.7, 1.5 Hz, 2H), 8.00 (dd, *J* = 7.8, 0.8 Hz, 1H), 7.80 (dd, *J* = 7.8, 1.3 Hz, 1H), 7.45 (ddd, *J* = 8.7, 7.2, 1.9 Hz, 1H), 7.19–7.14 (m, 1H), 7.11–7.09 (m, 4H), 6.90 (s, 1H), 6.70 (ddd, *J* = 7.2, 5.0, 0.9 Hz, 1H), 6.35 (dd, *J* = 8.5, 0.9 Hz, 1H), 3.00 (s, 3H). **^{13}C NMR** (150 MHz, $CDCl_3$) δ = 164.1 (C_q), 157.0 (C_q), 147.9 (CH), 137.6 (CH), 136.6 (C_q), 132.5 (C_q), 132.3 (CH), 130.8 (C_q), 129.7 (C_q), 128.8 (CH), 127.9 (CH), 127.4 (CH), 124.7 (CH), 124.1 (CH), 118.0 (C_q), 116.2 (C_q), 114.8 (CH), 110.4 (CH), 106.3 (CH), 37.0 (CH_3). **HR-MS** (ESI) *m/z* calcd for $C_{22}H_{17}N_4O$ [M+H$^+$] 353.1397, found 353.1395.

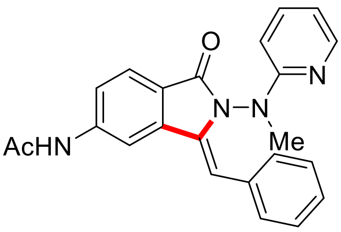

(*Z*)-*N*-{3-Benzylidene-2-(methyl[pyridin-2-yl]amino)-1-oxoisoindolin-5-yl}acetamide (3ja)

The general procedure was followed using hydrazide **1j** (85.3 mg, 0.30 mmol) and alkyne **2a** (91.0 mg, 0.90 mmol). Purification by column chromatography on silica gel (petroleum/EtOAc 10:1) yielded **3ja** (98.0 mg, 85%, *Z*/*E* = 9:1) as a light yellow solid. mp 74–75 °C. **^{1}H NMR** (600 MHz, $CDCl_3$) δ = 8.12 (ddd, *J* = 5.0, 2.1, 1.0 Hz, 1H), 7.77 (d, *J* = 7.9 Hz,1H), 7.62 (d, *J* = 1.5 Hz, 1H), 7.49–7.40 (m, 2H), 7.38 (dd, *J* = 8.1, 1.4 Hz, 1H), 7.17–7.12 (m, 1H), 7.11–7.05 (m, 4H), 6.82 (s, 1H), 6.70–6.64 (m, 1H), 6.42 (dd, *J* = 8.5, 1.0 Hz, 1H), 2.99 (s, 3H), 2.61 (s, 3H). **^{13}C NMR** (150 MHz, $CDCl_3$) δ = 165.5 (C_q), 165.5 (C_q), 157.6 (C_q), 147.7 (CH), 145.8 (C_q), 137.5 (CH), 136.9 (C_q), 133.2 (C_q), 131.8 (C_q), 128.8 (CH), 127.4 (CH), 127.3 (CH), 126.5 (CH), 123.9 (CH), 123.0 (C_q), 116.1 (CH), 114.3 (CH), 107.9 (CH), 106.4 (CH), 36.7 (CH_3), 15.3 (CH_3). **HR-MS** (ESI) *m/z* calcd for $C_{23}H_{21}N_4O_2$ [M+H$^+$] 385.1659, found 385.1656.

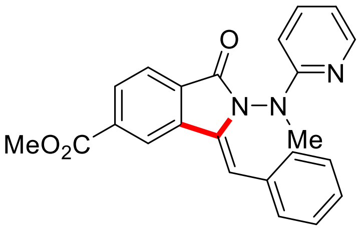

Methyl-(*Z*)-3-benzylidene-2-(methyl[pyridin-2-yl]amino)-1-oxoisoindoline-5-carboxylate (3ka)

The general procedure was followed using hydrazide **1k** (85.6 mg, 0.30 mmol) and alkyne **2a** (91.0 mg, 0.90 mmol). Purification by column chromatography on silica gel (petroleum/EtOAc 10:1) yielded **3ka** (80.9 mg, 70%, *Z*/*E* = 14:1) as a light yellow solid. mp 155–156 °C. **^{1}H NMR** (600 MHz, $CDCl_3$) δ = 8.51 (s, 1H), 8.20 (dd, *J* = 7.9, 1.3 Hz, 1H), 8.12 (ddd, *J* = 5.0, 1.9, 0.9 Hz, 1H), 7.95 (dd, *J* = 7.9, 0.8 Hz, 1H), 7.43 (ddd, *J* = 8.5, 7.2, 1.9 Hz, 1H), 7.18–7.12 (m, 1H), 7.13–7.05 (m, 6H), 6.94 (s, 1H), 6.67 (ddd,

J = 7.2, 5.0, 0.9 Hz, 1H), 6.38 (d, J = 8.5 Hz, 1H), 3.99 (s, 3H), 3.00 (s, 3H). **^{13}C NMR** (150 MHz, $CDCl_3$) δ = 166.1 (C_q), 164.8 (C_q), 157.3 (C_q), 147.8 (CH), 137.5 (CH), 136.2 (C_q), 134.1 (C_q), 132.9 (C_q), 131.6 (C_q), 130.1 (CH), 129.9 (C_q), 128.8 (CH), 127.6 (CH), 127.3 (CH), 123.8 (CH), 121.4 (CH), 114.5 (CH), 109.1 (CH), 106.3 (CH), 52.7 (CH_3), 36.8 (CH_3). **HR-MS** (ESI) *m/z* calcd for $C_{23}H_{20}N_3O_3$ [M+H$^+$] 386.1499, found 386.1498.

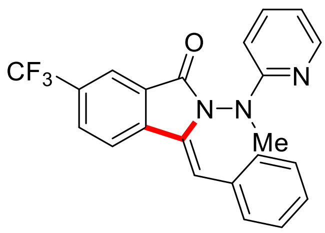

(*Z*)-3-Benzylidene-2-(methyl(pyridin-2-yl)amino)-6-(trifluoromethyl)isoindolin-1-one (3la)

The general procedure was followed using hydrazide **1l** (88.6 mg, 0.30 mmol) and alkyne **2a** (91.0 mg, 0.90 mmol). Purification by column chromatography on silica gel (petroleum/EtOAc 10:1) yielded **3la** (116.2 mg, 98%, *Z*/*E* = 13:1) as a light yellow solid. mp 119–120 °C. **^{1}H NMR** (600 MHz, $CDCl_3$) δ = 8.17 (s, 1H), 8.13 (dd, J = 5.0, 1.5 Hz, 1H), 7.96–7.91 (m, 2H), 7.47–7.42 (m, 1H), 7.20–7.14 (m, 1H), 7.12–7.05 (m, 4H), 6.94 (s, 1H), 6.69 (dd, J = 7.3, 4.9 Hz, 1H), 6.38 (d, J = 8.4 Hz, 1H), 3.01 (s, 3H). **^{13}C NMR** (150 MHz, $CDCl_3$) δ = 164.5 (C_q), 157.2 (C_q), 147.9 (CH), 139.1 (C_q), 137.6 (CH), 132.7 (C_q), 131.5 (q, $^2J_{C-F}$ = 35.1 Hz, C_q), 131.4 (C_q), 129.5 (q, $^3J_{C-F}$ = 3.8 Hz, CH), 128.8 (CH), 127.8 (CH), 127.4 (CH), 127.0 (C_q), 123.6 (q, $^1J_{C-F}$ = 272.7 Hz, C_q), 121.2 (q, $^3J_{C-F}$ = 4.0 Hz, CH), 120.5 (CH), 114.7 (CH), 110.3 (CH), 106.3 (CH), 36.9 (CH_3). **^{19}F NMR** (565 MHz, $CDCl_3$) δ = -62.27 (s, 3F). **HR-MS** (ESI) *m/z* calcd for $C_{22}H_{17}F_3N_3O$ [M+H$^+$] 396.1318, found 396.1314.

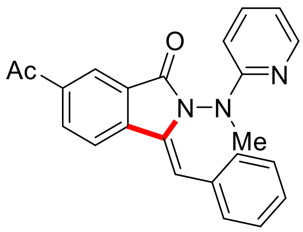

(*Z*)-6-Acetyl-3-benzylidene-2-(methyl(pyridin-2-yl)amino)isoindolin-1-one (3ma)

The general procedure was followed using hydrazide **1m** (80.8 mg, 0.30 mmol) and alkyne **2a** (91.0 mg, 0.90 mmol). Purification by column chromatography on silica gel (petroleum/EtOAc 10:1) yielded **3ma** (70.9 mg, 64%, *Z*/*E* = 15:1) as a light yellow solid. mp 132–133 °C. **^{1}H NMR** (600 MHz, $CDCl_3$) δ = 8.43 (s, 1H), 8.32 (dd, *J* = 8.2, 1.3 Hz, 1H), 8.13 (dd, *J* = 4.7, 1.5 Hz, 1H), 7.92 (d, *J* = 8.1 Hz, 1H), 7.47 – 7.41 (m, 1H), 7.16 (t, *J* = 6.9 Hz, 1H), 7.13 – 7.05 (m, 4H), 6.94 (s, 1H), 6.69 (dd, *J* = 6.9, 5.1 Hz, 1H), 6.40 (d, *J* = 8.4 Hz, 1H), 3.01 (s, 3H), 2.69 (s, 3H). **^{13}C NMR** (150 MHz, $CDCl_3$) δ = 196.8 (C_q), 165.1 (C_q), 157.3 (C_q), 147.9 (CH), 140.0 (C_q), 137.7 (C_q), 137.6 (CH), 132.8 (C_q), 132.2 (CH), 131.7 (C_q), 128.8 (CH), 128.8 (CH), 127.8 (CH), 127.4 (CH), 127.4 (CH), 126.8 (C_q), 124.3 (CH), 120.3 (CH), 114.6 (CH), 110.5 (CH), 106.4 (CH), 36.9 (CH_3), 26.8 (CH_3). **HR-MS** (ESI) *m/z* calcd for $C_{23}H_{20}N_3O_2$ [M+H^{+}] 370.1550, found 370.1548.

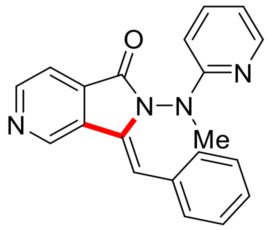

(*Z*)-3-Benzylidene-2-(methyl[pyridin-2-yl]amino)-2,3-dihydro-1*H*-pyrrolo[3,4-*c*]pyridin-1-one (3na)

The general procedure was followed using hydrazide **1n** (68.5 mg, 0.30 mmol) and alkyne **2a** (91.0 mg, 0.90 mmol). Purification by column chromatography on silica gel (petroleum/EtOAc 10:1) yielded **3na** (68.0 mg, 69%, *Z*/*E* = 27:1) as a light yellow solid. mp 143–144 °C. **^{1}H NMR** (600 MHz, $CDCl_3$) δ = 9.21 (d, *J* = 1.2 Hz, 1H), 8.83 (d, *J* = 5.0 Hz, 1H), 8.24–8.08 (m, 1H), 7.76 (dd, *J* = 5.0, 1.2 Hz, 1H), 7.44 (ddd, *J* = 8.8, 7.2, 1.9 Hz, 1H), 7.16 (tt, *J* = 6.4, 2.0 Hz, 1H), 7.09 (h, *J* = 6.0 Hz, 4H), 6.97 (s, 1H),

6.69 (dd, J = 7.2, 5.0 Hz, 1H), 6.35 (d, J = 8.5 Hz, 1H), 3.00 (s, 3H). **^{13}C NMR** (150 MHz, $CDCl_3$) δ = 164.2 (C_q), 157.0 (C_q), 149.8 (CH), 147.9 (CH), 142.7 (CH), 137.6 (CH), 133.2 (C_q), 132.6 (C_q), 130.6 (C_q), 130.3 (C_q), 128.7 (CH), 127.7 (CH), 127.4 (CH), 117.0 (CH), 114.8 (CH), 110.3 (CH), 106.3 (CH), 36.9 (CH_3). **HR-MS** (ESI) *m/z* calcd for $C_{20}H_{17}N_4O$ [M+H$^+$] 329.1397, found 329.1391.

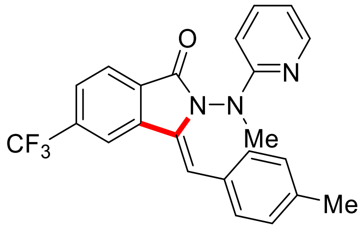

(*Z*)-2-[Methyl(pyridin-2-yl)amino]-3-(4-methylbenzylidene)-5-(trifluoromethyl)isoindolin-1-one (3cb)

The general procedure was followed using hydrazide **1c** (88.6 mg, 0.30 mmol) and alkyne **2b** (104.5 mg, 0.90 mmol). Purification by column chromatography on silica gel (petroleum/EtOAc 10:1) yielded **3cb** (109.3 mg, 89%) as a light yellow solid. mp 135–136 °C. **^{1}H NMR** (600 MHz, $CDCl_3$) δ = 8.16 (ddd, J = 5.0, 1.9, 0.9 Hz, 1H), 8.08 (s, 1H), 8.01 (d, J = 7.2 Hz, 1H), 7.78 (d, J = 9.3 Hz, 1H), 7.46 (ddd, J = 8.7, 7.1, 1.8 Hz, 1H), 7.02 (d, J = 7.9 Hz, 2H), 6.92–6.89 (m, 3H), 6.70 (ddd, J = 7.2, 5.0, 0.9 Hz, 1H), 6.42 (dd, J = 8.5, 0.9 Hz, 1H), 3.03 (s, 3H), 2.26 (s, 3H). **^{13}C NMR** (150 MHz, $CDCl_3$) δ = 164.5 (C_q), 157.4 (C_q), 147.9 (CH), 137.7 (C_q), 137.6 (CH), 136.7 (C_q), 134.6 (q, $^2J_{C\text{-}F}$ = 32.4 Hz, C_q), 130.8 (C_q), 129.6 (C_q), 129.2 (C_q), 128.9 (CH), 128.2 (CH), 125.8 (q, $^3J_{C\text{-}F}$ = 3.6 Hz, CH), 124.5 (CH), 123.6 (q, $^1J_{C\text{-}F}$ = 273.1 Hz, C_q), 117.2 (q, $^3J_{C\text{-}F}$ = 4.1 Hz, CH), 114.7 (CH), 110.1 (CH), 106.4 (CH), 36.9 (CH_3), 21.2 (CH_3). **^{19}F NMR** (565 MHz, $CDCl_3$) δ = -62.49 (s, 3F). **HR-MS** (ESI) *m/z* calcd for $C_{23}H_{19}F_3N_3O$ [M+H$^+$] 410.1475, found 410.1473.

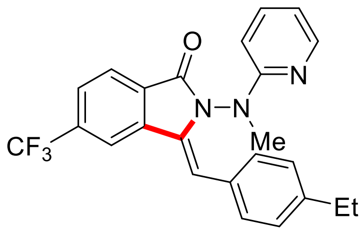

(*Z*)-3-(4-Ethylbenzylidene)-2-(methyl[pyridin-2-yl]amino)-5-(trifluoromethyl)isoindolin-1-one (3cd)

The general procedure was followed using hydrazide **1c** (88.6 mg, 0.30 mmol) and alkyne **2d** (117.2 mg, 0.90 mmol). Purification by column chromatography on silica gel (petroleum/EtOAc 10:1) yielded **3cd** (115.6 mg, 91%) as a light yellow solid. mp 119–120 °C. **^{1}H NMR** (600 MHz, $CDCl_3$) δ = 8.14 (ddd, J = 5.0, 1.9, 0.9 Hz, 1H), 8.08 (s, 1H), 8.01 (d, J = 8.0 Hz, 1H), 7.78 (dd, J = 8.0, 0.9 Hz, 1H), 7.45 (ddd, J = 8.5, 7.2, 1.9 Hz, 1H), 7.05 (d, J = 7.5 Hz, 2H), 6.94 – 6.90 (m, 3H), 6.69 (ddd, J = 7.1, 5.0, 0.9 Hz, 1H), 6.40 (d, J = 8.5 Hz, 1H), 3.04 (s, 3H), 2.56 (q, J = 7.6 Hz, 2H), 1.16 (t, J = 7.6 Hz, 3H). **^{13}C NMR** (150 MHz, $CDCl_3$) δ = 164.5 (C_q), 157.3 (C_q), 147.9 (CH), 144.0 (C_q), 137.6 (CH), 136.7 (C_q), 134.6 (q, $^2J_{C\text{-}F}$ = 32.5 Hz, C_q), 130.8 (C_q), 129.8 (C_q), 129.2 (C_q), 129.0 (CH), 126.9 (CH), 125.8 (q, $^3J_{C\text{-}F}$ = 3.7 Hz, CH), 124.5 (CH), 123.6 (q, $^1J_{C\text{-}F}$ = 273.0 Hz, C_q), 117.2 (q, $^3J_{C\text{-}F}$ = 4.0 Hz, CH), 114.6 (CH), 110.1 (CH), 106.4 (CH), 36.9 (CH_3), 28.5 (CH_2), 15.4 (CH_3). **^{19}F NMR** (565 MHz, $CDCl_3$) δ = -62.48 (s, 3F). **HR-MS** (ESI) *m/z* calcd for $C_{24}H_{21}F_3N_3O$ [M+H$^+$] 424.1631, found 424.1627.

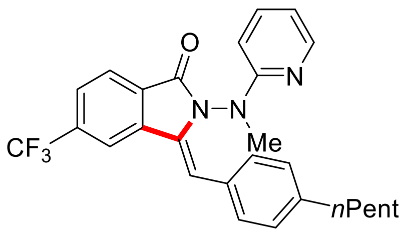

(*Z*)-2-(Methyl[pyridin-2-yl]amino)-3-(4-pentylbenzylidene)-5-(trifluoromethyl)isoindolin-1-one (3ce)

The general procedure was followed using hydrazide **1c** (88.6 mg, 0.30 mmol) and alkyne **2e** (155.1 mg, 0.90 mmol). Purification by column chromatography on silica gel (petroleum/EtOAc 10:1) yielded **3ce** (135.5 mg, 97%) as a light yellow solid. mp 71–72 °C. **^{1}H NMR** (600 MHz, $CDCl_3$) δ = 8.14 (ddd, J = 5.0, 1.9, 0.9 Hz, 1H), 8.08 (s,

1H), 8.01 (d, J = 7.9 Hz, 1H), 7.78 (d, J = 8.1 Hz, 1H), 7.44 (ddd, J = 8.4, 7.2, 1.9 Hz, 1H), 7.04 (d, J = 8.4 Hz, 2H), 6.93–6.88 (m, 3H), 6.72–6.67 (m, 1H), 6.38 (d, J = 8.5 Hz, 1H), 3.04 (s, 3H), 2.51 (t, J = 7.7 Hz, 2H), 1.53 (dtd, J = 9.0, 7.5, 6.2 Hz, 2H), 1.37–1.20 (m, 4H), 0.88 (t, J = 7.2 Hz, 3H). **^{13}C NMR** (150 MHz, $CDCl_3$) δ = 164.5 (C_q), 157.3 (C_q), 147.9 (CH), 142.7 (C_q), 137.5 (CH), 136.7 (C_q), 134.6 (q, $^2J_{C\text{-}F}$ = 32.6 Hz, C_q), 130.9 (C_q), 129.8 (C_q), 129.2 (C_q), 128.9 (CH), 127.5 (CH), 125.8 (q, $^3J_{C\text{-}F}$ = 4.1 Hz, CH), 124.5 (CH), 123.6 (q, $^1J_{C\text{-}F}$ = 273.1 Hz, C_q), 117.2 (q, $^3J_{C\text{-}F}$ = 4.0 Hz, CH), 114.6 (CH), 110.1 (CH), 106.3 (CH), 36.9 (CH_3), 35.6 (CH_2), 31.3 (CH_2), 30.9 (CH_2), 22.5 (CH_2), 14.0 (CH_3). **^{19}F NMR** (565 MHz, $CDCl_3$) δ = -62.48 (s, 3F). **HR-MS** (ESI) *m/z* calcd for $C_{27}H_{27}F_3N_3O$ [M+H$^+$] 466.2101, found 466.2097.

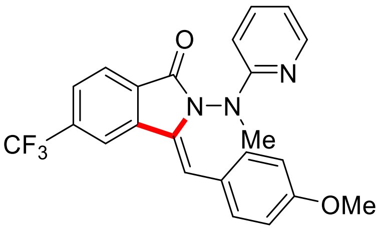

(*Z*)-3-(4-Methoxybenzylidene)-2-(methyl[pyridin-2-yl]amino)-5-(trifluoromethyl)isoindolin-1-one (3cf)

The general procedure was followed using hydrazide **1c** (88.6 mg, 0.30 mmol) and alkyne **2f** (118.9 mg, 0.90 mmol). Purification by column chromatography on silica gel (petroleum/EtOAc 10:1) yielded **3cf** (126.3 mg, 99%, *Z*/*E* = 10:1) as a light yellow solid. mp 122–123 °C. **^{1}H NMR** (600 MHz, $CDCl_3$) δ = 8.19–8.15 (m, 1H), 8.07 (s, 1H), 8.00 (dd, J = 7.9, 0.8 Hz, 1H), 7.78 (d, J = 1.4 Hz, 1H), 7.47 (ddd, J = 8.8, 7.1, 1.8 Hz, 1H), 7.10 (d, J = 9.0 Hz, 2H), 6.88 (s, 1H), 6.74–6.69 (m, 1H), 6.63 (d, J = 8.8 Hz, 2H), 6.45 (d, J = 8.5 Hz, 1H), 3.74 (s, 3H), 3.07 (s, 3H). **^{13}C NMR** (150 MHz, $CDCl_3$) δ = 164.5 (C_q), 159.3 (C_q), 157.5 (C_q), 147.9 (CH), 137.7 (CH), 136.8 (C_q), 134.6 (q, $^2J_{C\text{-}F}$ = 32.8 Hz, C_q), 130.6 (CH), 130.3 (C_q), 129.0 (C_q), 125.7 (q, $^3J_{C\text{-}F}$ = 3.7 Hz, CH), 124.9 (C_q), 124.5 (CH), 123.7 (q, $^1J_{C\text{-}F}$ = 271.5 Hz, C_q), 117.1 (q, $^3J_{C\text{-}F}$ = 4.2 Hz, CH), 114.8 (CH), 113.0 (CH), 110.0 (CH), 106.5 (CH), 55.2 (CH_3), 36.9 (CH_3). **^{19}F NMR**

(565 MHz, $CDCl_3$) δ = -62.51 (s, 3F). **HR-MS** (ESI) *m/z* calcd for $C_{23}H_{19}F_3N_3O_2$ [M+H$^+$] 426.1424, found 426.1421.

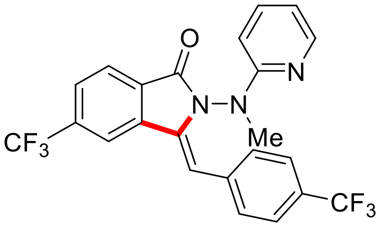

(*Z*)-2-(Methyl[pyridin-2-yl]amino)-5-(trifluoromethyl)-3-(4-(trifluoromethyl)benzylidene)isoindolin-1-one (3cg)

The general procedure was followed using hydrazide **1c** (88.6 mg, 0.30 mmol) and alkyne **2g** (153.1 mg, 0.90 mmol). Purification by column chromatography on silica gel (petroleum/EtOAc 10:1) yielded **3cg** (66.4 mg, 52%) as a light yellow solid. mp 153–154 °C. **^{1}H NMR** (600 MHz, $CDCl_3$) δ = 8.12–8.10 (m, 1H), 8.09 (s, 1H), 8.02 (d, *J* = 7.2 Hz, 1H), 7.84–7.81 (m, 1H), 7.45 (ddd, *J* = 8.8, 7.2, 1.9 Hz, 1H), 7.33 (d, *J* = 8.0 Hz, 2H), 7.22–7.18 (m, 2H), 6.87 (s, 1H), 6.74–6.68 (m, 1H), 6.34 (d, *J* = 8.5 Hz, 1H), 3.02 (s, 3H). **^{13}C NMR** (150 MHz, $CDCl_3$) δ = 164.5 (C_q), 156.9 (C_q), 148.1 (CH), 137.7 (CH), 136.7 (C_q), 136.2 (C_q), 134.9 (q, $^2J_{C\text{-}F}$ = 32.9 Hz, C_q), 132.7 (C_q), 129.5 (C_q), 129.4 (q, $^2J_{C\text{-}F}$ = 32.7 Hz, C_q), 129.0 (CH), 126.5 (q, $^3J_{C\text{-}F}$ = 3.6 Hz, CH), 124.6 (CH), 124.2 (q, $^3J_{C\text{-}F}$ = 3.7 Hz, CH), 123.9 (q, $^1J_{C\text{-}F}$ = 271.7 Hz, C_q), 123.5 (q, $^1J_{C\text{-}F}$ = 273.1 Hz, C_q), 117.4 (q, $^3J_{C\text{-}F}$ = 4.1 Hz, CH), 115.1 (CH), 107.3 (CH), 106.2 (CH), 37.1 (CH_3). **^{19}F NMR** (565 MHz, $CDCl_3$) δ = -62.55 (s, 3F), -62.55 (s, 3F). **HR-MS** (ESI) *m/z* calcd for $C_{23}H_{16}F_6N_3O$ [M+H$^+$] 464.1192, found 464.1189.

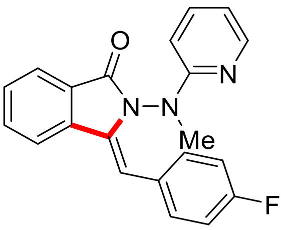

(*Z*)-3-(4-Fluorobenzylidene)-2-(methyl[pyridin-2-yl]amino)isoindolin-1-one (3ah)

The general procedure was followed using hydrazide **1a** (68.2 mg, 0.30 mmol) and

alkyne **2h** (108.1 mg, 0.90 mmol). Purification by column chromatography on silica gel (petroleum/EtOAc 10:1) yielded **3ah** (89.1 mg, 86%, *Z*/*E* = 14:1) as a light yellow solid. mp 101–102 °C. **^{1}H NMR** (600 MHz, $CDCl_3$) δ = 8.14 (ddd, *J* = 5.0, 1.9, 0.9 Hz, 1H), 7.88 (d, *J* = 7.6 Hz, 1H), 7.81 (d, *J* = 7.8 Hz, 1H), 7.69 (dd, *J* = 7.6, 1.1 Hz, 1H), 7.55 (dd, *J* = 7.5, 0.9 Hz, 1H), 7.44 (ddd, *J* = 8.5, 7.1, 1.9 Hz, 1H), 7.08–7.05 (m, 2H), 6.78–6.73 (m, 3H), 6.69 – 6.66 (m, 1H), 6.42 (d, *J* = 8.5 Hz, 1H), 3.02 (s, 3H). **^{13}C NMR** (150 MHz, $CDCl_3$) δ = 165.7 (C_q), 162.0 (d, $^{1}J_{C\text{-}F}$ = 247.0 Hz, C_q), 157.5 (C_q), 147.9 (CH), 137.5 (CH), 136.2 (C_q), 132.9 (CH), 132.3 (C_q), 130.5 (d, $^{3}J_{C\text{-}F}$ = 8.0 Hz, CH), 129.4 (CH), 129.2 (d, $^{4}J_{C\text{-}F}$ = 3.4 Hz, C_q), 126.5 (C_q), 123.8 (CH), 119.8 (CH), 114.5 (CH), 114.3 (d, $^{2}J_{C\text{-}F}$ = 21.5 Hz, CH), 106.5 (CH), 106.4 (CH), 36.7 (CH_3). **^{19}F NMR** (565 MHz, $CDCl_3$) δ = – (113.98-114.03, m, 1F). **HR-MS** (ESI) *m/z* calcd for $C_{21}H_{17}FN_3O$ [M+H$^+$] 346.1350, found 346.1347.

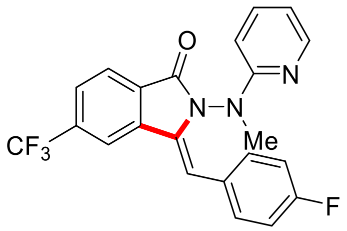

(*Z*)-3-(4-Fluorobenzylidene)-2-(methyl[pyridin-2-yl)amino]-5-(trifluoromethyl)isoindolin-1-one (3ch)

The general procedure was followed using hydrazide **1c** (88.6 mg, 0.30 mmol) and alkyne **2h** (108.1 mg, 0.90 mmol). Purification by column chromatography on silica gel (petroleum/EtOAc 10:1) yielded **3ch** (33.5 mg, 27%) as a light yellow solid. mp 133–134 °C. **^{1}H NMR** (600 MHz, $CDCl_3$) δ = 8.17–8.13 (m, 1H), 8.08 (s, 1H), 8.01 (d, *J* = 7.9 Hz, 1H), 7.80 (d, *J* = 7.9 Hz, 1H), 7.46 (ddd, *J* = 8.7, 7.0, 1.9 Hz, 1H), 7.08 (dd, *J* = 8.5, 5.5 Hz, 2H), 6.86 (s, 1H), 6.78 (d, *J* = 8.6 Hz, 2H), 6.71 (dd, *J* = 7.2, 5.0 Hz, 1H), 6.39 (d, *J* = 8.5 Hz, 1H), 3.03 (s, 3H). ^{13}C NMR (151 MHz, $CDCl_3$) δ = 164.5 (C_q), 162.2 (d, $^{1}J_{C\text{-}F}$ = 248.2 Hz, C_q), 157.2 (C_q), 148.0 (CH), 137.7 (CH), 136.4 (C_q), 134.7 (q, $^{2}J_{C\text{-}F}$ = 32.8 Hz, C_q), 131.6 (C_q), 130.6 (d, $^{3}J_{C\text{-}F}$ = 8.0 Hz, CH), 129.3 (C_q), 128.7 (d, $^{4}J_{C\text{-}F}$ = 3.5 Hz, C_q), 126.1 (q, $^{3}J_{C\text{-}F}$ = 3.6 Hz, CH), 124.6 (CH), 123.6 (q, $^{1}J_{C\text{-}F}$ = 273.2 Hz, C_q), 117.3 (q, $^{3}J_{C\text{-}F}$ = 4.1 Hz, CH), 114.9 (CH), 114.5 (d, $^{2}J_{C\text{-}F}$ = 21.6 Hz, CH), 108.4

(CH), 106.3 (CH), 37.0 (CH_3). **^{19}F NMR** (565 MHz, $CDCl_3$) δ = -62.53 (s, 3F), -113.34 (p, J = 6.2 Hz, 1F). **HR-MS** (ESI) *m/z* calcd for $C_{22}H_{16}F_4N_3O$ [M+H$^+$] 414.1224, found 414.1219.

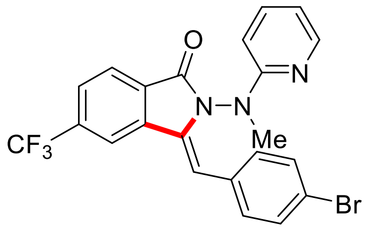

(*Z*)-3-(4-Bromobenzylidene)-2-(methyl[pyridin-2-yl]amino)-5-(trifluoromethyl)isoindolin-1-one (3ci)

The general procedure was followed using hydrazide **1c** (88.6 mg, 0.30 mmol) and alkyne **2i** (162.9 mg, 0.90 mmol). Purification by column chromatography on silica gel (petroleum/EtOAc 10:1) yielded **3ci** (44.1 mg, 31%) as a light yellow solid. mp 170–171 °C. **^{1}H NMR** (600 MHz, $CDCl_3$) δ = 8.16 (ddd, J = 5.0, 1.9, 0.9 Hz, 1H), 8.07 (s, 1H), 8.01 (dd, J = 7.9, 0.8 Hz, 1H), 7.81 (dd, J = 8.0, 0.9 Hz, 1H), 7.51–7.44 (m, 1H), 7.24–7.18 (m, 2H), 6.98 (dd, J = 8.6, 0.8 Hz, 2H), 6.80 (s, 1H), 6.72 (ddd, J = 7.2, 5.0, 0.9 Hz, 1H), 6.39 (dd, J = 8.5, 0.9 Hz, 1H), 3.04 (s, 3H). ^{13}C NMR (151 MHz, $CDCl_3$) δ = 164.5 (C_q), 157.1 (C_q), 148.1 (CH), 137.7 (CH), 136.4 (C_q), 134.8 (q, $^2J_{C\text{-}F}$ = 32.5 Hz, C_q), 131.9 (C_q), 131.7 (C_q), 130.5 (CH), 130.5 (CH), 129.4 (C_q), 126.3 (q, $^3J_{C\text{-}F}$ = 4.1 Hz, CH), 124.6 (CH), 123.6 (q, $^1J_{C\text{-}F}$ = 273.6 Hz, C_q), 121.9 (C_q), 117.3 (q, $^3J_{C\text{-}F}$ = 4.0 Hz, CH), 115.0 (CH), 108.0 (CH), 106.3 (CH), 37.1 (CH_3). **^{19}F NMR** (565 MHz, $CDCl_3$) δ = -62.53 (s, 3F). **HR-MS** (ESI) *m/z* calcd for $C_{22}H_{16}{}^{79}BrF_3N_3O$ [M+H$^+$] 474.0423, found 474.0420.

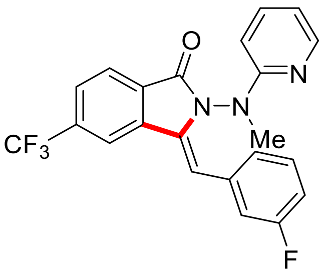

(*Z*)-3-(3-Fluorobenzylidene)-2-(methyl[pyridin-2-yl]amino)-5-(trifluoromethyl)isoindolin-1-one (3cj)

The general procedure was followed using hydrazide **1c** (88.6 mg, 0.30 mmol) and alkyne **2j** (108.1 mg, 0.90 mmol). Purification by column chromatography on silica gel (petroleum/EtOAc 10:1) yielded **3cj** (69.4 mg, 56%) as a light yellow solid. mp 147–148 °C. **^{1}H NMR** (600 MHz, $CDCl_3$) δ = 8.15–8.12 (m, 1H), 8.08 (s, 1H), 8.02 (d, J = 7.9 Hz, 1H), 7.82 (dd, J = 7.9, 1.5 Hz, 1H), 7.46 (ddd, J = 8.8, 7.2, 1.9 Hz, 1H), 7.06 (dd, J = 8.0, 5.9 Hz, 1H), 6.90–6.83 (m, 3H), 6.78 (dd, J = 9.9, 2.2 Hz, 1H), 6.71 (ddd, J = 7.2, 5.0, 0.9 Hz, 1H), 6.37 (d, J = 8.8 Hz, 1H), 3.05 (s, 3H). **^{13}C NMR** (150 MHz, $CDCl_3$) δ = 164.5 (C_q), 161.8 (d, $^1J_{C\text{-}F}$ = 246.3 Hz, C_q), 157.0 (C_q), 148.0 (CH), 137.7 (CH), 136.3 (C_q), 135.0 (d, $^3J_{C\text{-}F}$ = 8.1 Hz, C_q), 134.8 (q, $^2J_{C\text{-}F}$ = 33.1 Hz, C_q), 132.3 (C_q), 129.5 (C_q), 128.9 (d, $^3J_{C\text{-}F}$ = 8.5 Hz, CH), 126.4 (q, $^3J_{C\text{-}F}$ = 3.7 Hz, CH), 124.6 (CH), 124.6 (d, $^4J_{C\text{-}F}$ = 2.9 Hz, CH), 123.6 (q, $^1J_{C\text{-}F}$ = 273.4 Hz, C_q), 117.4 (q, $^3J_{C\text{-}F}$ = 4.0 Hz, CH), 117.3, 115.7 (d, $^2J_{C\text{-}F}$ = 22.3 Hz, CH), 114.9 (CH), 114.6 (d, $^2J_{C\text{-}F}$ = 20.9 Hz, CH), 107.8 (CH), 106.2 (CH), 37.1 (CH_3). **^{19}F NMR** (565 MHz, $CDCl_3$) δ = -62.54 (s, 3F), -113.76 (td, J = 9.3, 6.2 Hz, 1F). **HR-MS** (ESI) *m/z* calcd for $C_{22}H_{16}F_4N_3O$ [M+H$^+$] 414.1224, found 414.1222.

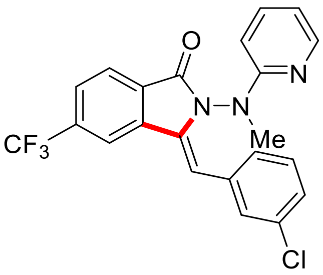

(*Z*)-3-(3-Chlorobenzylidene)-2-(methyl[pyridin-2-yl]amino)-5-(trifluoromethyl)isoindolin-1-one (3ck)

The general procedure was followed using hydrazide **1c** (88.6 mg, 0.30 mmol) and alkyne **2k** (122.9 mg, 0.90 mmol). Purification by column chromatography on silica gel (petroleum/EtOAc 10:1) yielded **3ck** (114.7 mg, 89%) as a light yellow solid. mp 131–132 °C. **^{1}H NMR** (600 MHz, $CDCl_3$) δ = 8.14 (ddd, J = 5.0, 1.8, 0.9 Hz, 1H), 8.07 (s, 1H), 8.02 (d, J = 7.9 Hz, 1H), 7.82 (d, J = 9.3 Hz, 1H), 7.47 (ddd, J = 8.7, 7.1, 1.8 Hz, 1H), 7.16–7.11 (m, 1H), 7.07–7.02 (m, 2H), 7.01–6.98 (m, 1H), 6.82 (s, 1H), 6.71 (dd, J = 7.2, 5.0 Hz, 1H), 6.38 (dd, J = 8.5, 1.0 Hz, 1H), 3.04 (s, 3H). **^{13}C NMR** (150 MHz, $CDCl_3$) δ = 164.5 (C_q), 156.9 (C_q), 148.1 (CH), 137.8 (CH), 136.3 (C_q), 134.8 (q, $^2J_{C\text{-}F}$ = 32.3 Hz, C_q), 134.9 (C_q), 132.4 (C_q), 131.9 (CH), 130.6 (CH), 129.4 (C_q), 128.9 (CH), 127.2 (CH), 126.4 (q, $^3J_{C\text{-}F}$ = 3.8 Hz, CH), 124.6 (CH), 123.6 (q, $^1J_{C\text{-}F}$ = 273.1 Hz, C_q), 121.3 (C_q), 117.4 (q, $^3J_{C\text{-}F}$ = 3.7, 3.2 Hz, CH), 115.1 (CH), 107.4 (CH), 106.2 (CH), 37.0 (CH_3) **^{19}F NMR** (565 MHz, $CDCl_3$) δ = -62.53 (s, 3F). **HR-MS** (ESI) *m/z* calcd for $C_{22}H_{16}{}^{35}ClF_3N_3O$ [M+H$^+$] 430.0929, found 430.0928.

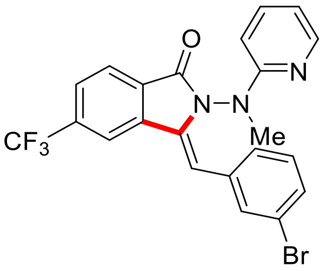

(*Z*)-3-(3-Bromobenzylidene)-2-(methyl(pyridin-2-yl)amino)-5-(trifluoromethyl)isoindolin-1-one (3cl)

The general procedure was followed using hydrazide **1c** (88.6 mg, 0.30 mmol) and alkyne **2l** (162.9 mg, 0.90 mmol). Purification by column chromatography on silica gel (petroleum/EtOAc 10:1) yielded **3cl** (98.2 mg, 69%) as a light yellow solid. mp 135–136 °C. **^{1}H NMR** (600 MHz, $CDCl_3$) δ = 8.17–8.13 (m, 1H), 8.07 (s, 1H), 8.02 (d, J = 7.9 Hz, 1H), 7.82 (d, J = 7.9 Hz, 1H), 7.50–7.45 (m, 1H), 7.28 (dd, J = 7.9, 1.0 Hz, 1H), 7.24 (s, 1H), 7.05 (d, J = 6.8 Hz, 1H), 7.02–6.97 (m, 1H), 6.81 (s, 1H), 6.72 (ddd, J = 7.2, 5.0, 1.0 Hz, 1H), 6.39 (dd, J = 8.4, 1.0 Hz, 1H), 3.04 (s, 3H). **^{13}C NMR** (150 MHz, $CDCl_3$) δ = 164.5 (C_q), 156.9 (C_q), 148.1 (CH), 137.8 (CH), 136.3 (C_q), 134.8 (q, $^2J_{C\text{-}F}$ = 32.3 Hz, C_q), 134.9 (C_q), 132.4 (C_q), 131.9 (CH), 130.6 (CH), 129.4 (C_q), 128.9 (CH), 127.2 (CH), 126.4 (q, $^3J_{C\text{-}F}$ = 3.8 Hz, CH), 124.6 (CH), 123.6 (q, $^1J_{C\text{-}F}$ = 273.1 Hz, C_q), 121.3 (C_q), 117.4 (q, $^3J_{C\text{-}F}$ = 3.7, 3.2 Hz, CH), 115.1 (CH), 107.4 (CH), 106.2 (CH), 37.0 (CH_3). **^{19}F NMR** (565 MHz, $CDCl_3$) δ = -62.53 (s, 3F). **HR-MS** (ESI) *m/z* calcd for $C_{22}H_{16}{}^{79}BrF_3N_3O$ [M+H$^+$] 474.0423, found 474.0424.

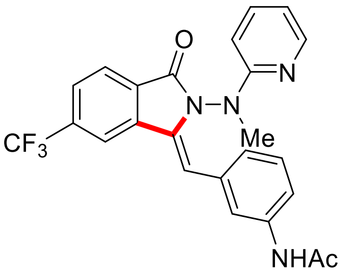

(*Z*)-*N*-[3-{(2-{Methyl[pyridin-2-yl]amino}-3-oxo-6-(trifluoromethyl)isoindolin-1-ylidene)methyl}phenyl]acetamide (3cm)

The general procedure was followed using hydrazide **1c** (88.6 mg, 0.30 mmol) and

alkyne **2m** (143.3 mg, 0.90 mmol). Purification by column chromatography on silica gel (petroleum/EtOAc 10:1) yielded **3cm** (99.1 mg, 73%) as a light yellow solid. mp 75–76 °C. **^{1}H NMR** (600 MHz, $CDCl_3$) δ = 8.14 (dd, J = 5.2, 1.8 Hz, 1H), 8.04 (s, 1H), 7.99 (d, J = 7.9 Hz, 1H), 7.78 (d, J = 8.0 Hz, 1H), 7.48 (ddd, J = 8.7, 7.2, 1.9 Hz, 1H), 7.38–7.33 (m, 1H), 7.14 (s, 1H), 7.10 (s, 1H), 7.04 (dd, J = 7.9, 7.9 Hz, 1H), 7.86 (s, 1H), 6.85 (d, J = 8.7 Hz, 1H), 6.71 (dd, J = 7.1, 5.0 Hz, 1H), 6.42 (d, J = 8.5 Hz, 1H), 2.96 (s, 3H), 2.00 (s, 3H). **^{13}C NMR** (150 MHz, $CDCl_3$) δ = 168.1 (C_q), 164.5 (C_q), 157.3 (C_q), 147.9 (CH), 137.8 (CH), 137.1 (C_q), 136.5 (C_q), 134.8 (q, $^2J_{C\text{-}F}$ = 32.7 Hz, C_q), 133.2 (C_q), 131.4 (C_q), 129.1 (C_q), 128.1 (CH), 126.1 (q, $^3J_{C\text{-}F}$ = 3.8 Hz, CH), 124.5 (CH), 124.4 (CH), 123.5 (q, $^1J_{C\text{-}F}$ = 272.9 Hz, C_q), 120.5 (CH), 119.2 (CH), 117.3 (q, $^3J_{C\text{-}F}$ = 4.1 Hz, CH), 114.5 (CH), 109.2 (CH), 106.5 (CH), 36.8 (CH_3), 24.4 (CH_3). **^{19}F NMR** (565 MHz, $CDCl_3$) δ = -62.51 (s, 3F). **HR-MS** (ESI) *m/z* calcd for $C_{24}H_{20}F_3N_4O_2$ [M+H$^+$] 453.1533, found 453.1533.

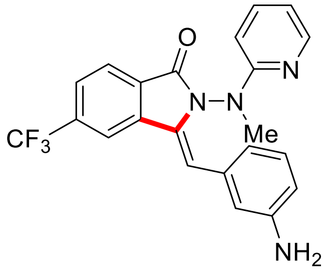

(*Z*)-3-(3-Aminobenzylidene)-2-(methyl[pyridin-2-yl]amino)-5-(trifluoromethyl)isoindolin-1-one (3cn)

The general procedure was followed using hydrazide **1c** (88.6 mg, 0.30 mmol) and alkyne **2n** (105.4 mg, 0.90 mmol). Purification by column chromatography on silica gel (petroleum/EtOAc 10:1) yielded **3cn** (91.1 mg, 74%) as a light yellow solid. mp 115–116 °C. **^{1}H NMR** (600 MHz, $CDCl_3$) δ = 8.19 (d, J = 5.0 Hz, 1H), 8.07 (s, 1H), 8.02 (d, J = 7.9 Hz, 1H), 7.79 (d, J = 7.9 Hz, 1H), 7.49 (ddd, J = 8.8, 7.1, 1.8 Hz, 1H), 6.94 (dd, J = 7.8, 7.8 Hz, 1H), 6.86 (s, 1H), 6.75–6.68 (m, 1H), 6.56 (d, J = 7.5 Hz, 1H), 6.50 (d, J = 8.2 Hz, 1H), 6.45 (d, J = 8.5 Hz, 1H), 6.32 (s, 1H), 3.24 (s_{br}, 2H), 3.01 (s, 3H). **^{13}C NMR** (150 MHz, $CDCl_3$) δ = 164.6 (C_q), 157.4 (C_q), 148.0 (CH), 145.4 (C_q), 137.7 (CH), 136.7 (C_q), 134.7 (q, $^2J_{C\text{-}F}$ = 32.6 Hz, C_q), 133.5 (C_q), 131.0 (C_q), 129.2 (C_q), 128.4 (CH), 126.0 (q, $^3J_{C\text{-}F}$ = 3.6 Hz, CH), 124.5 (CH), 123.6 (q, $^1J_{C\text{-}F}$ = 273.5 Hz, C_q),

119.2 (CH), 117.2 (q, $^3J_{C\text{-}F}$ = 4.2 Hz, CH), 116.0 (CH), 114.7 (CH), 114.4 (CH), 110.1 (CH), 106.3 (CH), 36.8 (CH_3). **^{19}F NMR** (565 MHz, $CDCl_3$) δ = -62.50 (s, 3F). **HR-MS** (ESI) *m/z* calcd for $C_{22}H_{18}F_3N_4O$ [M+H$^+$] 411.1427, found 411.1425.

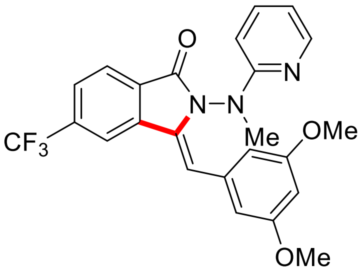

(*Z*)-3-(3,5-Dimethoxybenzylidene)-2-(methyl[pyridin-2-yl]amino)-5-(trifluoromethyl)isoindolin-1-one (3co)

The general procedure was followed using hydrazide **1c** (88.6 mg, 0.30 mmol) and alkyne **2o** (146.0 mg, 0.90 mmol). Purification by column chromatography on silica gel (petroleum/EtOAc 10:1) yielded **3co** (128.4 mg, 94%, *Z*/*E* = 12:1) as a light yellow solid. mp 115–116 °C. **^{1}H NMR** (600 MHz, $CDCl_3$) δ =8.16 (dd, *J* = 5.0, 1.0 Hz, 1H), 8.07 (s, 1H), 8.00 (d, *J* = 7.9 Hz, 1H), 7.79 (d, *J* = 7.5 Hz, 1H), 7.46 (ddd, *J* = 8.8, 7.2, 1.9 Hz, 1H), 6.86 (s, 1H), 6.70 (ddd, *J* = 7.2, 5.0, 0.9 Hz, 1H), 6.45 (d, *J* = 8.5 Hz, 1H), 6.30–6.29 (m, 3H), 3.46 (s, 6H), 3.05 (s, 3H). **^{13}C NMR** (150 MHz, $CDCl_3$) δ = 164.4 (C_q), 159.9 (C_q), 157.4 (C_q), 148.1 (CH), 137.7 (CH), 136.5 (C_q), 134.7 (C_q), 134.7 (q, $^3J_{C\text{-}F}$ = 32.4 Hz, C_q), 131.5 (C_q), 129.3 (C_q), 126.1 (q, $^3J_{C\text{-}F}$ = 3.7 Hz, CH), 124.5 (CH), 123.6 (q, $^1J_{C\text{-}F}$ = 273.4 Hz, C_q), 117.3 (q, $^3J_{C\text{-}F}$ = 4.3 Hz, CH), 114.8 (CH), 109.5 (CH), 106.7 (CH), 106.5 (CH), 100.6 (CH), 55.0 (CH_3), 37.0 (CH_3). **^{19}F NMR** (565 MHz, $CDCl_3$) δ = -62.51 (s, 3F). **HR-MS** (ESI) *m/z* calcd for $C_{24}H_{21}F_3N_3O_3$ [M+H$^+$] 456.1530, found 456.1529.

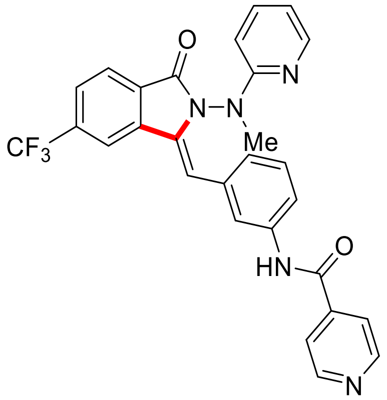

(*Z*)-*N*-{3-({2-[Methyl(pyridin-2-yl)amino]-3-oxo-6-(trifluoromethyl)isoindolin-1-ylidene}methyl)phenyl}isonicotinamide (3cp)

The general procedure was followed using hydrazide **1c** (88.6 mg, 0.30 mmol) and alkyne **2p** (200.0 mg, 0.90 mmol). Purification by column chromatography on silica gel (petroleum/EtOAc 10:1) yielded **3cp** (136.1 mg, 88%) as a light yellow solid. mp 70–71 °C. **^{1}H NMR** (600 MHz, $CDCl_3$) δ = 8.77–8.71 (m, 2H), 8.12–8.05 (m, 2H), 7.99 (d, J = 7.2 Hz, 1H), 7.79 (d, J = 8.0 Hz, 1H), 7.70 (s, 1H), 7.59–7.55 (m, 2H), 7.52 (d, J = 8.1 Hz, 1H), 7.42 (ddd, J = 8.7, 7.1, 1.9 Hz, 1H), 7.27 (s, 1H), 7.15 (t, J = 7.9 Hz, 1H), 7.01 – 6.95 (m, 1H), 6.89 (s, 1H), 6.57 (ddd, J = 7.2, 5.0, 1.0 Hz, 1H), 6.43 (d, J = 8.5 Hz, 1H), 2.97 (s, 3H). **^{13}C NMR** (150 MHz, $CDCl_3$) δ = 164.4 (C_q), 163.4 (C_q), 157.3 (C_q), 150.6 (CH), 147.9 (CH), 141.7 (C_q), 137.8 (CH), 136.5 (C_q), 136.4 (C_q), 134.9 (d, $^{2}J_{C\text{-}F}$ = 32.5 Hz, C_q), 133.5 (C_q), 131.7 (C_q), 129.1 (C_q), 128.4 (CH), 126.3 (q, $^{3}J_{C\text{-}F}$ = 3.7 Hz, CH), 125.4 (CH), 124.6 (CH), 123.5 (d, $^{1}J_{C\text{-}F}$ = 273.2 Hz, C_q), 121.2 (CH), 120.8 (CH), 119.9 (CH), 117.3 (q, $^{3}J_{C\text{-}F}$ = 3.9 Hz, CH), 114.6 (CH), 108.8 (CH), 106.5 (CH), 36.8 (CH_3). **^{19}F NMR** (565 MHz, $CDCl_3$) δ = -62.53 (s, 3F). **HR-MS** (ESI) *m/z* calcd for $C_{28}H_{21}F_3N_5O_2$ [M+H$^+$] 516.1642, found 516.1641.

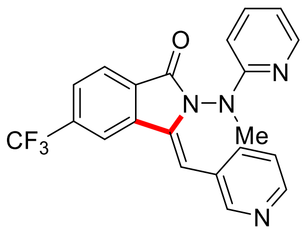

(*Z*)-2-(Methyl[pyridin-2-yl]amino)-3-(pyridin-3-ylmethylene)-5-(trifluoromethyl)isoindolin-1-one (3cq)

The general procedure was followed using hydrazide **1c** (88.6 mg, 0.30 mmol) and alkyne **2q** (92.8 mg, 0.90 mmol). Purification by column chromatography on silica gel (petroleum/EtOAc 10:1) yielded **3cq** (105.8 mg, 89%) as a light yellow solid. mp 141–142 °C. **^{1}H NMR** (600 MHz, $CDCl_3$) δ = 8.42 (dd, J = 2.2, 1.0 Hz, 1H), 8.37 (ddd, J = 4.9, 1.7, 0.7 Hz, 1H), 8.12 (ddd, J = 4.9, 1.9, 0.9 Hz, 1H), 8.10 (s, 1H), 8.01 (d, J = 7.9 Hz, 1H), 7.82 (d, J = 7.9 Hz, 1H), 7.45 (ddd, J = 8.4, 7.2, 1.9 Hz, 1H), 7.38 (ddd, J = 7.8, 2.4, 1.7, 1H), 6.99 (dd, J = 7.8, 4.9 Hz, 1H), 6.80 (s, 1H), 6.73–6.68 (m, 1H), 6.36 (d, J = 8.5 Hz, 1H), 3.07 (s, 3H). **^{13}C NMR** (150 MHz, $CDCl_3$) δ = 164.4 (C_q), 156.9 (C_q), 149.2 (CH), 148.6 (CH), 148.1 (CH), 137.8 (CH), 136.0 (C_q), 135.9 (CH), 134.9 (q, $^{2}J_{C\text{-}F}$ = 32.8 Hz, C_q), 133.2 (C_q), 129.5 (C_q), 129.1 (C_q), 126.5 (q, $^{3}J_{C\text{-}F}$ = 3.8 Hz, CH), 124.6 (CH), 123.5 (d, $^{1}J_{C\text{-}F}$ = 273.1 Hz, C_q) 122.1 (CH), 117.5 (q, $^{3}J_{C\text{-}F}$ = 4.1 Hz, CH), 115.3 (CH), 106.3 (CH), 104.9 (CH), 37.2 (CH_3). **^{19}F NMR** (565 MHz, $CDCl_3$) δ = -62.55 (s, 3F). **HR-MS** (ESI) *m/z* calcd for $C_{21}H_{16}F_3N_4O$ [M+H^{+}] 397.1271, found 397.1269.

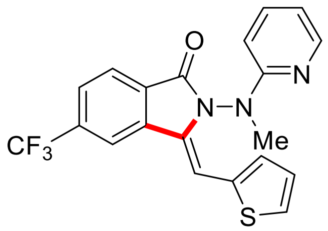

(*Z*)-2-(Methyl[pyridin-2-yl]amino)-3-(thiophen-2-ylmethylene)-5-(trifluoromethyl)isoindolin-1-one (3cr)

The general procedure was followed using hydrazide **1c** (88.6 mg, 0.30 mmol) and alkyne **2r** (105 mg, 0.90 mmol). Purification by column chromatography on silica gel (petroleum/EtOAc 10:1) yielded **3cr** (97.3 mg, 88%) as a light yellow solid. mp 115–

116 °C. **^{1}H NMR** (600 MHz, $CDCl_3$) δ = 8.23 (ddd, J = 5.0, 1.9, 0.9 Hz, 1H), 8.07 (s, 1H), 8.00 (d, J = 7.9 Hz, 1H), 7.78 (d, J = 7.9 Hz, 1H), 7.51–7.43 (m, 1H), 7.18–7.17 (m, 1H), 7.15 (ddd, J = 4.1, 3.0, 1.1 Hz, 1H), 6.98 (dd, J = 5.0, 1.3 Hz, 1H), 6.80 (s, 1H), 6.75 (ddd, J = 7.2, 5.0, 0.9 Hz, 1H), 6.49 (d, J = 8.5 Hz, 1H), 3.24 (s, 3H). **^{13}C NMR** (150 MHz, $CDCl_3$) δ = 164.3 (C_q), 157.9 (C_q), 148.1 (CH), 137.9 (CH), 136.8 (C_q), 134.6 (q, $^{2}J_{C\text{-}F}$ = 32.7 Hz, C_q), 132.8 (C_q), 130.4 (C_q), 129.3 (CH), 128.9 (C_q), 125.8 (q, $^{3}J_{C\text{-}F}$ = 3.6 Hz, CH), 125.6 (CH), 125.0 (CH), 124.5 (CH), 123.6 (d, $^{1}J_{C\text{-}F}$ = 271.3 Hz, C_q), 117.0 (q, $^{3}J_{C\text{-}F}$ = 3.9 Hz, CH), 115.2 (CH), 106.7 (CH), 104.2 (CH), 37.3 (CH_3). **^{19}F NMR** (565 MHz, $CDCl_3$) δ = -62.49 (s, 3F). **HR-MS** (ESI) *m/z* calcd for $C_{20}H_{15}F_3N_3OS$ [M+H$^+$] 402.0882, found 402.0880.

Copper-mediated decarboxylative C−H/N−H activation

(a) Copper-mediated decarboxylative C-H/N-H annulaiton

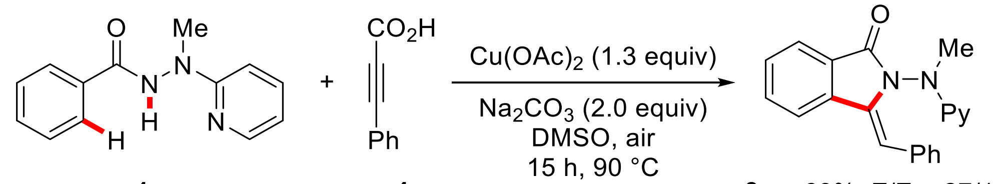

To a 25-mL Schlenk tube were added benzhydrazide **1** (68.2 mg, 0.30 mmol, 1.00 equiv), 3-phenylpropiolic acid (131.5 mg, 0.90 mmol, 3.0 equiv), $Cu(OAc)_2$ (71 mg, 0.39 mmol, 1.30 equiv), and Na_2CO_3 (64 mg, 0.60 mmol, 2.00 equiv) under an air atmosphere. The mixture was stirred at 90 °C for 15 h. At ambient temperature, H_2O (15 mL) and Et_3N (0.5 mL) were added and a suspension was formed immediately. After being filtered through a Celite® pad, the reaction mixture was extracted with EtOAc (3×20 mL). The combined organic phase was washed with brine (20 mL) and dried over Na_2SO_4. Then Et_3N (0.5 mL) and silica gel (0.8 g) were added and the combined solvent was removed under reduced pressure. The residue solid sample was purified by column chromatography on silica gel (petroleum/EtOAc = 6:1, with 1% Et_3N) and yielded the desired product **3aa** (61.9 mg, 63%, *Z*/*E* = 27:1).

Removal of the directing group

(b) Removal of the Directing Group

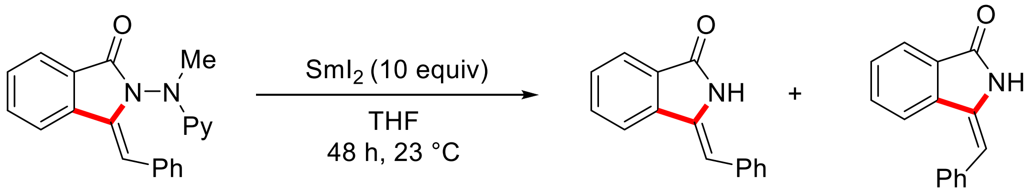

3aa (Z/E = 13/1) (Z)-**S-3aa:** 63% (E)-**S-3aa:** 16%

An oven-dried 100-mL Schlenk round bottom flask was charged with **3aa** (0.22 mmol, 73 mg). After purging with argon three times, freshly distilled THF (5.0 mL) was added, followed by SmI_2 (0.1 M in THF, 22 mL, 10 equiv), which was added dropwise at 0 °C. 30 minutes later, the mixture was warmed to ambient temperature and stirred for an additional 48 h. Then, the mixture was quenched with saturated aqueous $Na_2S_2O_3$ (5.0 mL) and extracted with DCM (3×20 mL), dried over Na_2SO_4, filtered, and concentrated under reduced pressure. The residue was purified by column chromatography on silica gel (petroleum/EtOAc = 5:1) and yielded the desired products (*Z*)-**S-3aa** (31.1 mg, 63%) and (*E*)-**S-3aa** (8.0 mg, 16%). **(*Z*)-3-Benzylideneisoindolin-1-one: ^{1}H NMR** (600 MHz, $CDCl_3$) δ = 8.40 (s_{br}, 1H), 7.87 (d, *J* = 7.6 Hz, 1H), 7.78 (d, *J* = 7.7 Hz, 1H), 7.63 (dd, *J* = 7.6, 1.1 Hz, 1H), 7.51 (dd, *J* = 7.5, 0.9 Hz, 1H), 7.48–7.40 (m, 4H), 7.30 (dd, *J* = 7.0, 1.5 Hz, 1H), 6.55 (s, 1H). **^{13}C NMR** (150 MHz, $CDCl_3$) δ = 169.0 (C_q), 138.2 (C_q), 135.0 (C_q), 133.1 (C_q), 132.2 (CH), 129.2 (CH), 129.2 (CH), 128.7 (C_q), 128.5 (CH), 127.7 (CH), 123.5 (CH), 119.8 (CH), 105.9 (CH). **(*E*)-3-Benzylideneisoindolin-1-one: ^{1}H NMR** (600 MHz, $CDCl_3$) δ = 8.80 (s, 1H), 7.87 (dd, *J* = 7.6, 1.1 Hz, 1H), 7.50–7.40 (m, 6H), 7.39–7.34 (m, 2H), 6.66 (s, 1H). **^{13}C NMR** (150 MHz, $CDCl_3$) δ = 168.3 (C_q), 135.5 (C_q), 134.9 (C_q), 134.5 (C_q), 131.8 (CH), 131.3 (C_q), 129.5 (CH), 129.4 (CH), 128.6 (CH), 127.9 (CH), 123.4 (CH), 123.4 (CH), 112.1 (CH). The analytical data are in accordance with those previously reported in the literature [3-5].

Mechanistic studies

Competition experiments

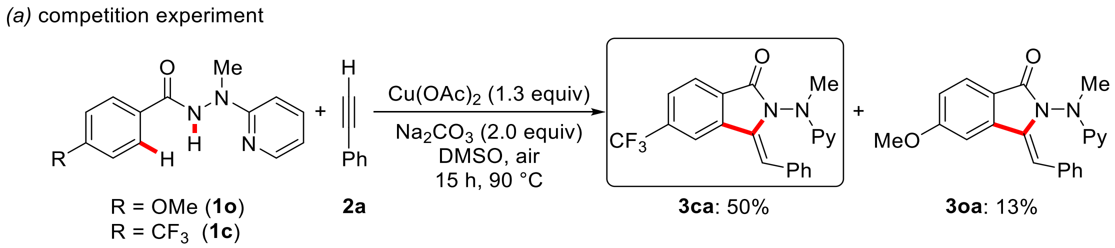

The general procedure was followed using hydrazides **1o** (77.2 mg, 0.30 mmol) and **1c** (88.6 mg, 0.30 mmol) and ethynylbenzene (**2a**, 91.8 mg, 0.9 mmol). The mixture was stirred at 90 °C for 3 h. At ambient temperature, H_2O (15 mL) and Et_3N (0.5 mL) were added, and a suspension was formed immediately. After being filtered through a Celite® pad, the reaction mixture was extracted with EtOAc/NEt_3 (100:1, 3×20 mL). The combined organic phase was washed with brine (20 mL) and dried over Na_2SO_4. After evaporation of the solvent, the crude mixture was analyzed by ^{1}H NMR using 1,3,5-trimethoxybenzene (9.5 mg, 0.056 mmol) as the internal standard, which showed a product distribution of 3.85:1 in favor of **3ca**.

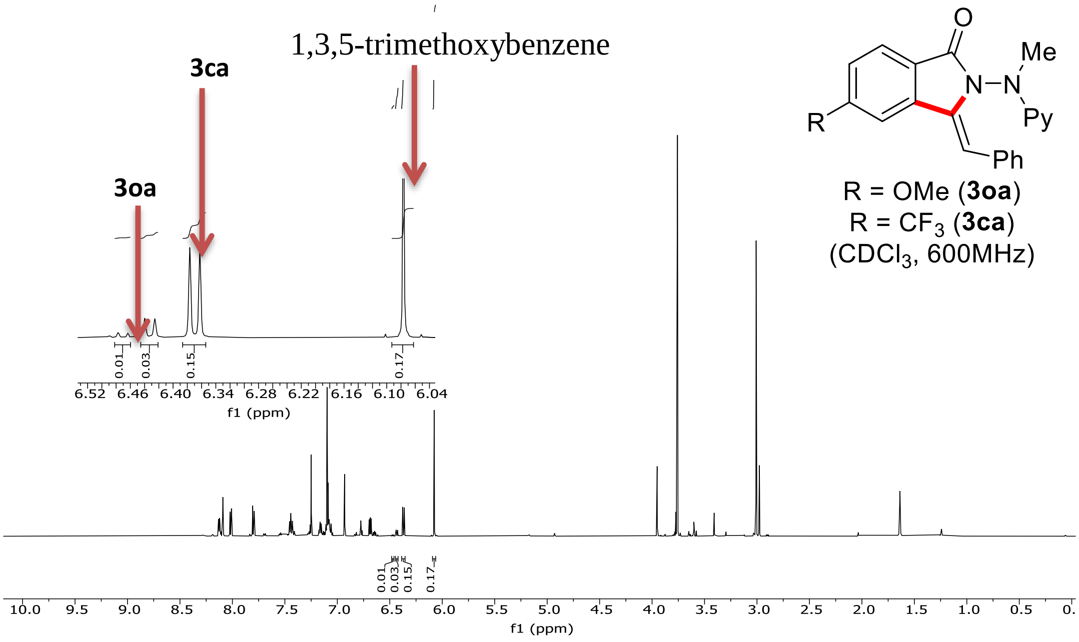

Figure S-1: ^{1}H NMR spectrum of a mixture of **3ca** and **3oa**.

(b) competition experiment

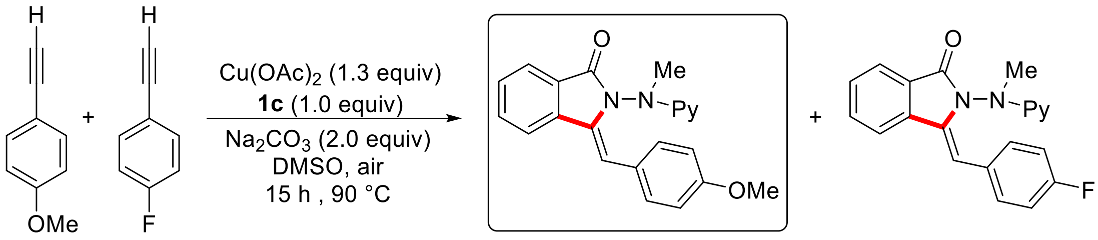

2f **2h** **3cf**: 37% **3ch**: 13%

The general procedure was followed using hydrazide **1c** (88.6 mg, 0.30 mmol), alkyne **2f** (59.5 mg, 0.45 mmol), and alkyne **2i** (54.1 mg, 0.45 mmol). The mixture was stirred at 90 °C for 3 h. At ambient temperature, H_2O (15 mL) and Et_3N (0.5 mL) were added, and a suspension was formed immediately. After being filtered through a Celite® pad, the reaction mixture was extracted with EtOAc/NEt_3 (100:1, 3×20 mL). The combined organic phase was washed with brine (20 mL) and dried over Na_2SO_4. After evaporation of the solvent, the crude mixture was analyzed by ^{1}H NMR using 1,3,5-trimethoxybenzene (9.3 mg, 0.055 mmol) as the internal standard, which showed a product distribution of 2.85:1 in favor of **3cf**.

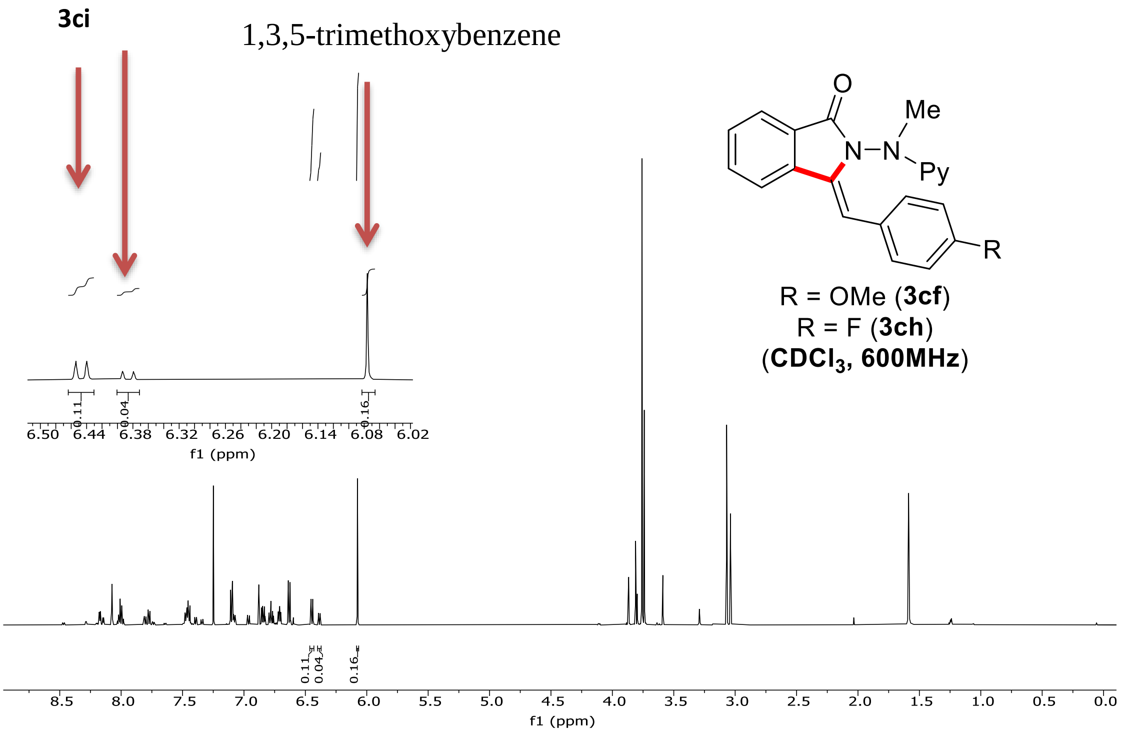

Figure S-2: ^{1}H NMR spectrum of a mixture of **3cf** and **3ch**.

Deuteration experiment

(c) H/D exchange experiment

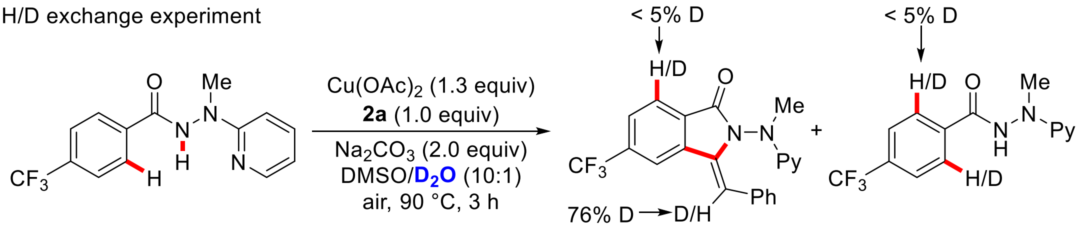

1c (3.0 equiv) [D]$_n$-**3ca**: 36% [D]$_n$-**1c**: 50%

The general procedure was followed using alkyne **2a** (30.6 mg, 0.30 mmol) and hydrazide **1c** (265.8 mg, 0.90 mmol). The mixture was stirred in a solvent mixture of DMSO/D_2O (10:1, 6.6 mL) at 90 °C for 3 h. At ambient temperature, H_2O (15 mL) and Et_3N (0.5 mL) were added, and a suspension was formed immediately. After being filtered through a Celite® pad, the reaction mixture was extracted with EtOAc/NEt_3 (100:1, 3×20 mL). The combined organic phase was washed with brine (20 mL) and dried over Na_2SO_4. Then Et_3N (1.0 mL), silica gel (0.8 g) were added and the combined solvent was removed under reduced pressure. The residue solid sample was purified by column chromatography on silica gel (petroleum/EtOAc = 5:1 to 2:1, with 1% Et_3N) and yielded the desired product [D]$_n$-**3ca** (43 mg, 36%) and reisolated starting material [D]$_n$-**1c** (132 mg, 50%) as white solids. The H/D-scrambling was analyzed in each of the compounds by ^{1}H NMR spectroscopy.

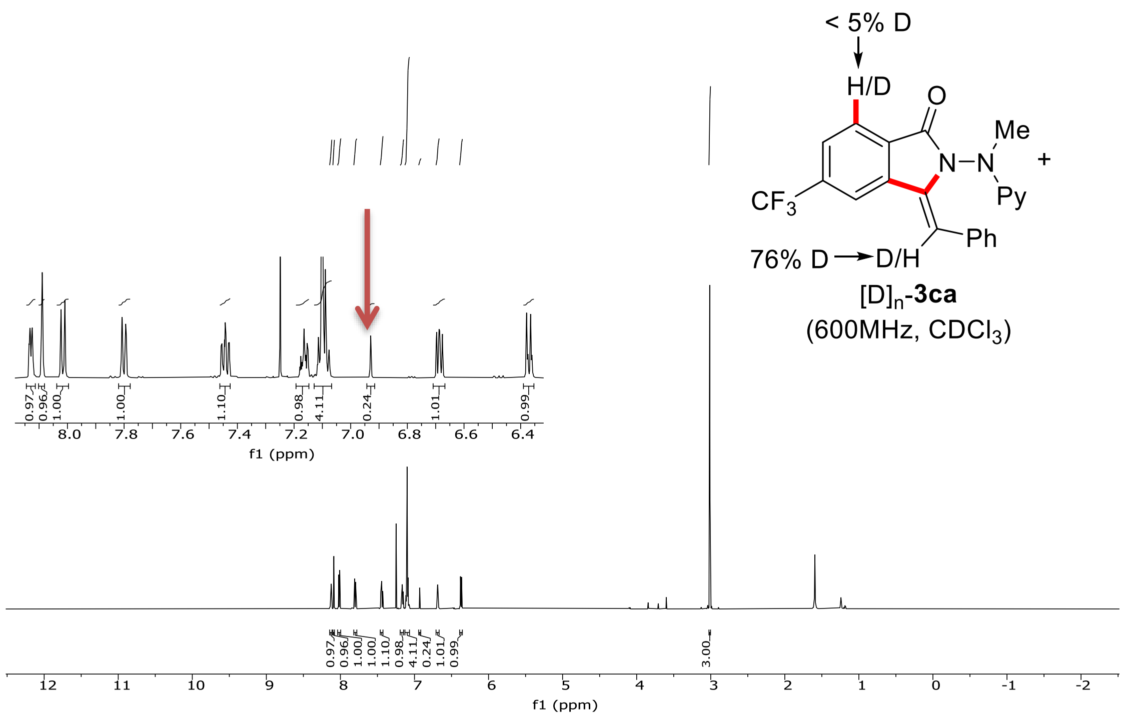

Figure S-3: ^{1}H NMR spectrum of a mixture of [D]$_n$-**3ca**.

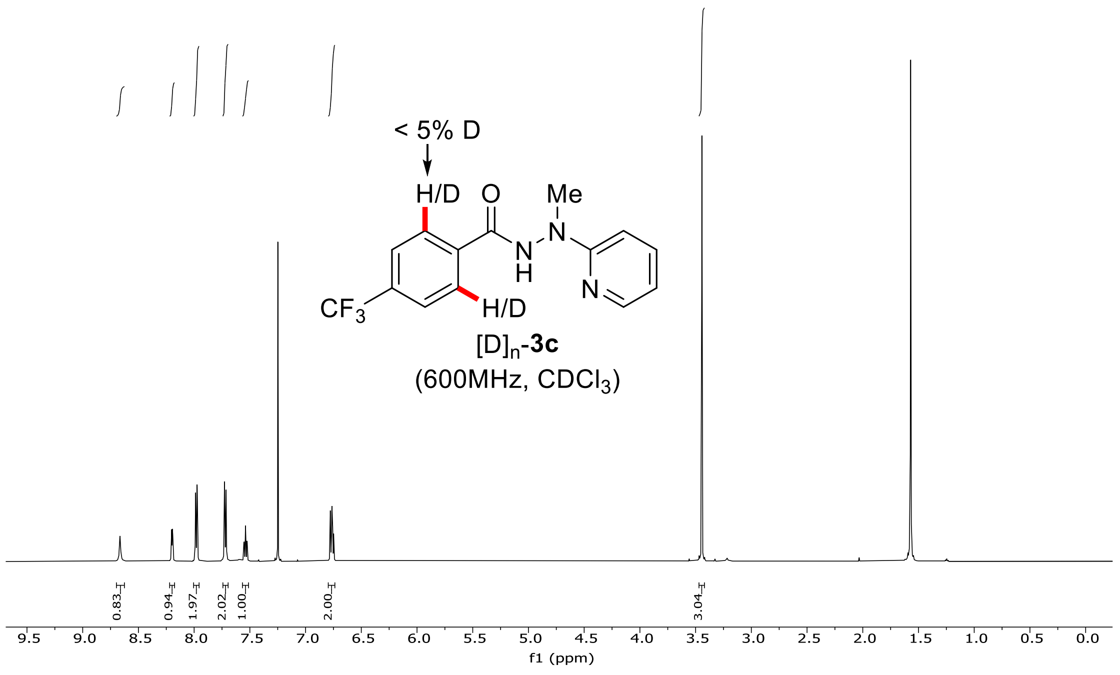

Figure S-4: ^{1}H NMR spectrum of a mixture of [D]$_n$-**1c**.

KIE studies

Parallel experiment

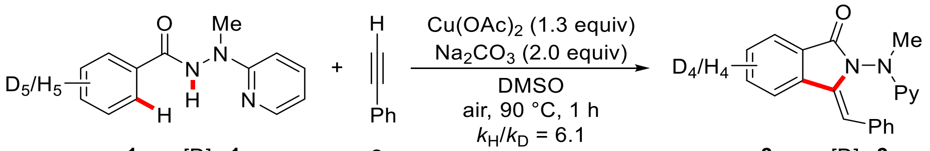

Two independent reactions following the general procedure were carried out using substrates **1a**, [D]$_5$-**1a** (0.30 mmol each), and ethynylbenzene (**2a**, 91.8 mg, 0.90 mmol). The mixture was stirred at 90 °C for 1 h. At ambient temperature, these two mixtures were combined and quenched by adding H_2O (15 mL) and Et_3N (0.5 mL). After being filtered through a Celite® pad, the reaction mixture was extracted with EtOAc/Et_3N (100:1, 3×20 mL). The combined organic phase was washed with brine (20 mL) and dried over Na_2SO_4. Then, Et_3N (1.0 mL) and silica gel (0.8 g) were added, and the combined solvent was removed under reduced pressure. The residual solid sample was purified by column chromatography on silica gel (petroleum/EtOAc = 5:1 to 2:1, with 1% Et_3N) and yielded the mixture of the desired products [D]$_4$-**3aa** and **3aa** (53.0 mg, 27%). The H/D-scrambling was analyzed by ^{1}H NMR spectroscopy.

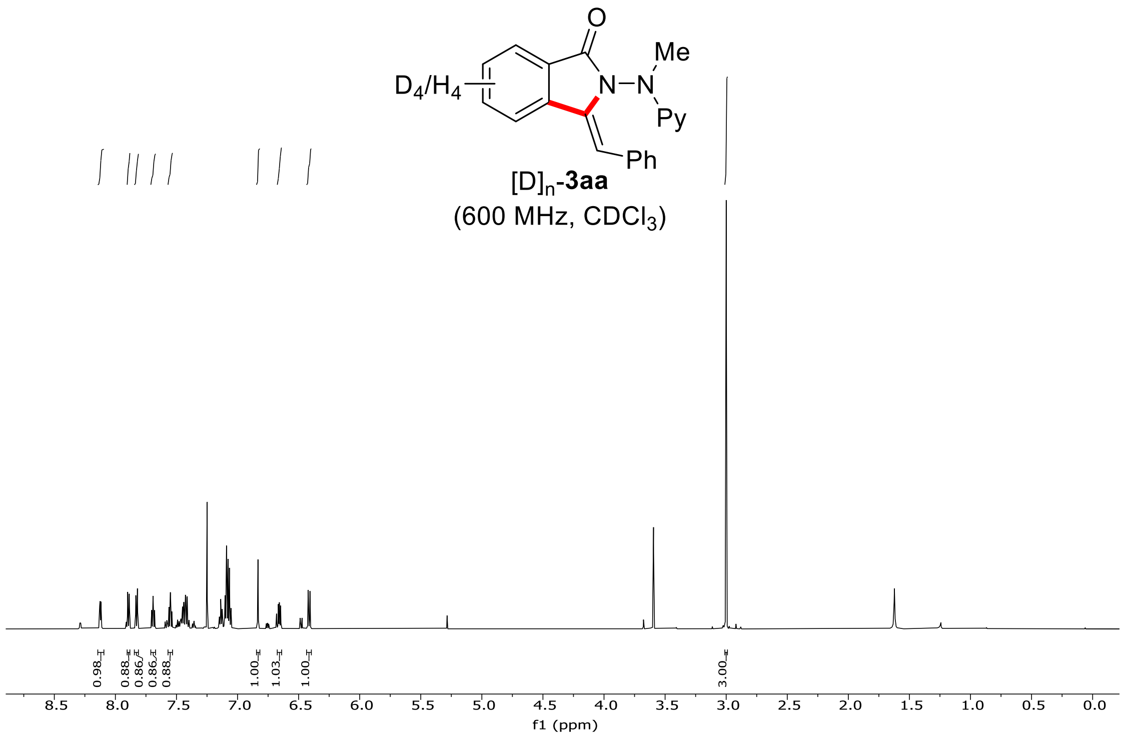

Figure S-5: ^{1}H NMR spectrum of **3aa** and [D]$_4$-**3aa** for the parallel experiment.

competition experiment

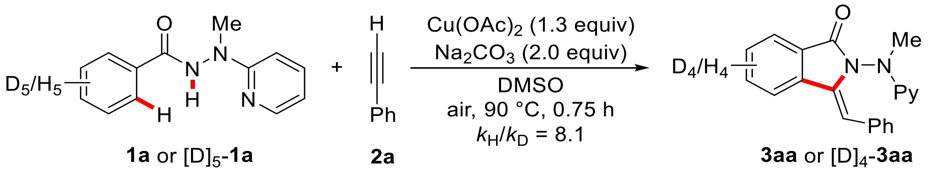

The general procedure was followed using hydrazide **1a** (68.2 mg, 0.30 mmol), [D]$_5$-**1a** (69.4 mg, 0.30 mmol), and ethynylbenzene (**2a**, 91.8 mg, 0.90 mmol). The mixture was stirred at 90 °C for 0.75 h. At ambient temperature, H_2O (15 mL) and Et_3N (0.5 mL) were added, and a suspension was formed immediately. After being filtered through a Celite® pad, the reaction mixture was extracted with EtOAc/NEt_3 (100:1, 3×20 mL). The combined organic phase was washed with brine (20 mL) and dried over Na_2SO_4. Then, Et_3N (1.0 mL) and silica gel (0.8 g) were added, and the combined solvent was removed under reduced pressure. The residual solid sample was purified by column chromatography on silica gel (petroleum/EtOAc = 5:1 to 2:1, with 1% Et_3N)

and yielded the mixture of the desired products [D]$_4$-**3aa** and **3aa** (37.3 mg, 19%) as a light yellow oil. The H/D-scrambling was analyzed by ^{1}H NMR spectroscopy.

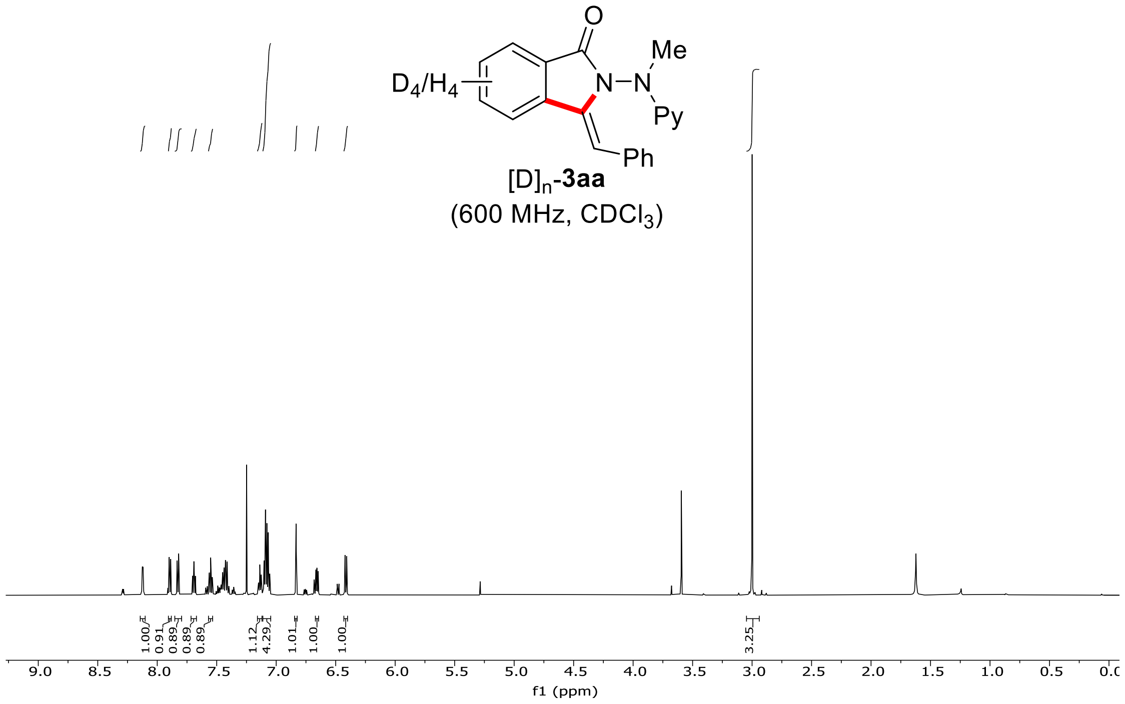

Figure S-6: ^{1}H NMR spectrum of **3aa** and [D]$_4$-**3aa** for the competition experiment.

References

[1] Zhai, S.; Qiu, S.; Chen, X.; Wu, J.; Zhao, H.; Tao, C.; Li, Y.; Cheng, B.; Wang, H.; Zhai, H. *Chem. Commun.* **2018**, *54*, 98–101.

[2] Mei, R.; Sauermann, N.; Oliveira, J. C.; Ackermann, L. *J. Am. Chem. Soc.* **2018**, *140*, 7913–7921.

[3] a) Munoz, S. B.; Krishnamurti, V.; Barrio, P.; Mathew, T.; Prakash, G. K. S. *Org. Lett.* **2018**, *20*, 1042–1045.

[4] Zhang, L.-B.; Hao, X.-Q.; Liu, Z.-J.; Zheng, X.-X.; Zhang, S.-K.; Niu, J.-L.; Song, M.-P. *Angew. Chem., Int. Ed.* **2015**, *54*, 10012–10015.

[5] Li, L.; Wang, M.; Zhang, X.; Jiang, Y.; Ma, D. *Org. Lett.* **2009**, *11*, 1309–1312.

NMR spectra

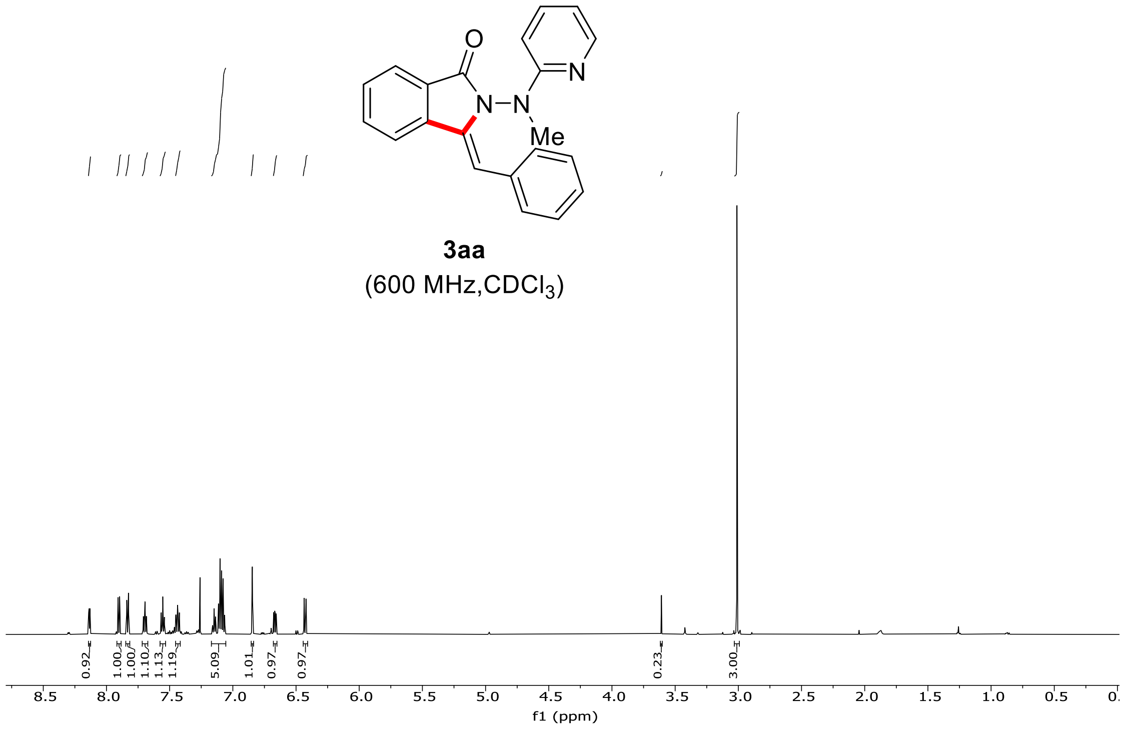

O
N
N–N
Me
3aa
(600 MHz,CDCl3)
0.92
1.00
1.00
1.10
1.13
1.19
5.09
1.01
0.97
0.97
0.23
3.00
8.5 8.0 7.5 7.0 6.5 6.0 5.5 5.0 4.5 4.0 3.5 3.0 2.5 2.0 1.5 1.0 0.5
f1 (ppm)

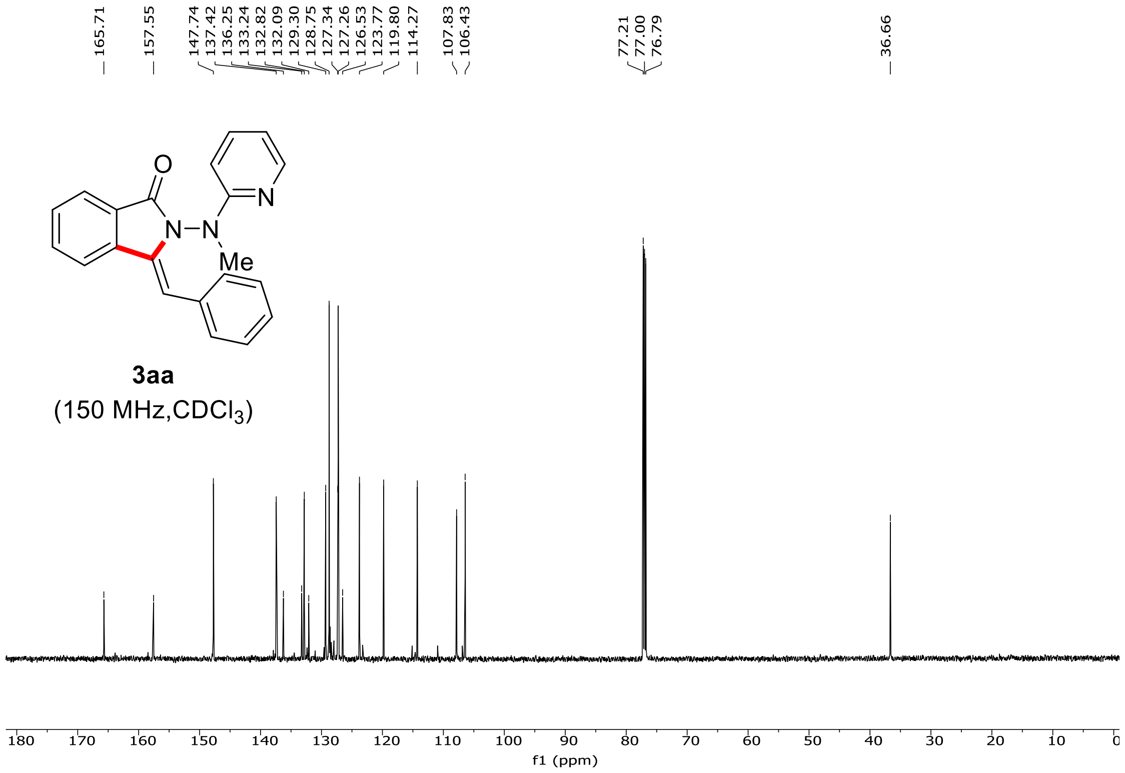

165.71
157.55
147.74
137.42
136.25
133.24
132.82
132.09
129.30
128.75
127.34
127.26
126.53
123.77
119.80
114.27
107.83
106.43
77.21
77.00
76.79
36.66
O
N
N–N
Me
3aa
(150 MHz,CDCl3)
180 170 160 150 140 130 120 110 100 90 80 70 60 50 40 30 20 10
f1 (ppm)

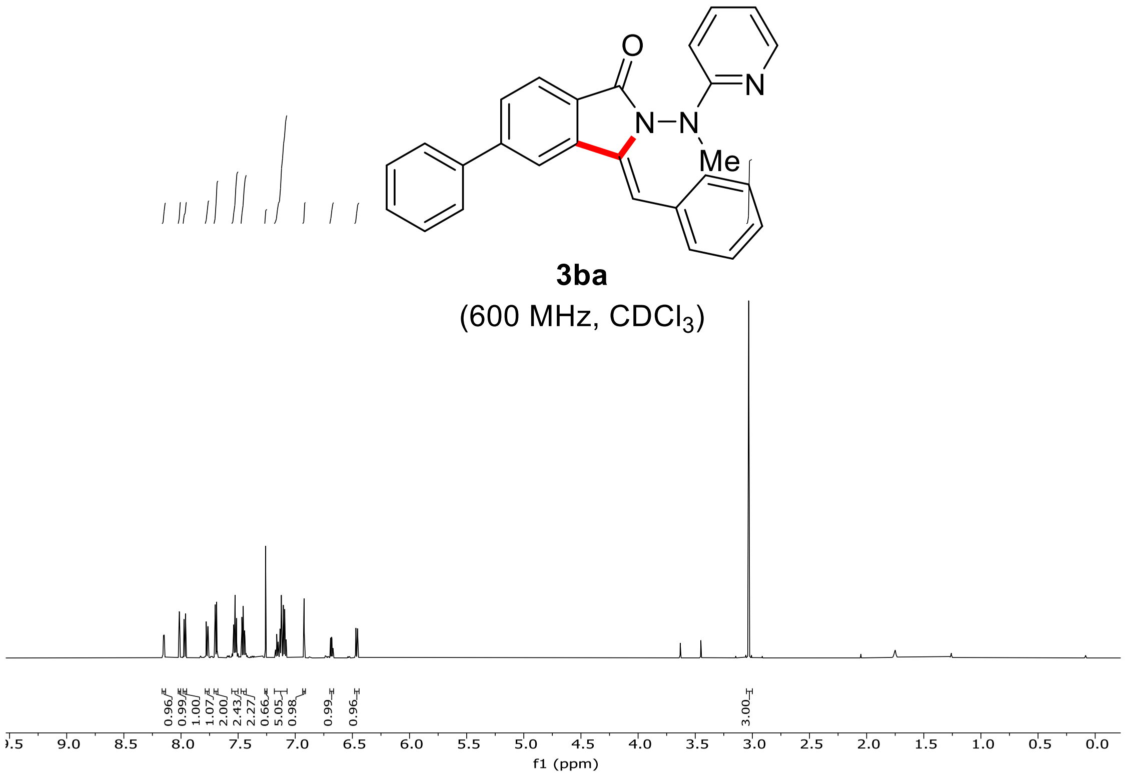

3ba

(600 MHz, $CDCl_3$)

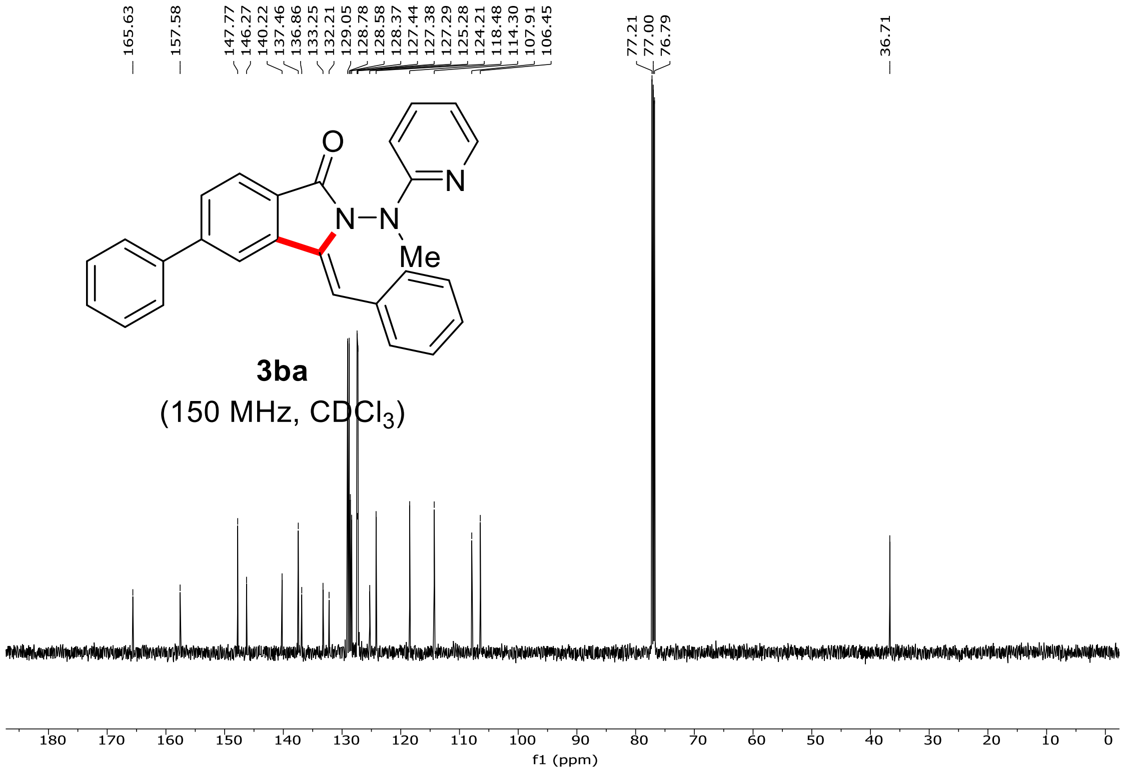

3ba

(150 MHz, $CDCl_3$)

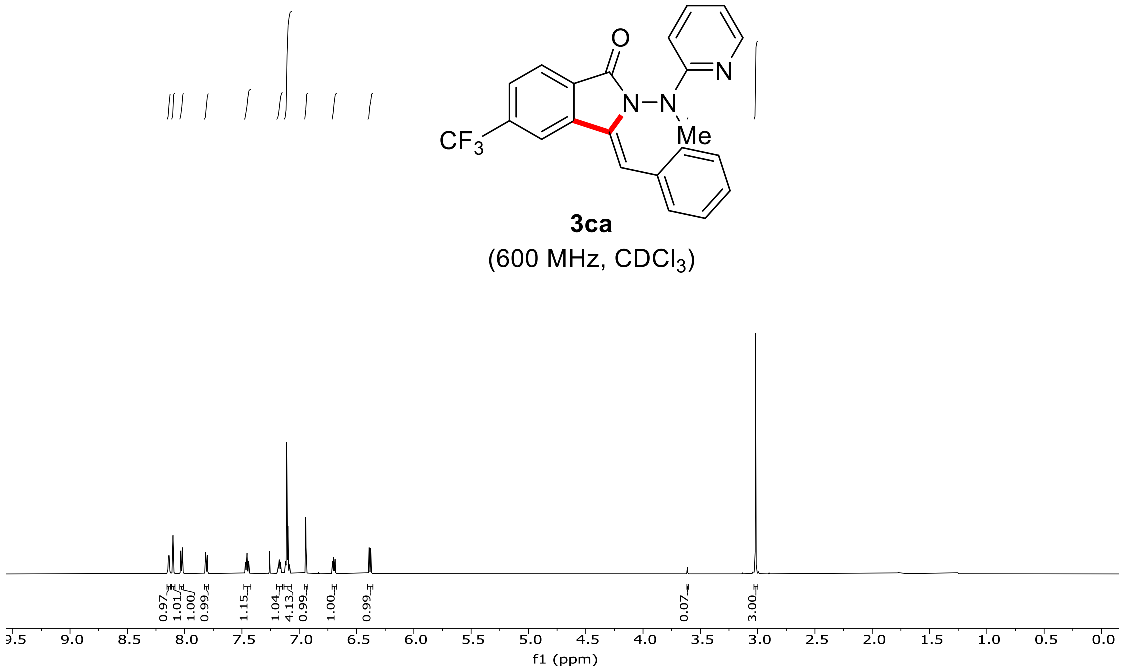

3ca
(600 MHz, $CDCl_3$)

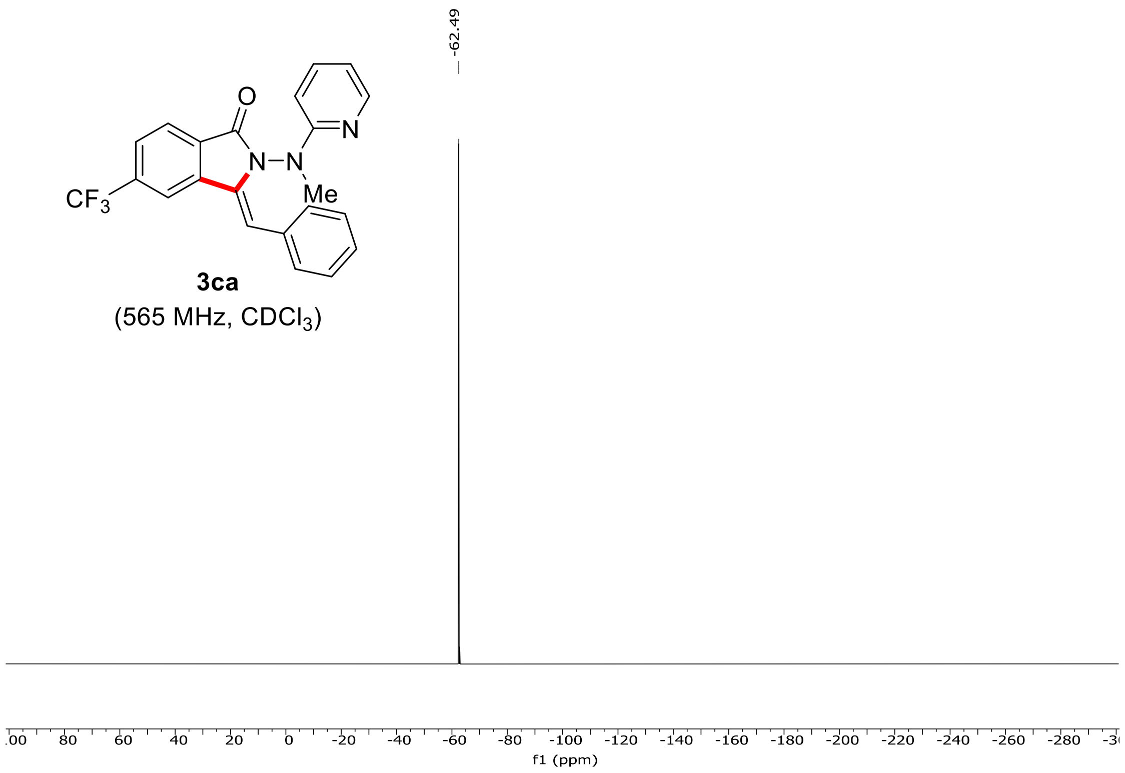

3ca
(565 MHz, $CDCl_3$)

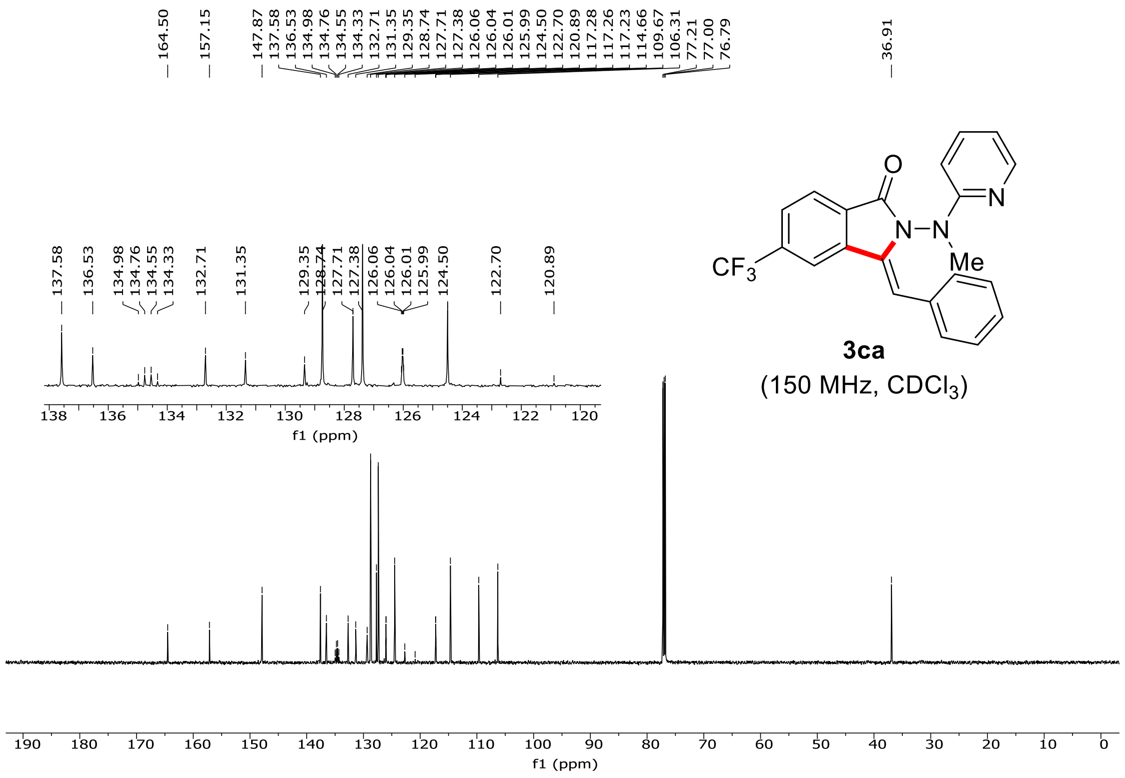
164.50
157.15
147.87
137.58
136.53
134.98
134.76
134.55
134.33
132.71
131.35
129.35
128.74
127.71
127.38
126.06
126.04
126.01
125.99
124.50
122.70
120.89
117.28
117.26
117.23
114.66
109.67
106.31
77.21
77.00
76.79
36.91
O
N
N–N
Me
CF_3
3ca
(150 MHz, $CDCl_3$)
f1 (ppm)

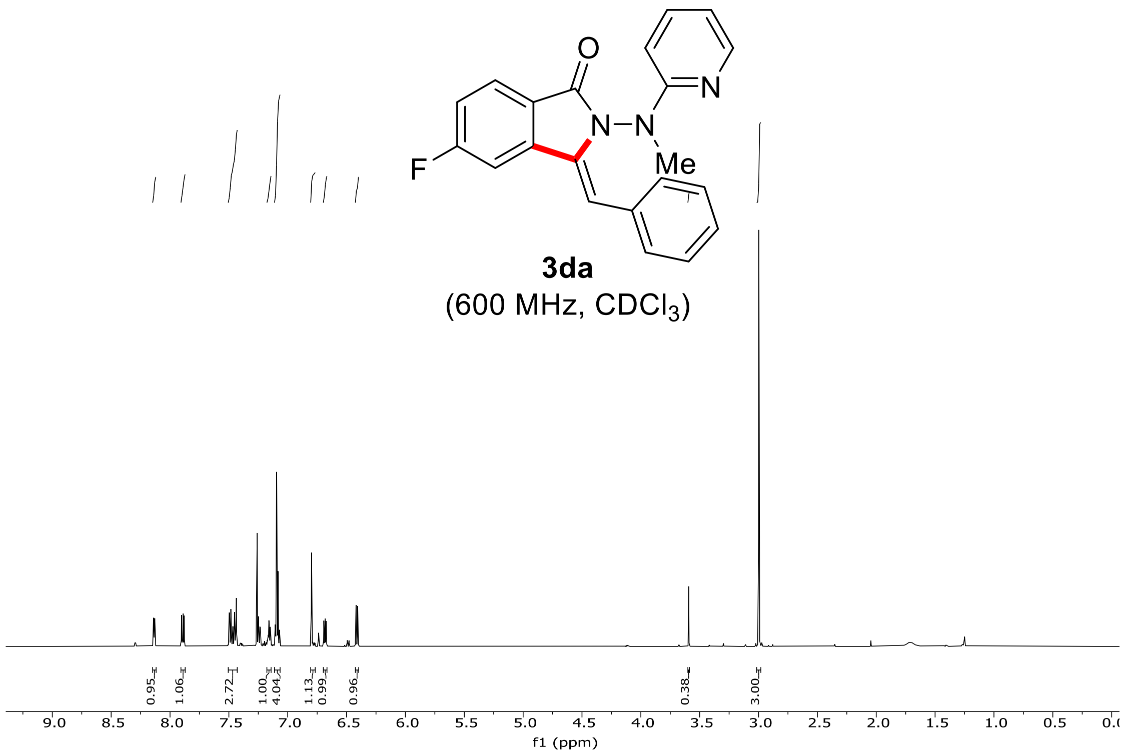
O
N
N–N
Me
F
3da
(600 MHz, $CDCl_3$)
0.95
1.06
2.72
1.00
4.04
1.13
0.99
0.96
0.38
3.00
f1 (ppm)

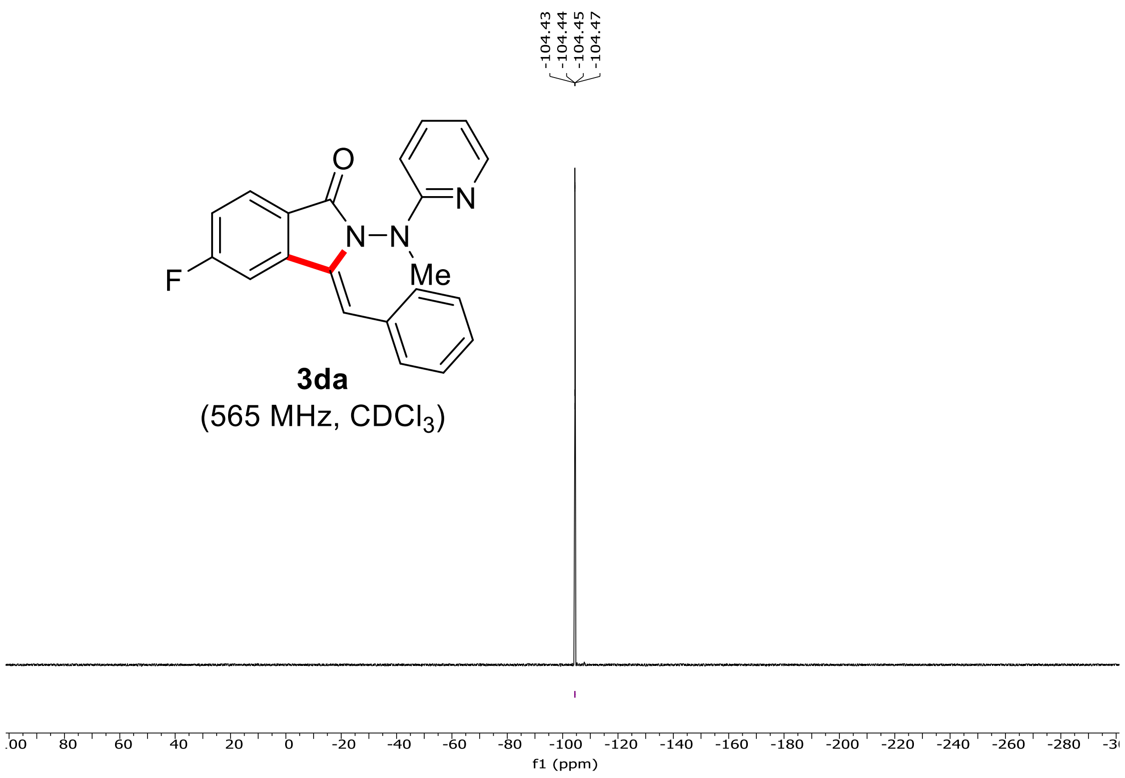
-104.43
-104.44
-104.45
-104.47
O
N
N–N
Me
F
3da
(565 MHz, CDCl3)
f1 (ppm)

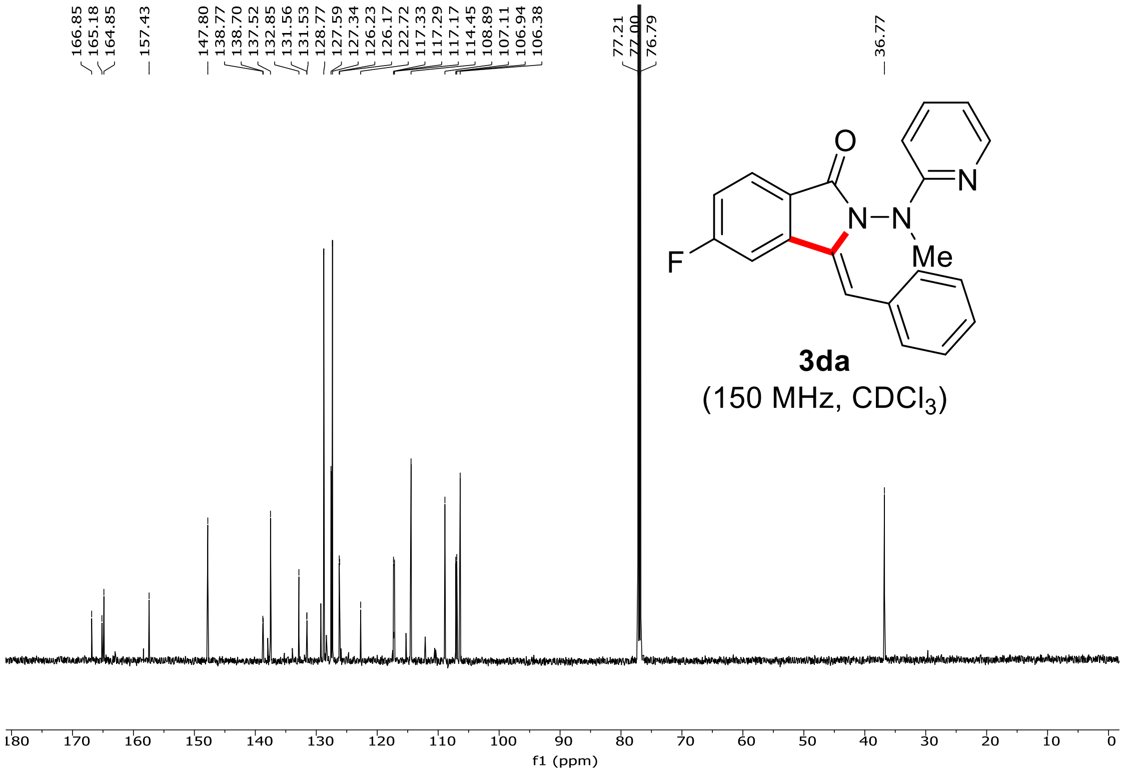
166.85
165.18
164.85
157.43
147.80
138.77
138.70
137.52
132.85
131.56
131.53
128.77
127.59
127.34
126.23
126.17
122.72
117.33
117.29
117.17
114.45
108.89
107.11
106.94
106.38
77.21
77.00
76.79
36.77
O
N
N–N
Me
F
3da
(150 MHz, CDCl3)
f1 (ppm)

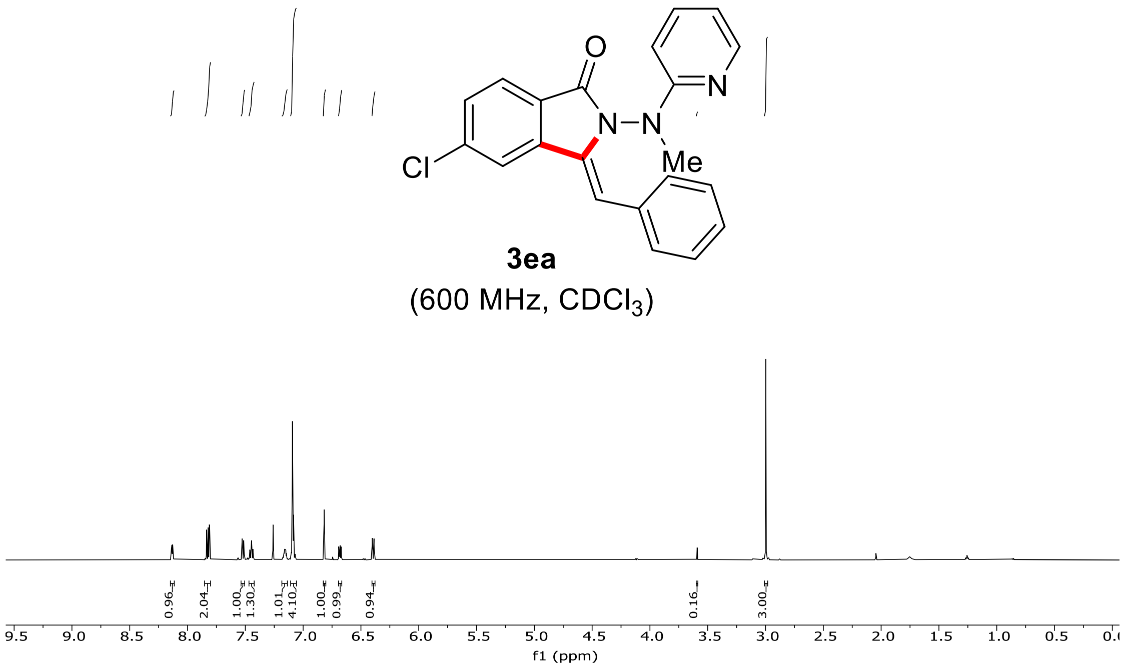

Cl
O
N
N
N
Me
3ea
(600 MHz, CDCl3)
0.96
2.04
1.00
1.30
1.01
4.10
1.00
0.99
0.94
0.16
3.00
9.5 9.0 8.5 8.0 7.5 7.0 6.5 6.0 5.5 5.0 4.5 4.0 3.5 3.0 2.5 2.0 1.5 1.0 0.5
f1 (ppm)

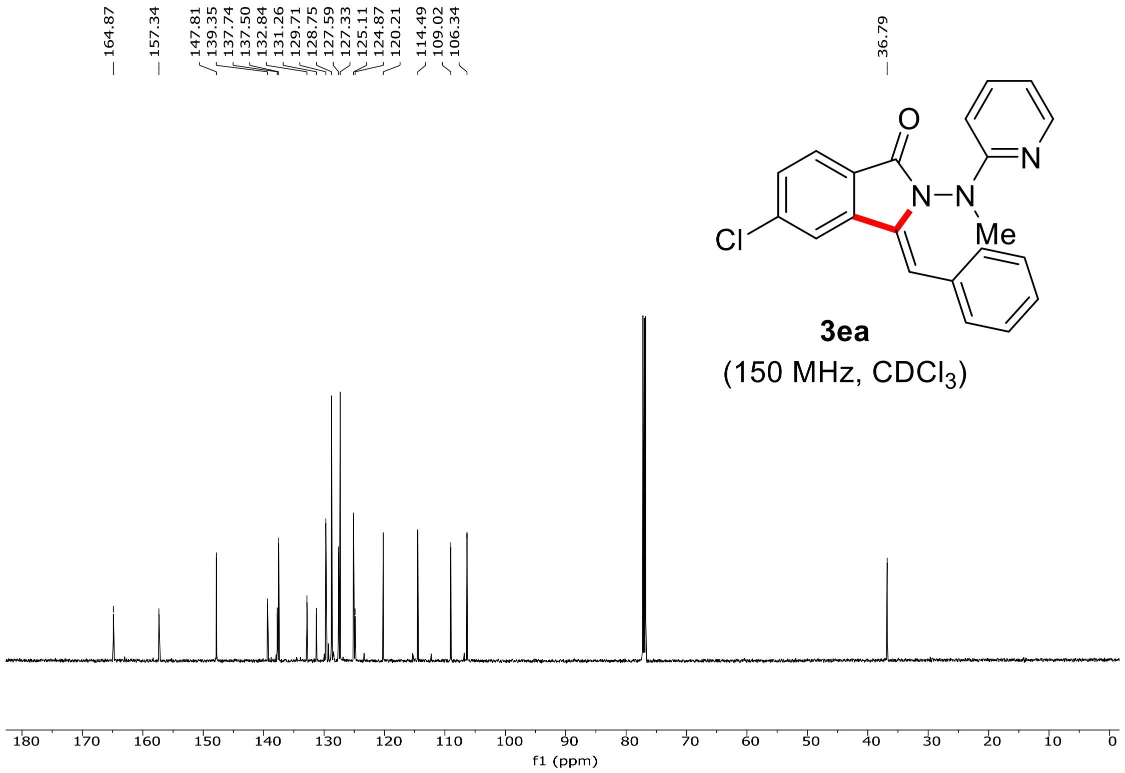

164.87
157.34
147.81
139.35
137.74
137.50
132.84
131.26
129.71
128.75
127.59
127.33
125.11
124.87
120.21
114.49
109.02
106.34
36.79
Cl
O
N
N
N
Me
3ea
(150 MHz, CDCl3)
180 170 160 150 140 130 120 110 100 90 80 70 60 50 40 30 20 10 0
f1 (ppm)

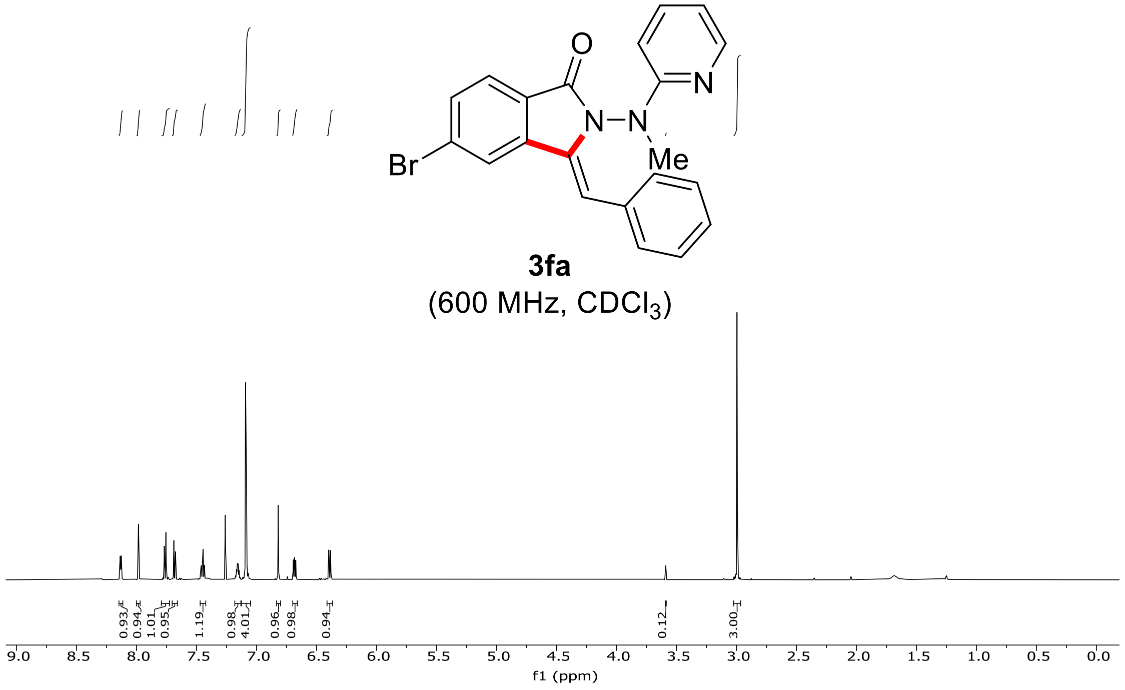

O
N
N
N
Me
Br
3fa
(600 MHz, CDCl3)
0.93
0.94
1.01
0.95
1.19
0.98
4.01
0.96
0.98
0.94
0.12
3.00
9.0 8.5 8.0 7.5 7.0 6.5 6.0 5.5 5.0 4.5 4.0 3.5 3.0 2.5 2.0 1.5 1.0 0.5 0.0
f1 (ppm)

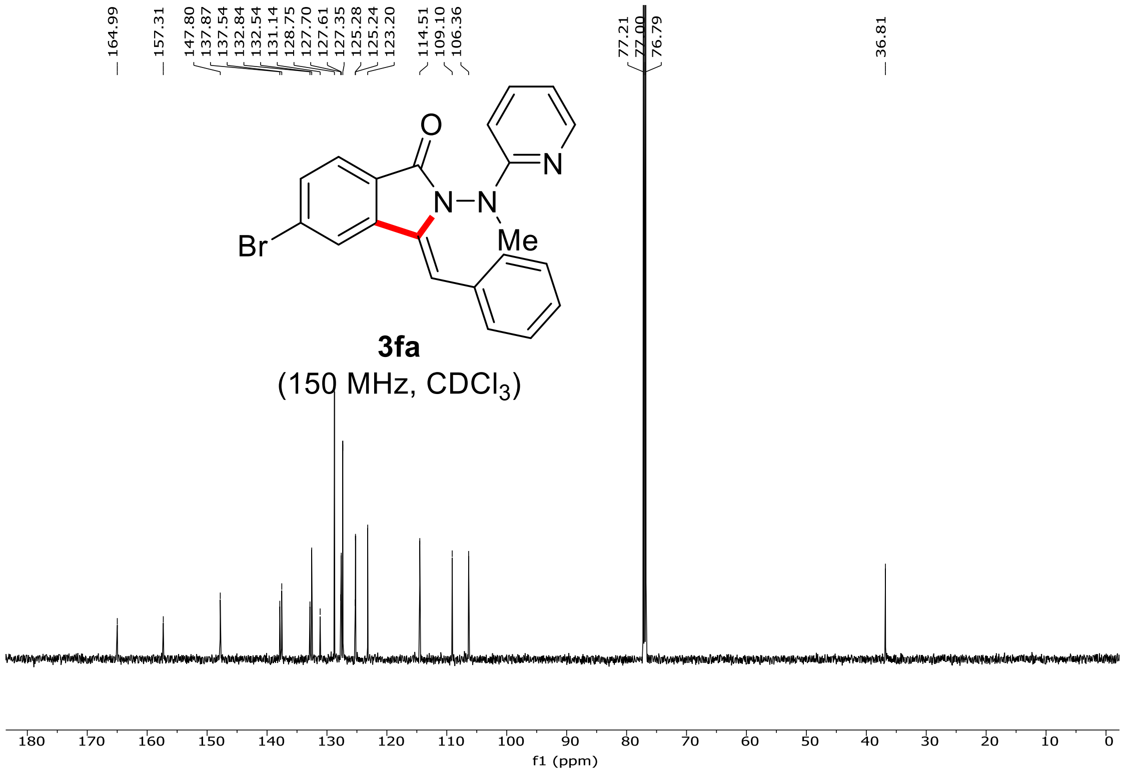

164.99
157.31
147.80
137.87
137.54
132.84
132.54
131.14
128.75
127.70
127.61
127.35
125.28
125.24
123.20
114.51
109.10
106.36
77.21
77.00
76.79
36.81
O
N
N
N
Me
Br
3fa
(150 MHz, CDCl3)
180 170 160 150 140 130 120 110 100 90 80 70 60 50 40 30 20 10 0
f1 (ppm)

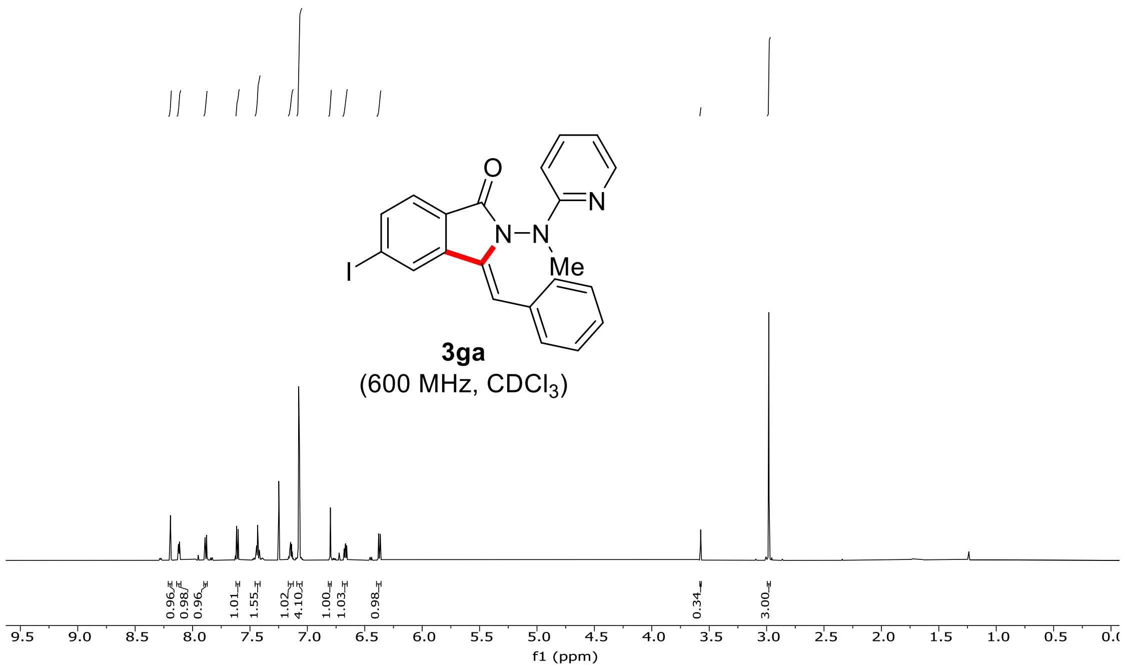
O
N
N–N
Me
I
3ga
(600 MHz, $CDCl_3$)
0.96
0.98
0.96
1.01
1.55
1.02
4.10
1.00
1.03
0.98
0.34
3.00
9.5 9.0 8.5 8.0 7.5 7.0 6.5 6.0 5.5 5.0 4.5 4.0 3.5 3.0 2.5 2.0 1.5 1.0 0.5 0.0
f1 (ppm)

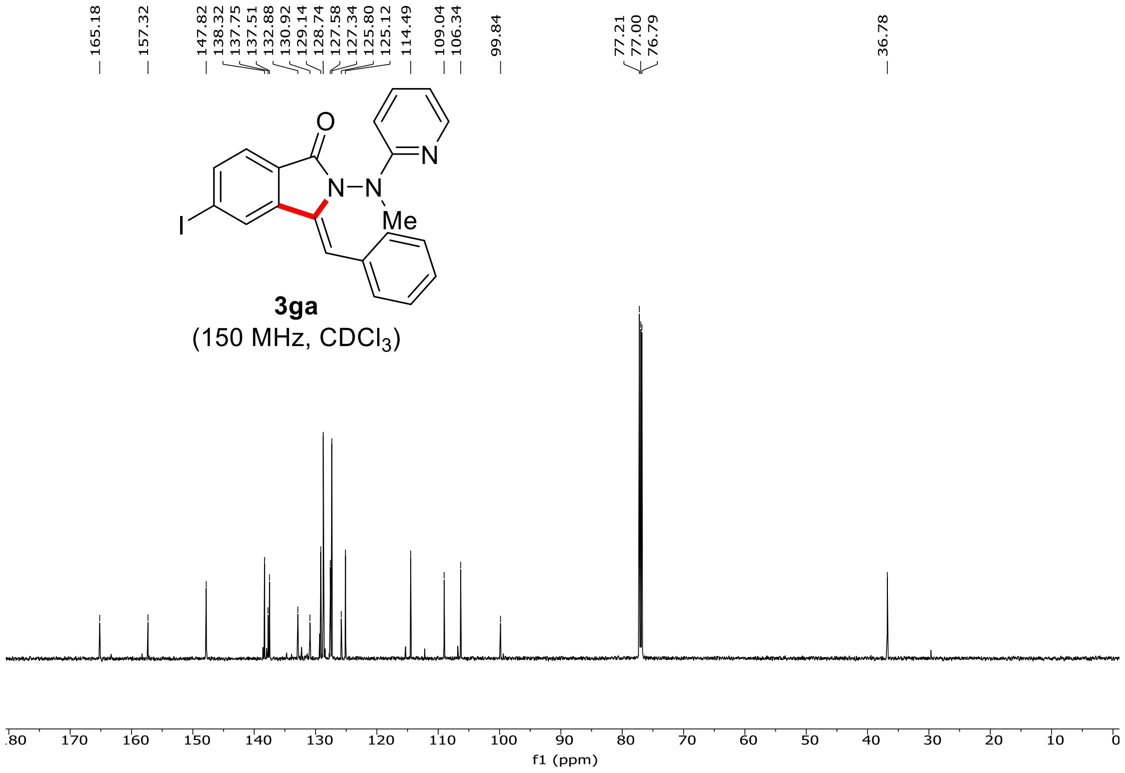
165.18
157.32
147.82
138.32
137.75
137.51
132.88
130.92
129.14
128.74
127.58
127.34
125.80
125.12
114.49
109.04
106.34
99.84
77.21
77.00
76.79
36.78
O
N
N–N
Me
I
3ga
(150 MHz, $CDCl_3$)
180 170 160 150 140 130 120 110 100 90 80 70 60 50 40 30 20 10 0
f1 (ppm)

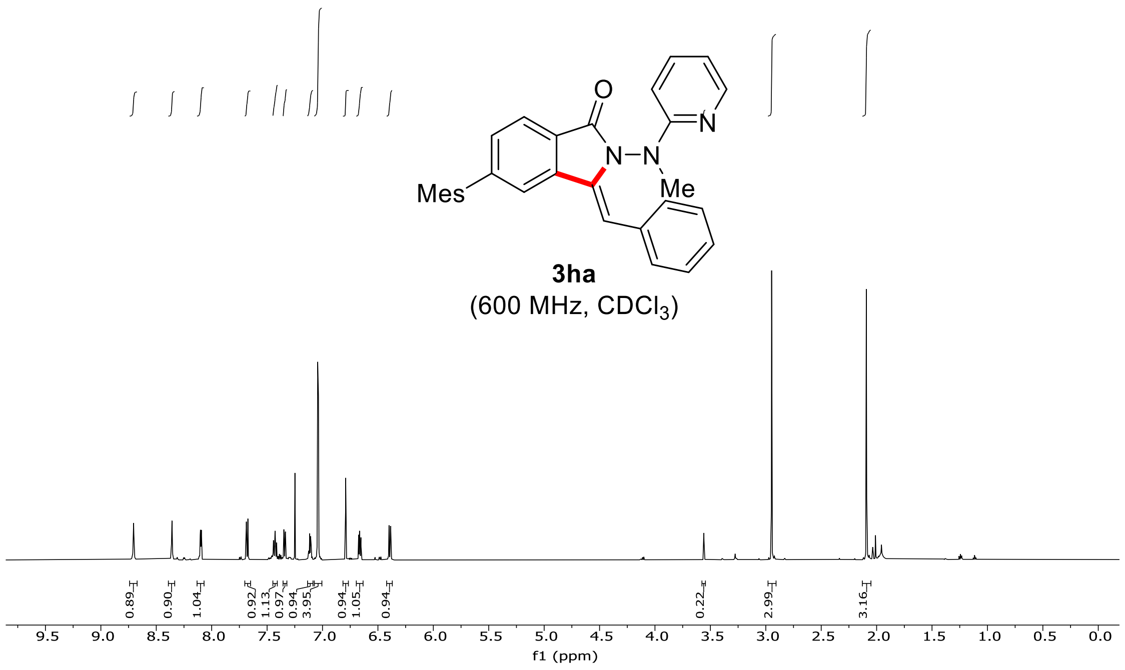
O
N
N–N
Me
Mes
3ha
(600 MHz, CDCl3)
0.89
0.90
1.04
0.92
1.13
0.97
0.94
3.95
0.94
1.05
0.94
0.22
2.99
3.16
9.5 9.0 8.5 8.0 7.5 7.0 6.5 6.0 5.5 5.0 4.5 4.0 3.5 3.0 2.5 2.0 1.5 1.0 0.5 0.0
f1 (ppm)

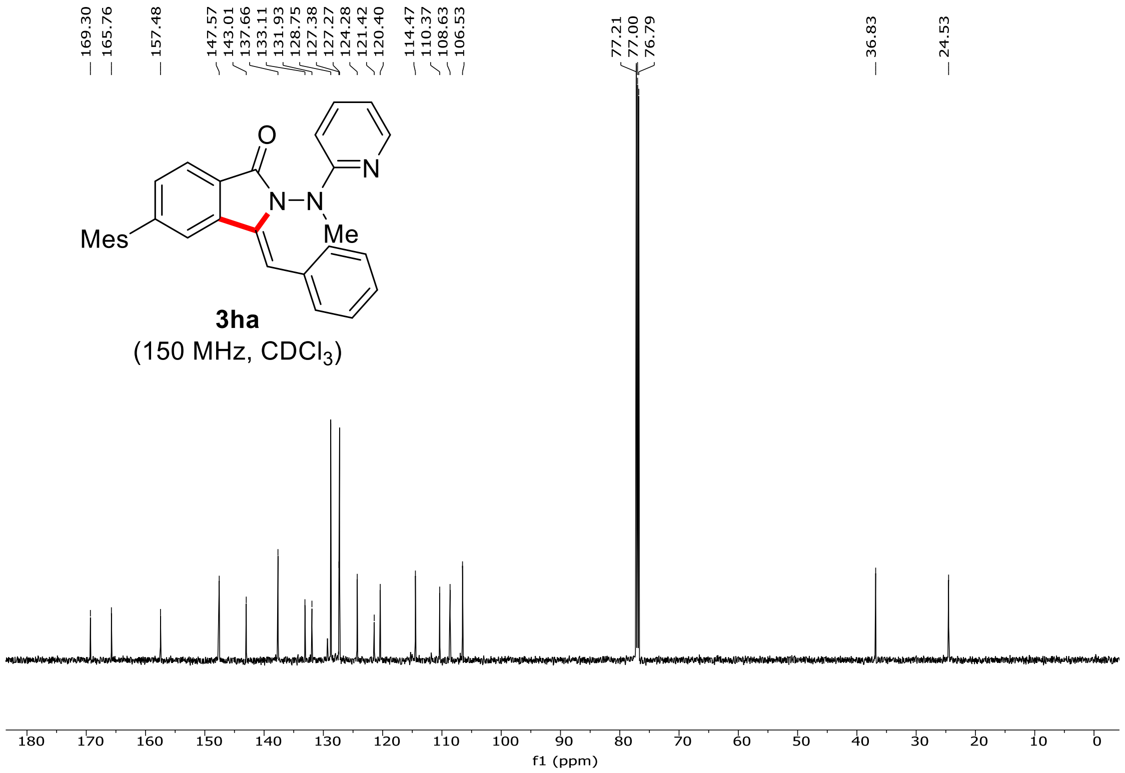
169.30
165.76
157.48
147.57
143.01
137.66
133.11
131.93
128.75
127.38
127.27
124.28
121.42
120.40
114.47
110.37
108.63
106.53
77.21
77.00
76.79
36.83
24.53
O
N
N–N
Me
Mes
3ha
(150 MHz, CDCl3)
180 170 160 150 140 130 120 110 100 90 80 70 60 50 40 30 20 10 0
f1 (ppm)

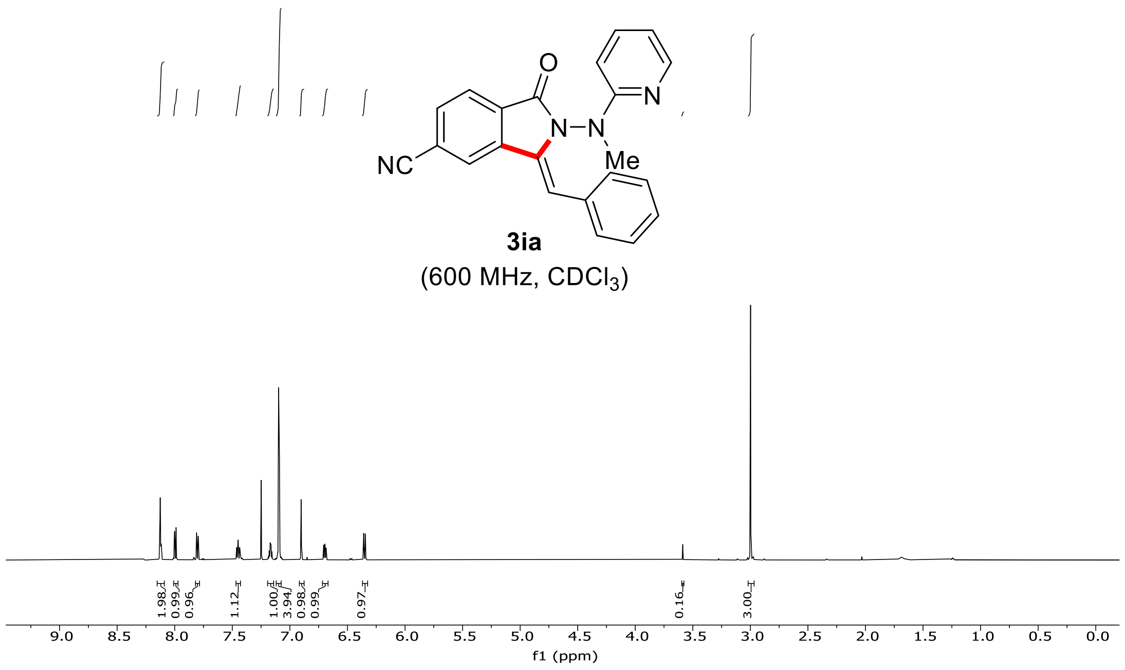
NC
O
N–N
N
Me
3ia
(600 MHz, CDCl3)
1.98
0.99
0.96
1.12
1.00
3.94
0.98
0.99
0.97
0.16
3.00
9.0 8.5 8.0 7.5 7.0 6.5 6.0 5.5 5.0 4.5 4.0 3.5 3.0 2.5 2.0 1.5 1.0 0.5 0.0
f1 (ppm)

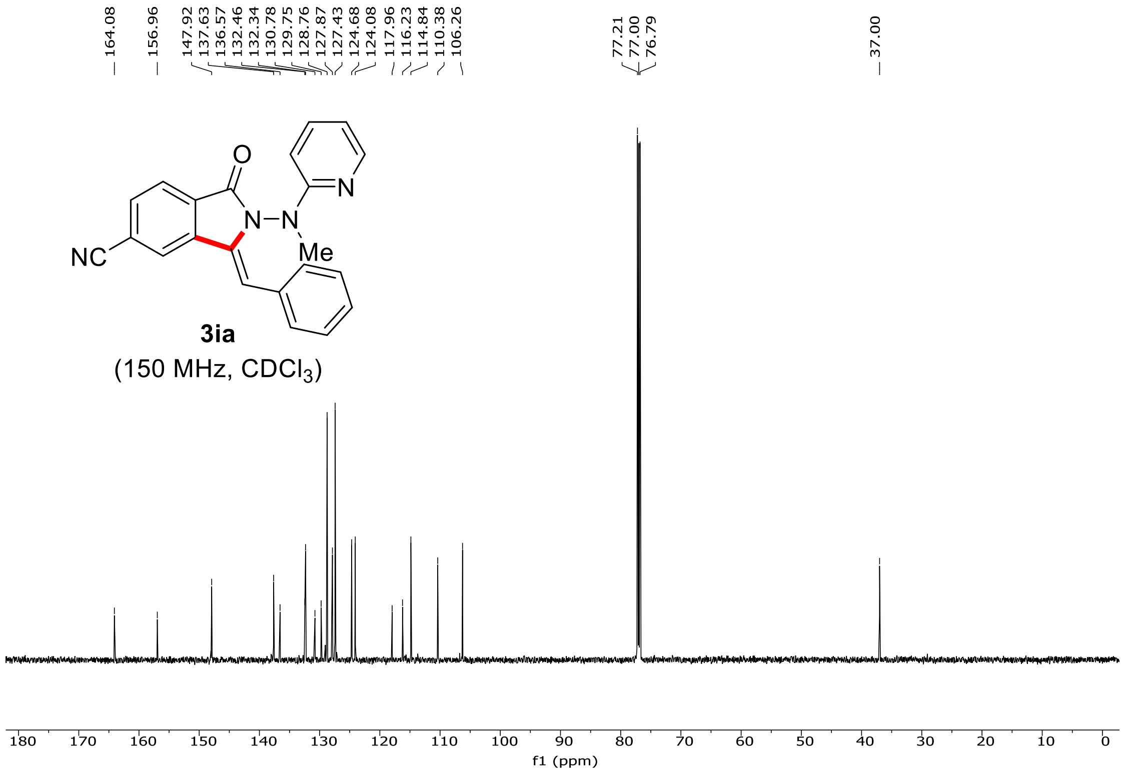
164.08
156.96
147.92
137.63
136.57
132.46
132.34
130.78
129.75
128.76
127.87
127.43
124.68
124.08
117.96
116.23
114.84
110.38
106.26
77.21
77.00
76.79
37.00
NC
O
N–N
N
Me
3ia
(150 MHz, CDCl3)
180 170 160 150 140 130 120 110 100 90 80 70 60 50 40 30 20 10 0
f1 (ppm)

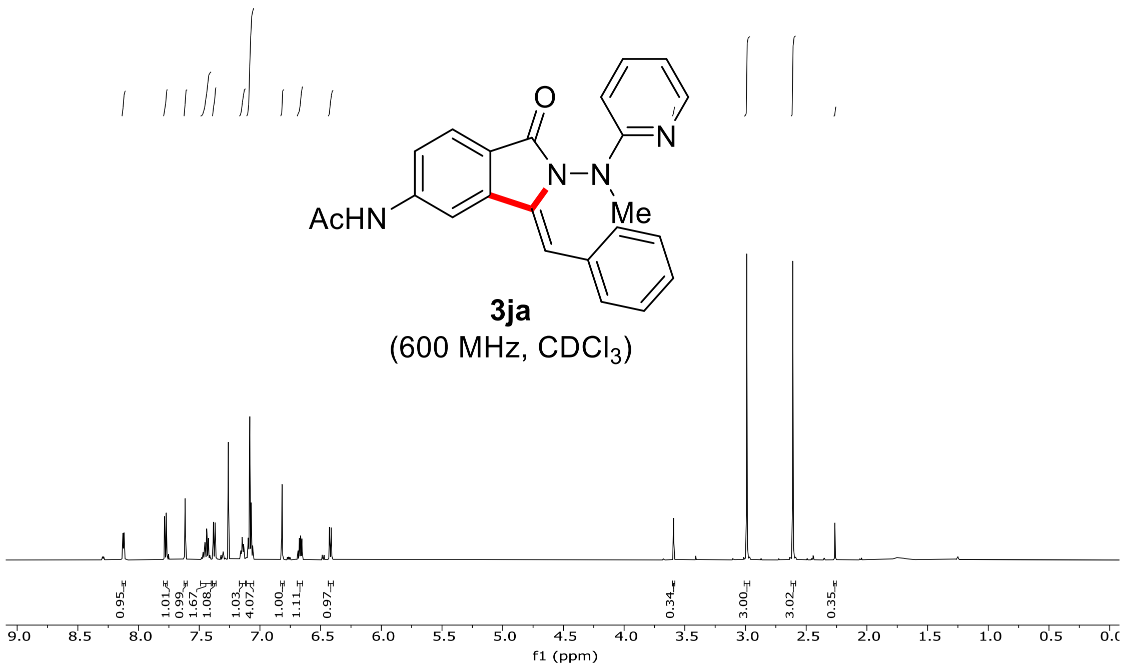
O
N
N–N
AcHN
Me
3ja
(600 MHz, CDCl$_3$)
0.95 1.01 0.99 1.67 1.08 1.03 4.07 1.00 1.11 0.97 0.34 3.00 3.02 0.35
9.0 8.5 8.0 7.5 7.0 6.5 6.0 5.5 5.0 4.5 4.0 3.5 3.0 2.5 2.0 1.5 1.0 0.5
f1 (ppm)

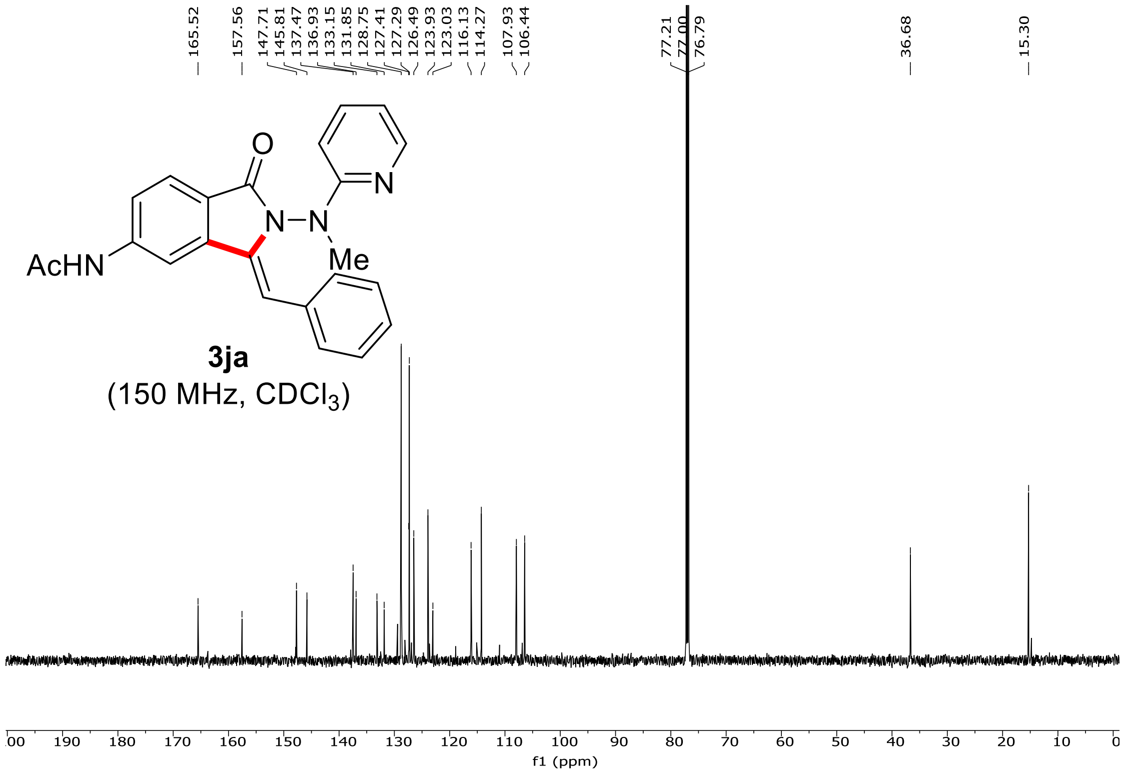
165.52
157.56
147.71
145.81
137.47
136.93
133.15
131.85
128.75
127.41
127.29
126.49
123.93
123.03
116.13
114.27
107.93
106.44
77.21
77.00
76.79
36.68
15.30
O
N
N–N
AcHN
Me
3ja
(150 MHz, CDCl$_3$)
190 180 170 160 150 140 130 120 110 100 90 80 70 60 50 40 30 20 10 0
f1 (ppm)

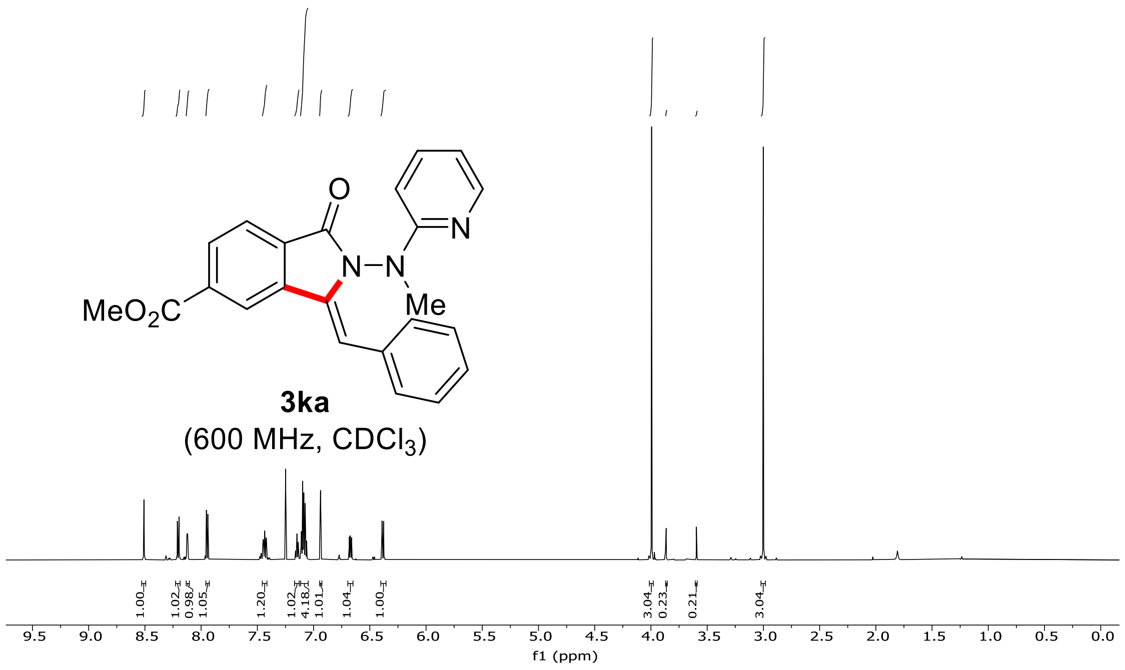
O
N
N–N
Me
MeO2C
3ka
(600 MHz, CDCl3)
1.00
1.02
0.98
1.05
1.20
1.02
4.18
1.01
1.04
1.00
3.04
0.23
0.21
3.04
9.5 9.0 8.5 8.0 7.5 7.0 6.5 6.0 5.5 5.0 4.5 4.0 3.5 3.0 2.5 2.0 1.5 1.0 0.5 0.0
f1 (ppm)

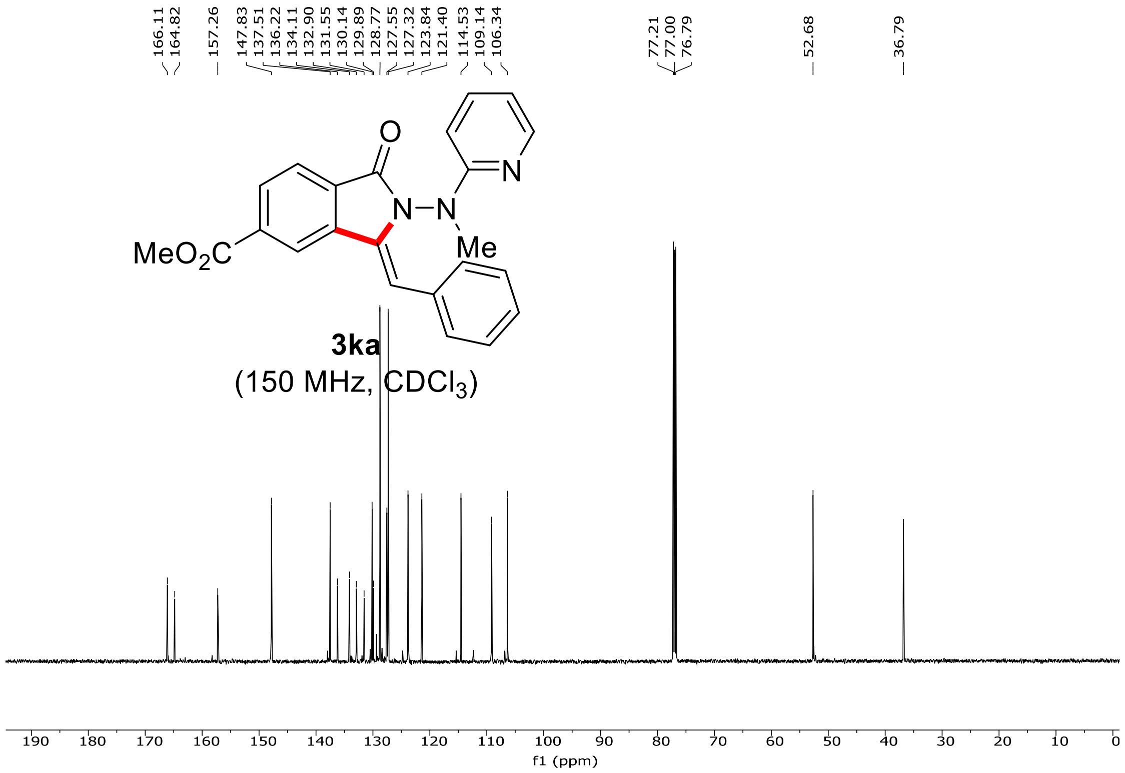
166.11
164.82
157.26
147.83
137.51
136.22
134.11
132.90
131.55
130.14
129.89
128.77
127.55
127.32
123.84
121.40
114.53
109.14
106.34
77.21
77.00
76.79
52.68
36.79
O
N
N–N
Me
MeO2C
3ka
(150 MHz, CDCl3)
190 180 170 160 150 140 130 120 110 100 90 80 70 60 50 40 30 20 10 0
f1 (ppm)

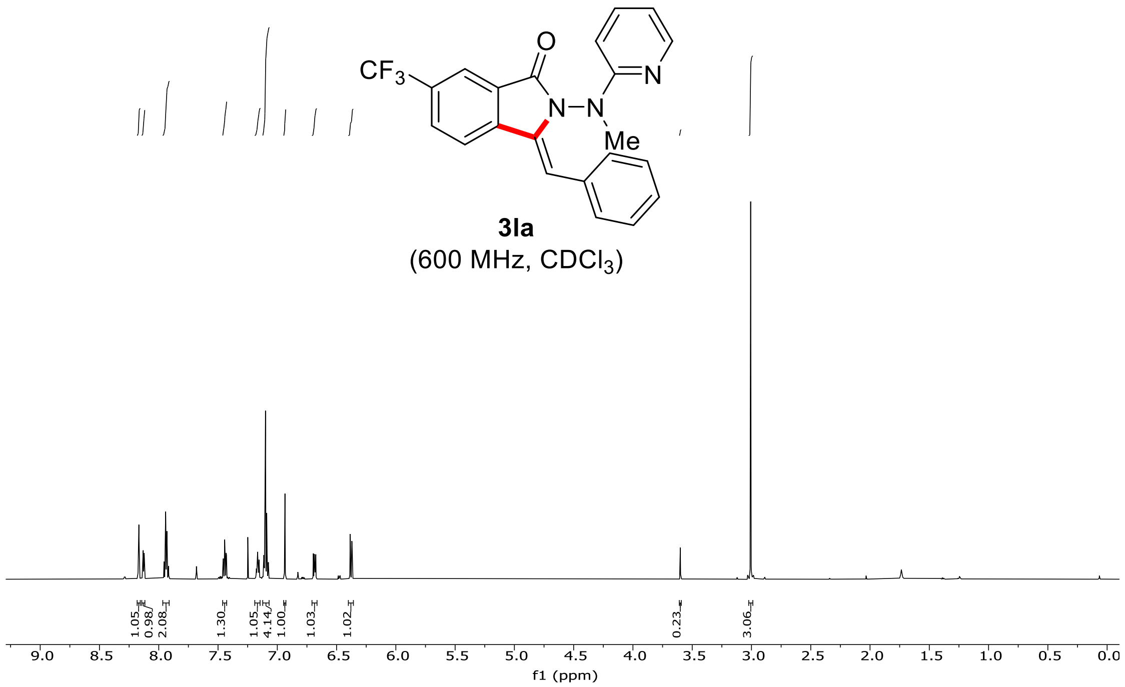
CF3
O
N
N
N
Me
3la
(600 MHz, CDCl3)
1.05
0.98
2.08
1.30
1.05
4.14
1.00
1.03
1.02
0.23
3.06
9.0
8.5
8.0
7.5
7.0
6.5
6.0
5.5
5.0
4.5
4.0
3.5
3.0
2.5
2.0
1.5
1.0
0.5
0.0
f1 (ppm)

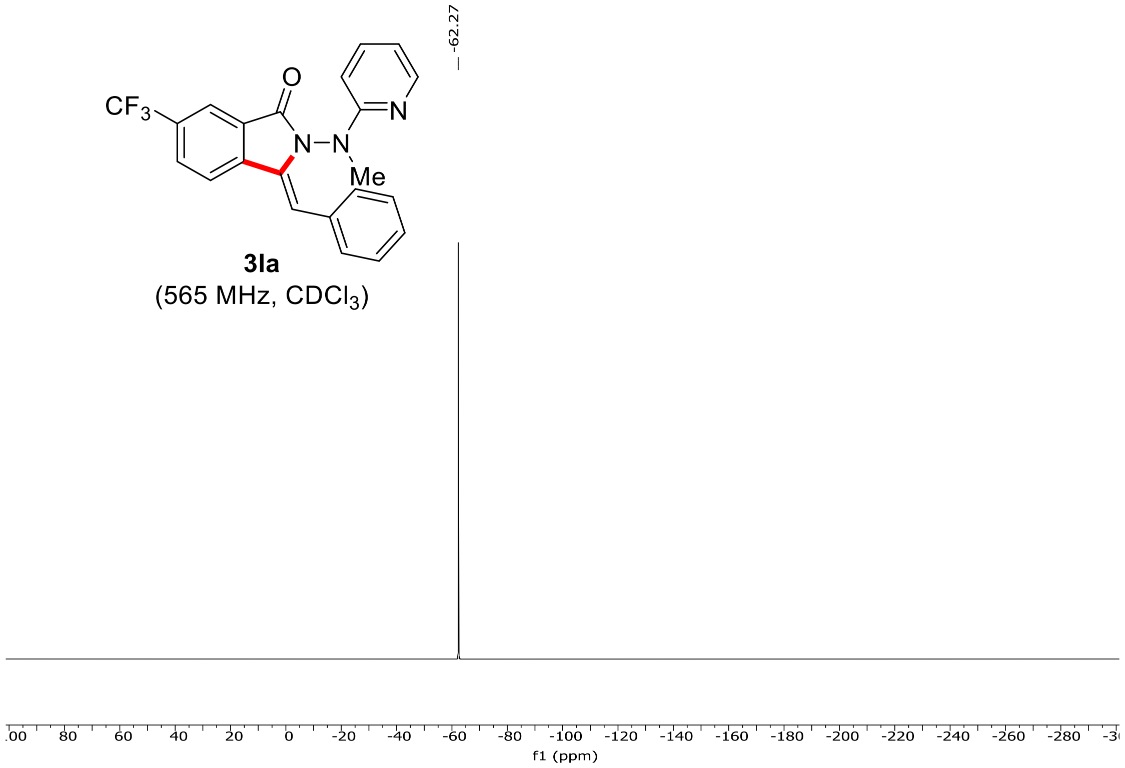
-62.27
CF3
O
N
N
N
Me
3la
(565 MHz, CDCl3)
80
60
40
20
0
-20
-40
-60
-80
-100
-120
-140
-160
-180
-200
-220
-240
-260
-280
f1 (ppm)

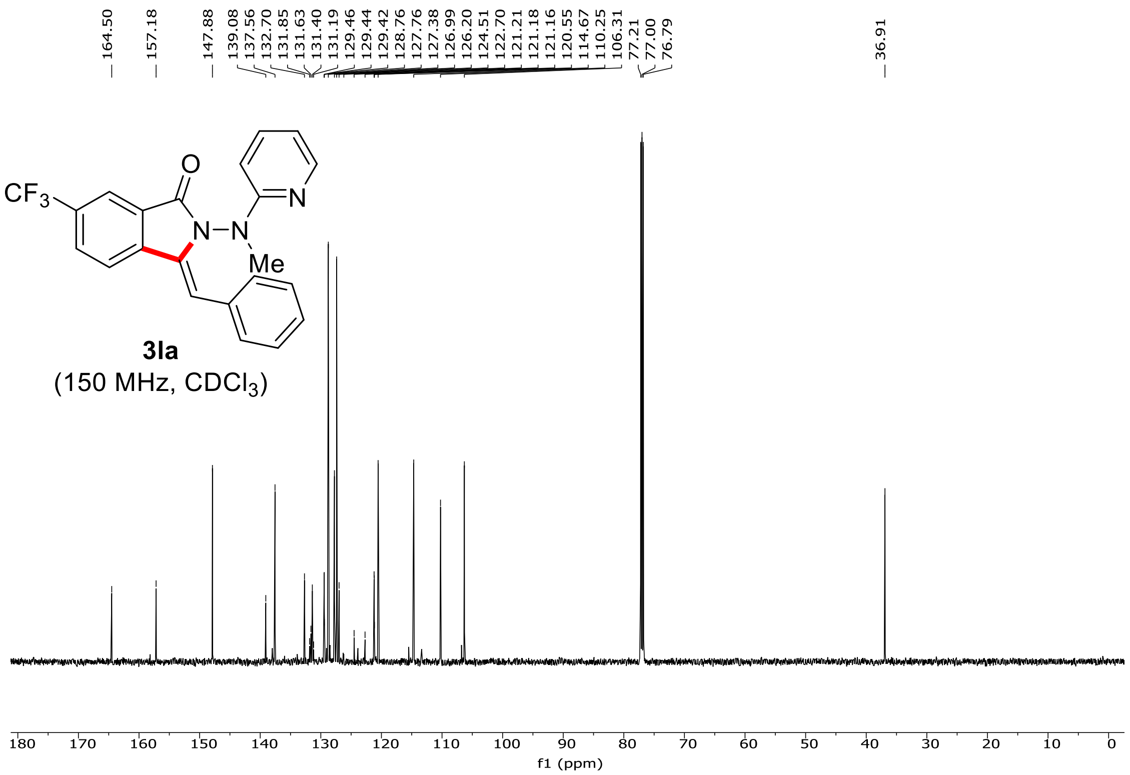
164.50
157.18
147.88
139.08
137.56
132.70
131.85
131.63
131.40
131.19
129.46
129.44
129.42
128.76
127.76
127.38
126.99
126.20
124.51
122.70
121.21
121.18
121.16
120.55
114.67
110.25
106.31
77.21
77.00
76.79
36.91
CF3
O
N
N
N
Me
3la
(150 MHz, CDCl3)
f1 (ppm)

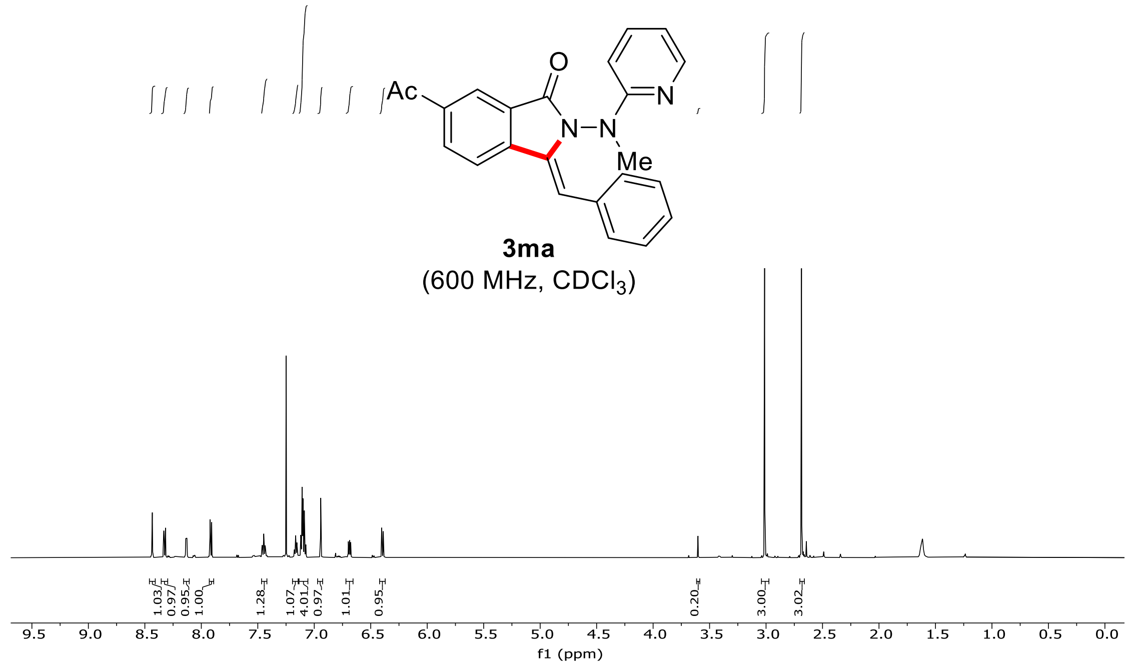
Ac
O
N
N
N
Me
3ma
(600 MHz, CDCl3)
1.03
0.97
0.95
1.00
1.28
1.07
4.01
0.97
1.01
0.95
0.20
3.00
3.02
f1 (ppm)

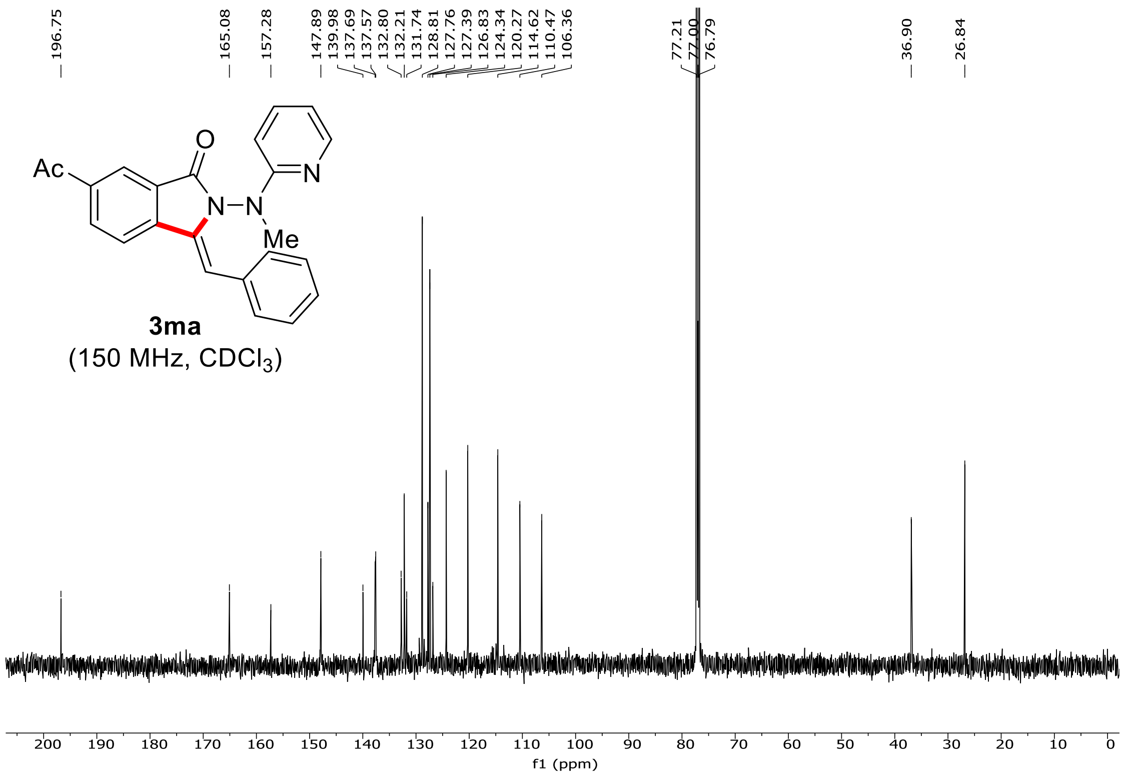
196.75
165.08
157.28
147.89
139.98
137.69
137.57
132.80
132.21
131.74
128.81
127.76
127.39
126.83
124.34
120.27
114.62
110.47
106.36
77.21
77.00
76.79
36.90
26.84
Ac
O
N
N
Me
3ma
(150 MHz, CDCl3)
200 190 180 170 160 150 140 130 120 110 100 90 80 70 60 50 40 30 20 10 0
f1 (ppm)

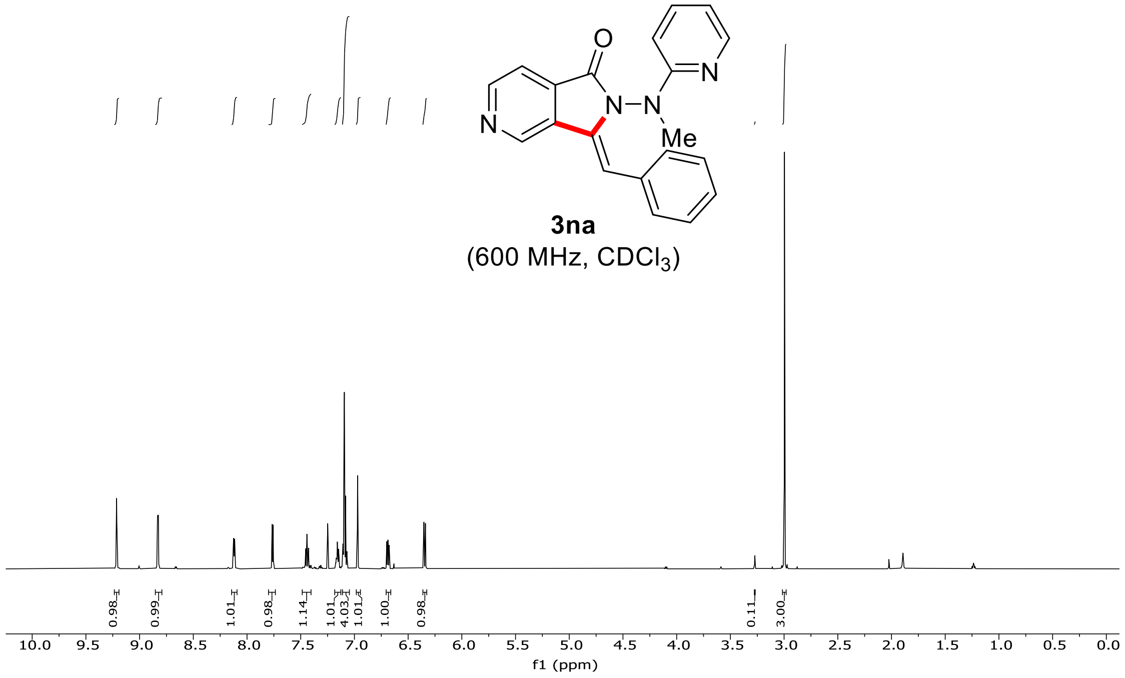
O
N
N
N
Me
3na
(600 MHz, CDCl3)
0.98
0.99
1.01
0.98
1.14
1.01
4.03
1.01
1.00
0.98
0.11
3.00
10.0 9.5 9.0 8.5 8.0 7.5 7.0 6.5 6.0 5.5 5.0 4.5 4.0 3.5 3.0 2.5 2.0 1.5 1.0 0.5 0.0
f1 (ppm)

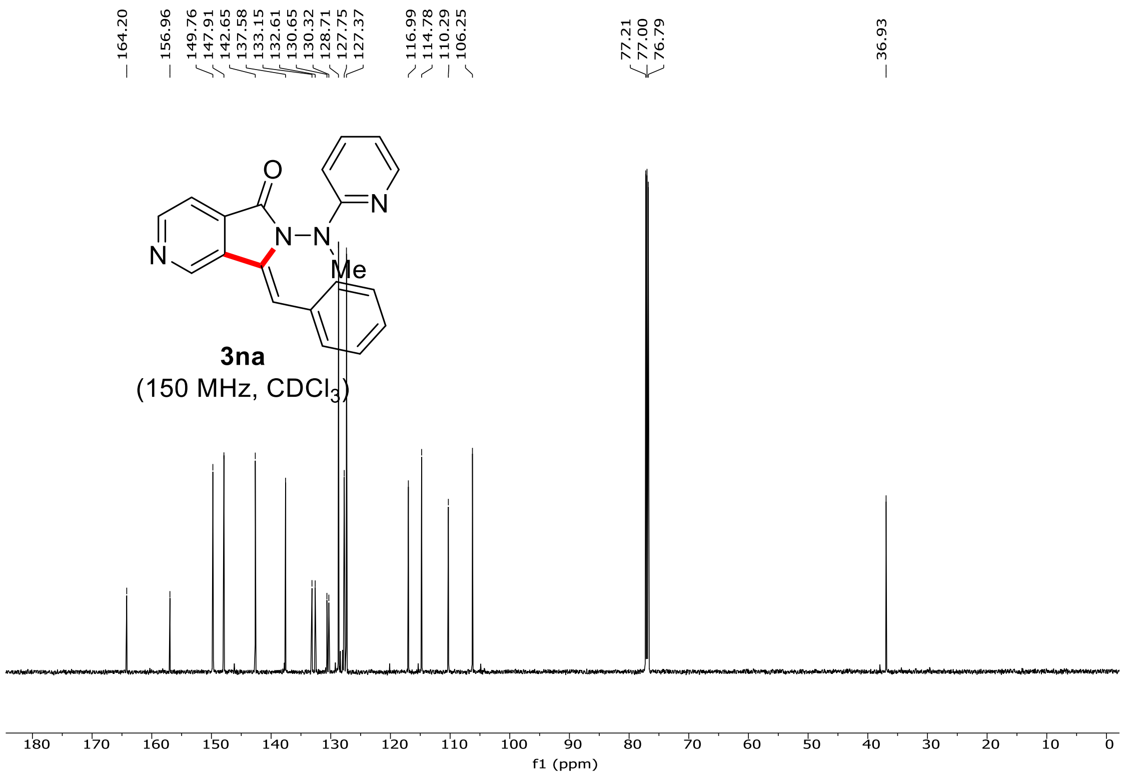
164.20
156.96
149.76
147.91
142.65
137.58
133.15
132.61
130.65
130.32
128.71
127.75
127.37
116.99
114.78
110.29
106.25
77.21
77.00
76.79
36.93
3na
(150 MHz, $CDCl_3$)
f1 (ppm)

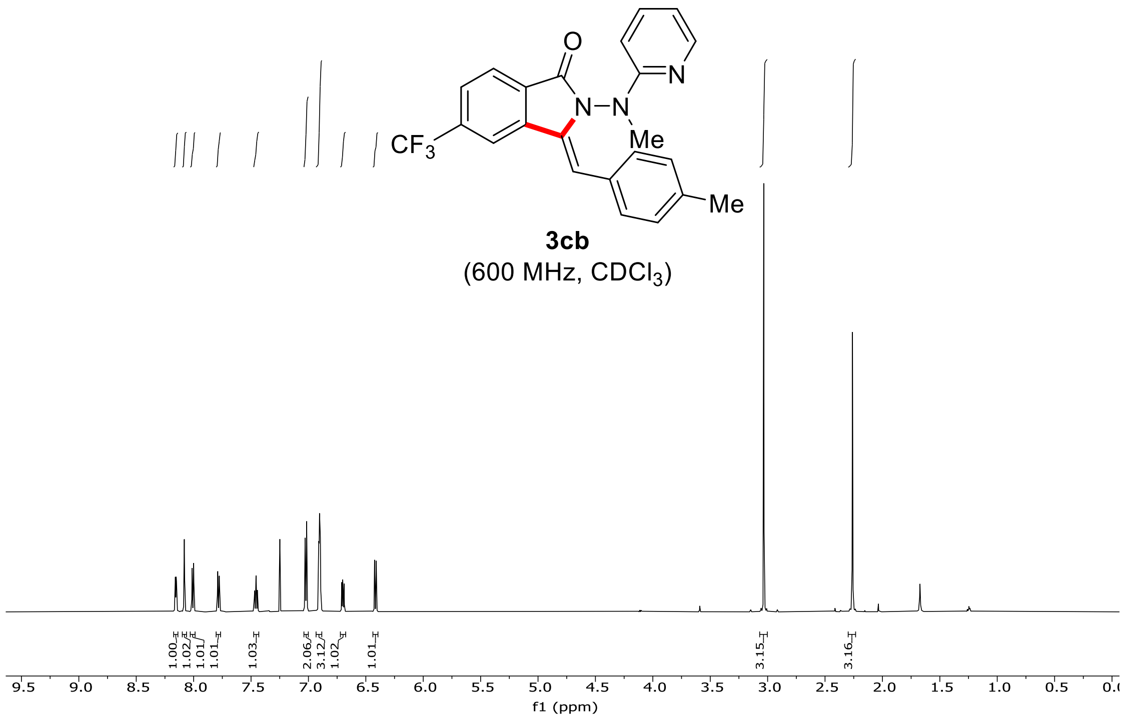
3cb
(600 MHz, $CDCl_3$)
CF_3
Me
f1 (ppm)

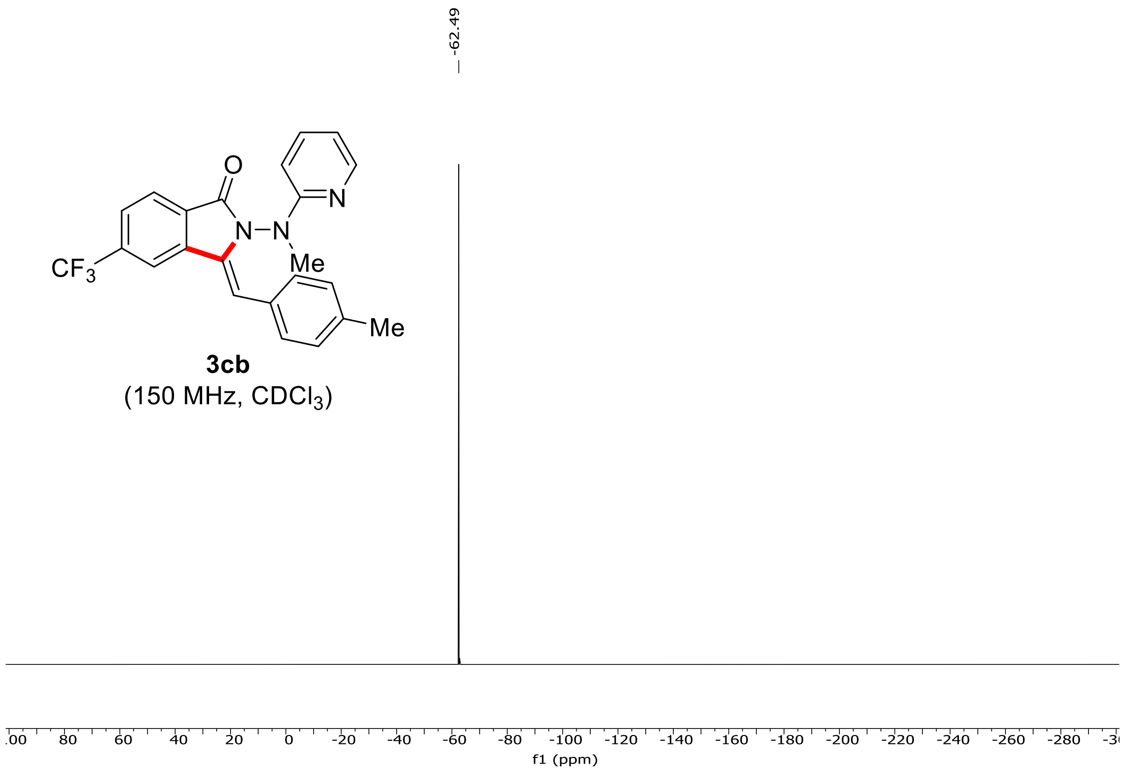
-62.49
O
N
N–N
Me
CF3
Me
3cb
(150 MHz, CDCl3)
00
80
60
40
20
0
-20
-40
-60
-80
-100
-120
-140
-160
-180
-200
-220
-240
-260
-280
f1 (ppm)

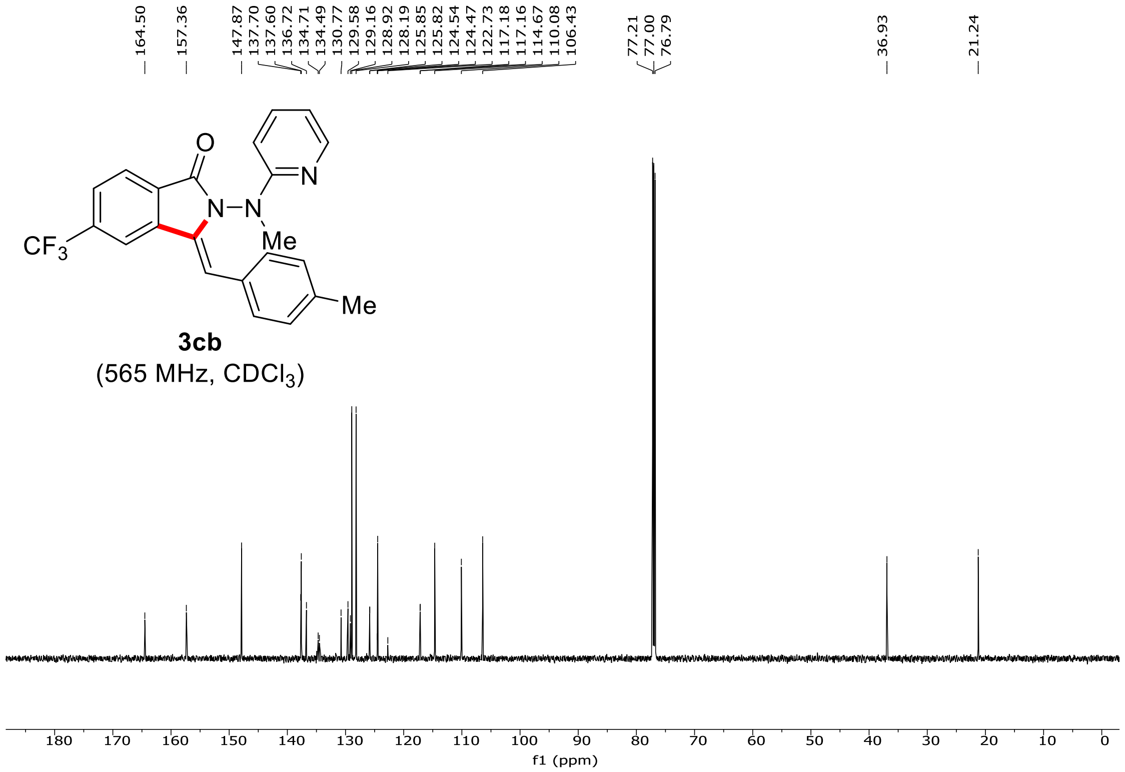
164.50
157.36
147.87
137.70
137.60
136.72
134.71
134.49
130.77
129.58
129.16
128.92
128.19
125.85
125.82
124.54
124.47
122.73
117.18
117.16
114.67
110.08
106.43
77.21
77.00
76.79
36.93
21.24
O
N
N–N
Me
CF3
Me
3cb
(565 MHz, CDCl3)
180
170
160
150
140
130
120
110
100
90
80
70
60
50
40
30
20
10
0
f1 (ppm)

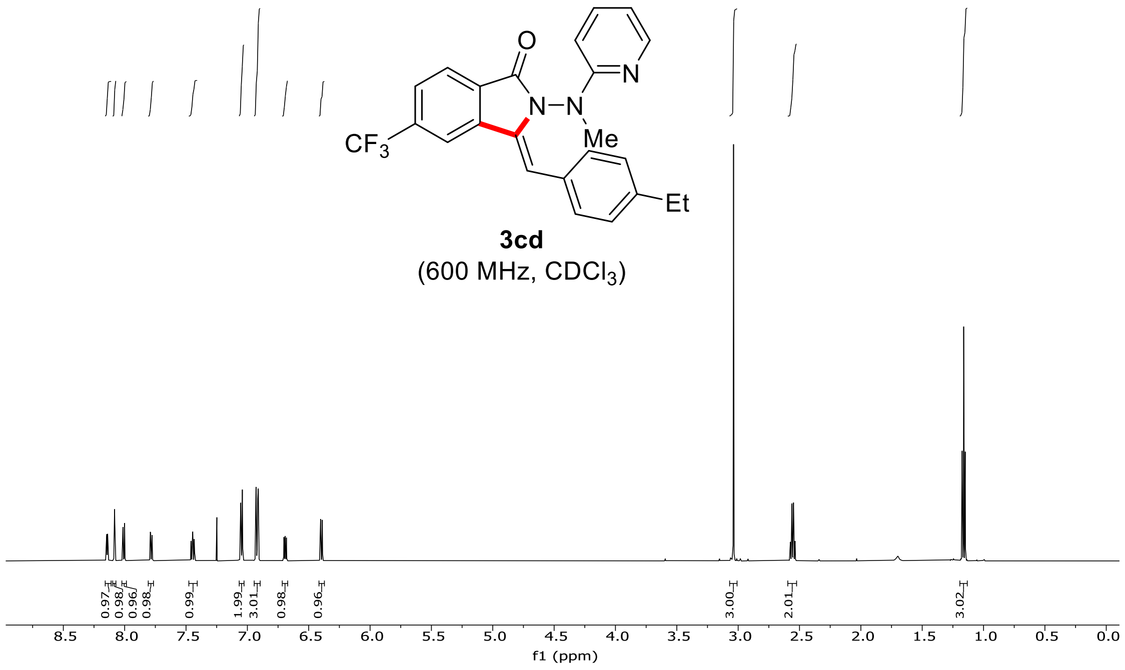
O
N
N–N
Me
CF3
Et
3cd
(600 MHz, CDCl3)
0.97
0.98
0.96
0.98
0.99
1.99
3.01
0.98
0.96
3.00
2.01
3.02
8.5
8.0
7.5
7.0
6.5
6.0
5.5
5.0
4.5
4.0
3.5
3.0
2.5
2.0
1.5
1.0
0.5
0.0
f1 (ppm)

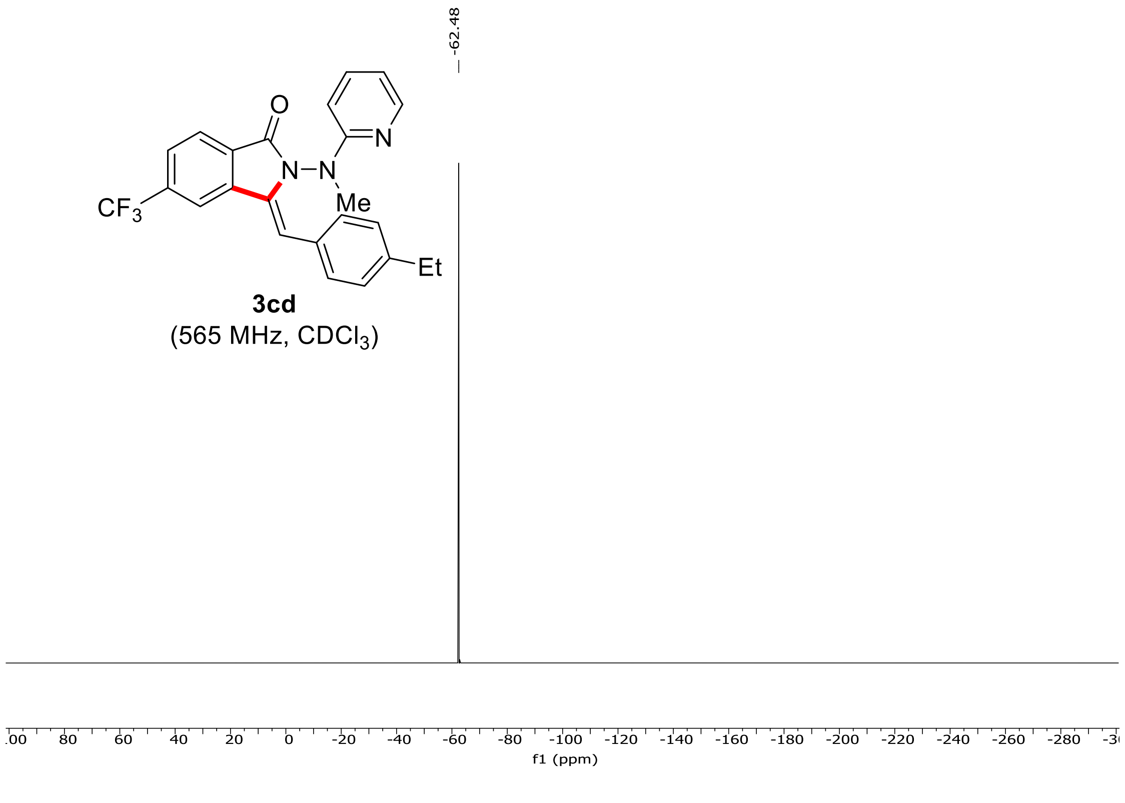
-62.48
O
N
N–N
Me
CF3
Et
3cd
(565 MHz, CDCl3)
80
60
40
20
0
-20
-40
-60
-80
-100
-120
-140
-160
-180
-200
-220
-240
-260
-280
f1 (ppm)

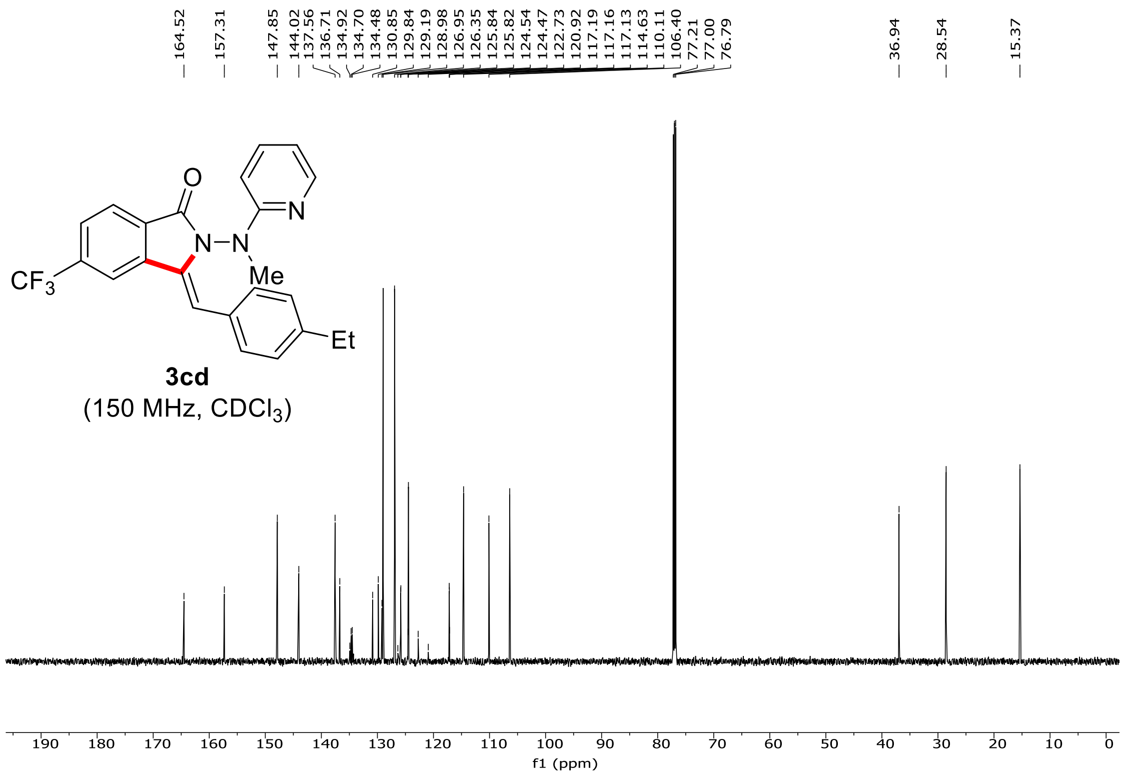
164.52
157.31
147.85
144.02
137.56
136.71
134.92
134.70
134.48
130.85
129.84
129.19
128.98
126.95
126.35
125.84
125.82
124.54
124.47
122.73
120.92
117.19
117.16
117.13
114.63
110.11
106.40
77.21
77.00
76.79
36.94
28.54
15.37
O
N
N–N
Me
CF3
Et
3cd
(150 MHz, CDCl3)
f1 (ppm)

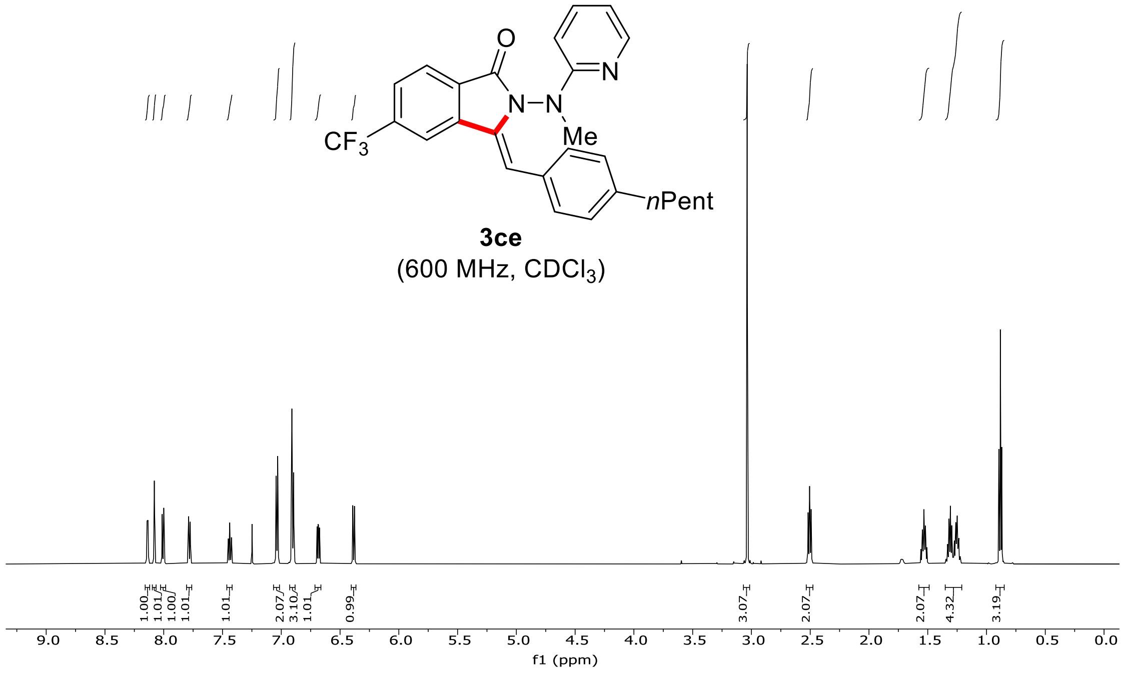
O
N
N–N
Me
CF3
nPent
3ce
(600 MHz, CDCl3)
1.00
1.01
1.00
1.01
1.01
2.07
3.10
1.01
0.99
3.07
2.07
2.07
4.32
3.19
f1 (ppm)

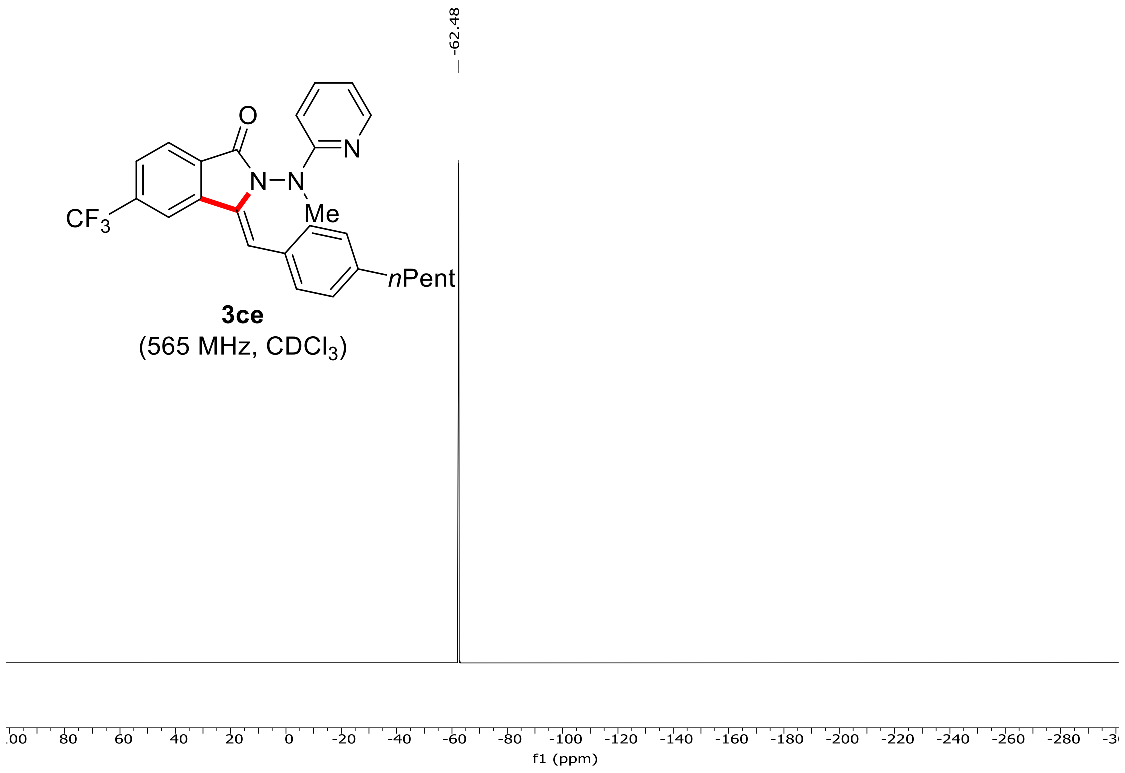
-62.48
O
N
N
N
Me
CF3
nPent
3ce
(565 MHz, CDCl3)
f1 (ppm)

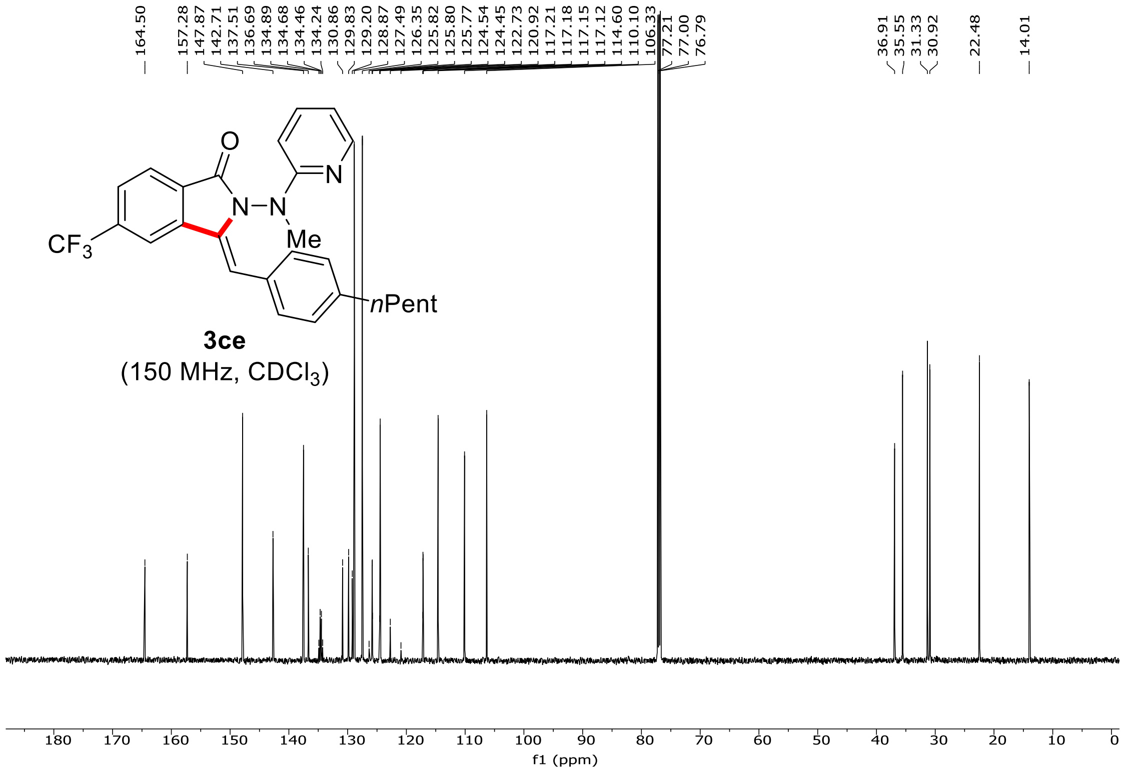
164.50
157.28
147.87
142.71
137.51
136.69
134.89
134.68
134.46
134.24
130.86
129.83
129.20
128.87
127.49
126.35
125.82
125.80
125.77
124.54
124.45
122.73
120.92
117.21
117.18
117.15
117.12
114.60
110.10
106.33
77.21
77.00
76.79
36.91
35.55
31.33
30.92
22.48
14.01
O
N
N
N
Me
CF3
nPent
3ce
(150 MHz, CDCl3)
f1 (ppm)

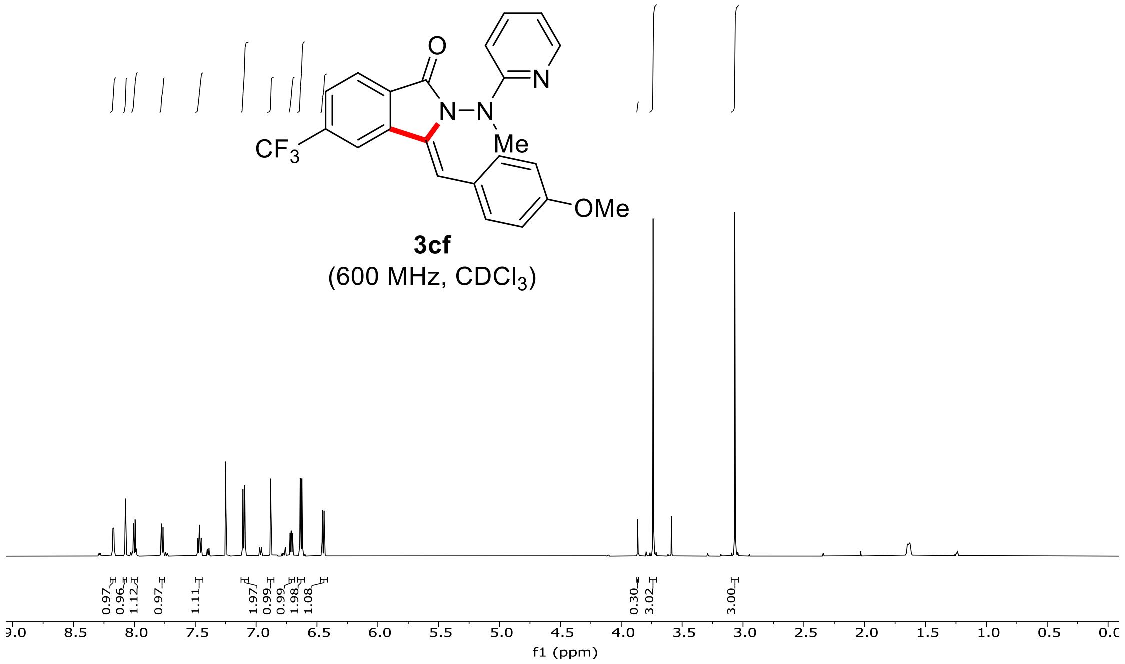
O
N
N
N
Me
CF3
OMe
3cf
(600 MHz, CDCl3)
0.97
0.96
1.12
0.97
1.11
1.97
0.99
0.99
1.98
1.08
0.30
3.02
3.00
f1 (ppm)

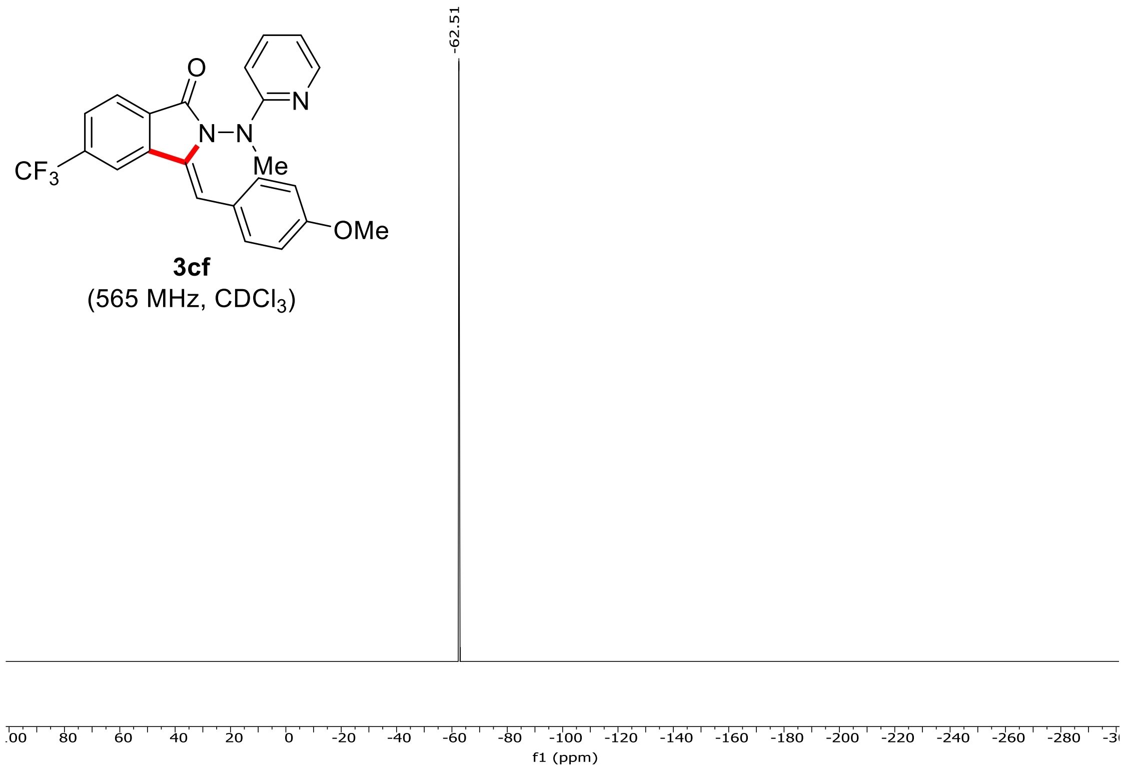
-62.51
O
N
N
N
Me
CF3
OMe
3cf
(565 MHz, CDCl3)
f1 (ppm)

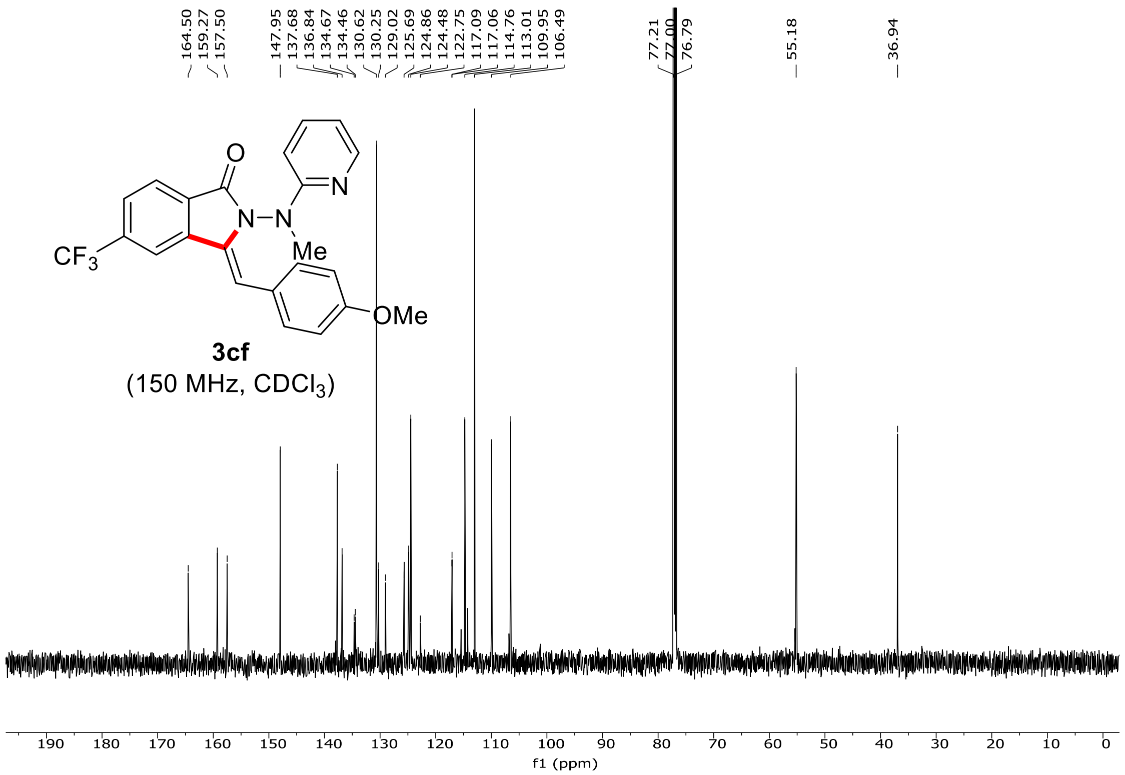
164.50
159.27
157.50
147.95
137.68
136.84
134.67
134.46
130.62
130.25
129.02
125.69
124.86
124.48
122.75
117.09
117.06
114.76
113.01
109.95
106.49
77.21
77.00
76.79
55.18
36.94
O
N
N
N
Me
CF_3
OMe
3cf
(150 MHz, $CDCl_3$)
190 180 170 160 150 140 130 120 110 100 90 80 70 60 50 40 30 20 10 0
f1 (ppm)

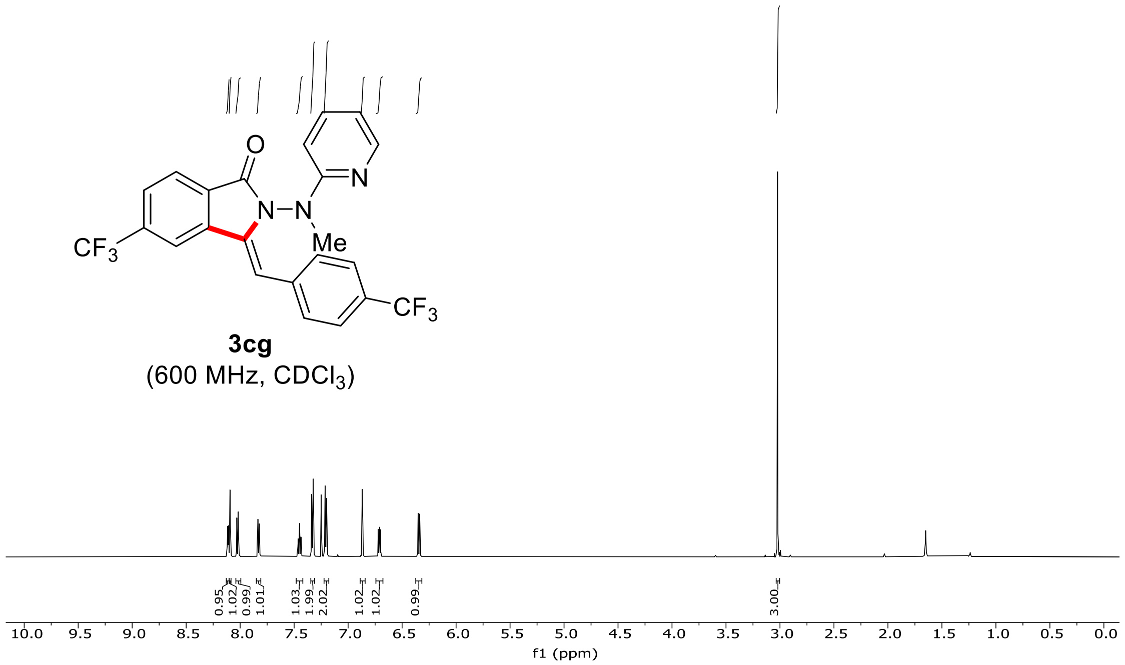
O
N
N
N
Me
CF_3
CF_3
3cg
(600 MHz, $CDCl_3$)
0.95
1.02
0.99
1.01
1.03
1.99
2.02
1.02
1.02
0.99
3.00
10.0 9.5 9.0 8.5 8.0 7.5 7.0 6.5 6.0 5.5 5.0 4.5 4.0 3.5 3.0 2.5 2.0 1.5 1.0 0.5 0.0
f1 (ppm)

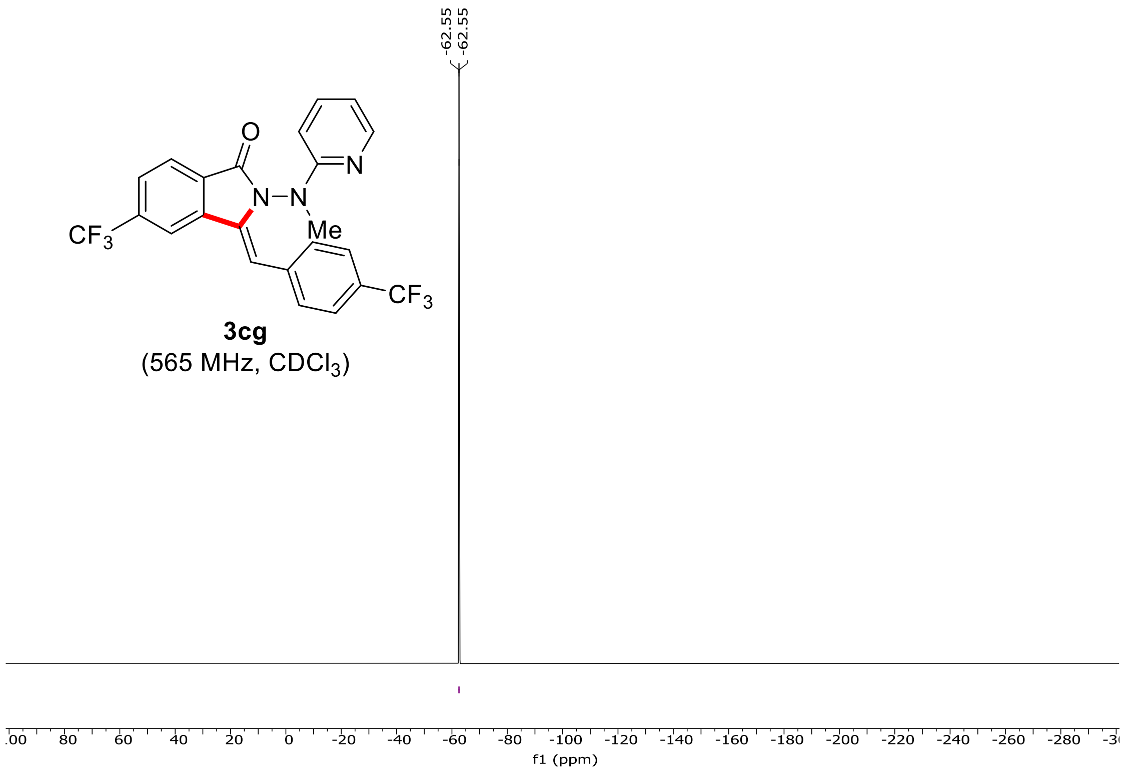
-62.55
-62.55
O
N
N
N
Me
CF3
CF3
3cg
(565 MHz, CDCl3)
f1 (ppm)

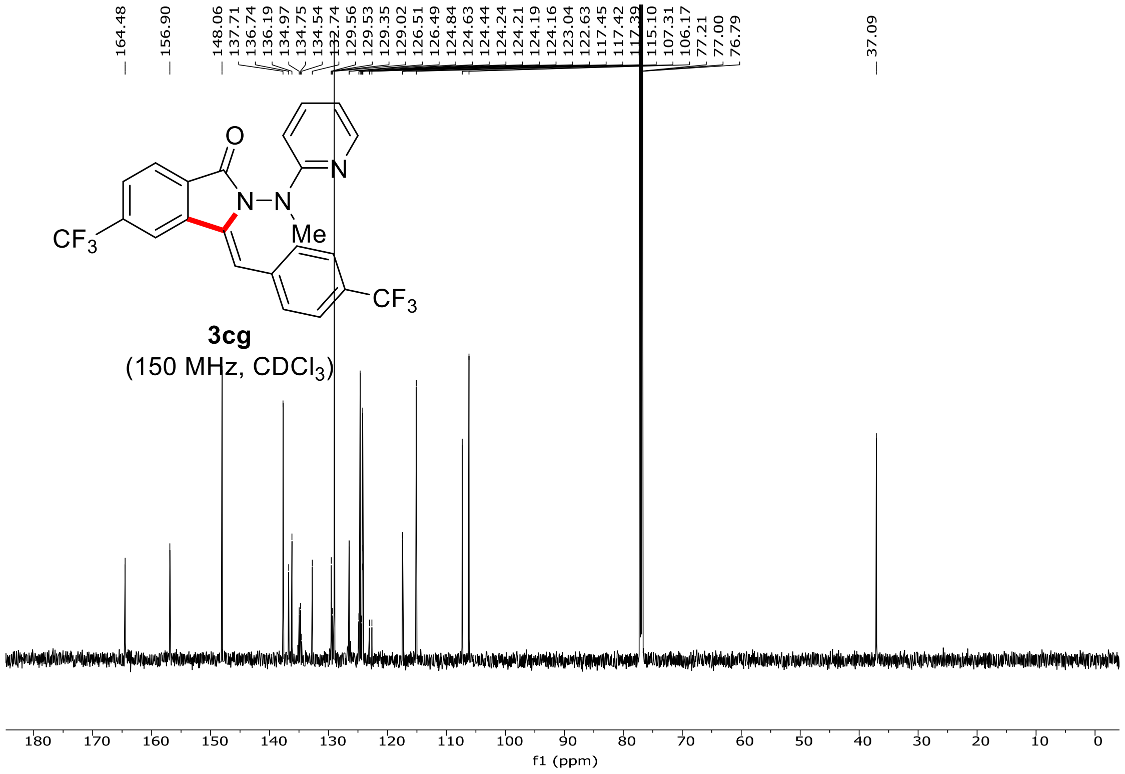
164.48
156.90
148.06
137.71
136.74
136.19
134.97
134.75
134.54
132.74
129.56
129.53
129.35
129.02
126.51
126.49
124.84
124.63
124.44
124.24
124.21
124.19
124.16
123.04
122.63
117.45
117.42
117.39
115.10
107.31
106.17
77.21
77.00
76.79
37.09
O
N
N
N
Me
CF3
CF3
3cg
(150 MHz, CDCl3)
f1 (ppm)

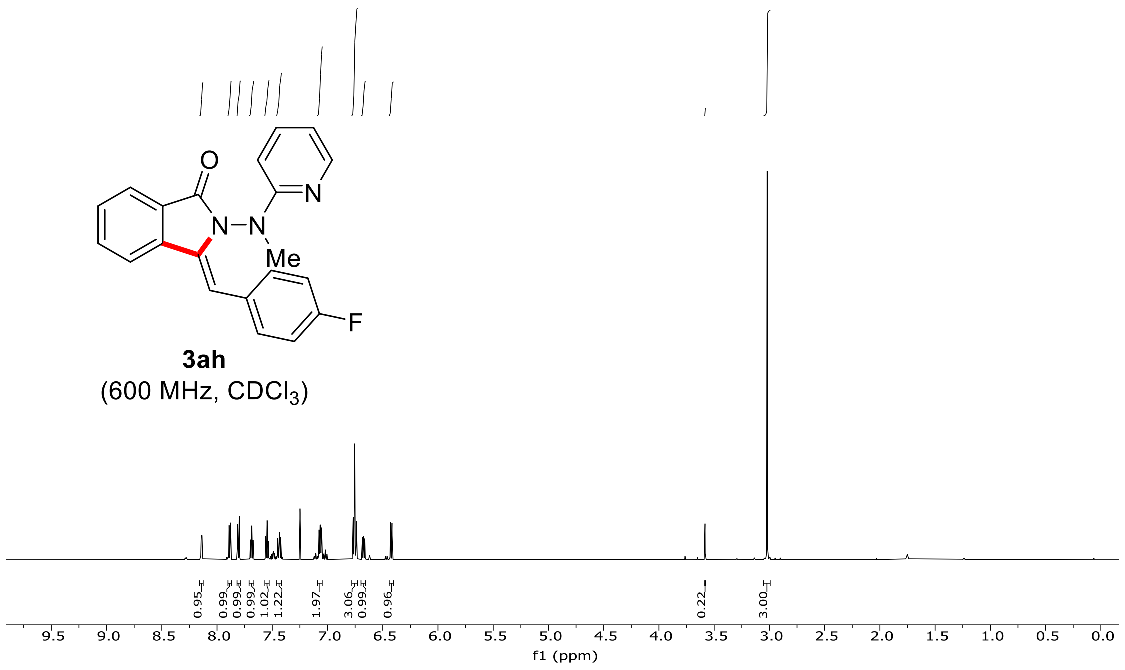

3ah
(600 MHz, $CDCl_3$)

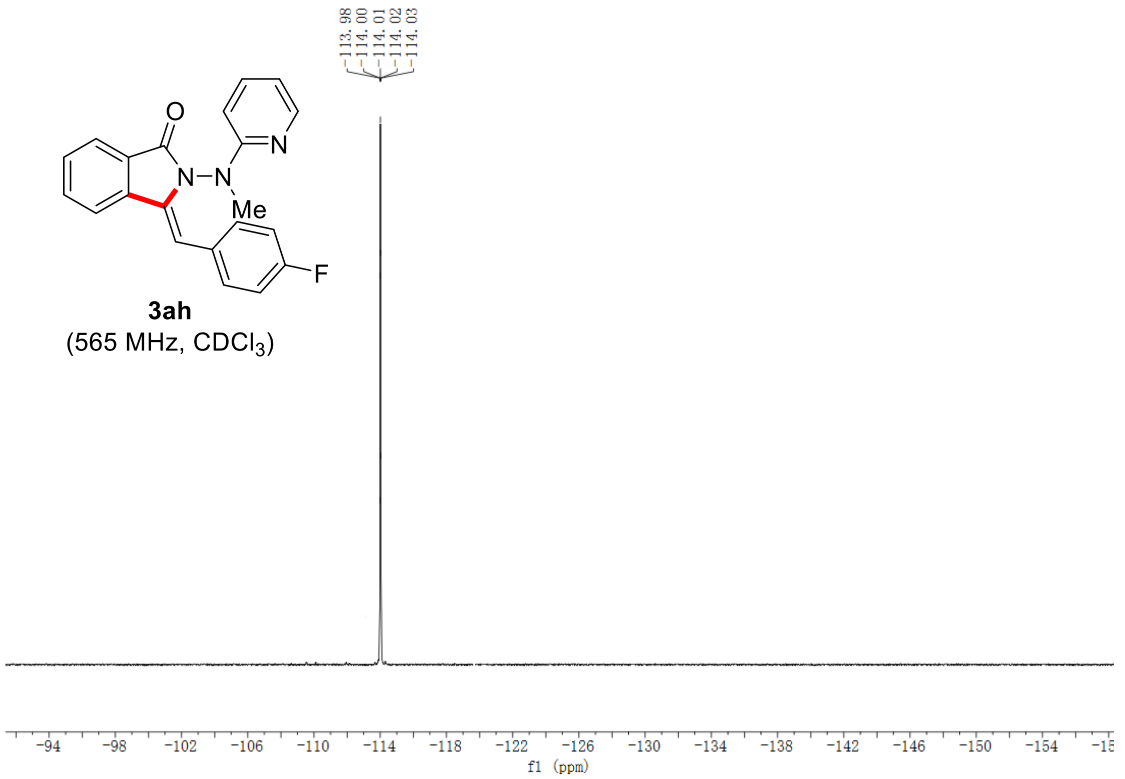

3ah
(565 MHz, $CDCl_3$)

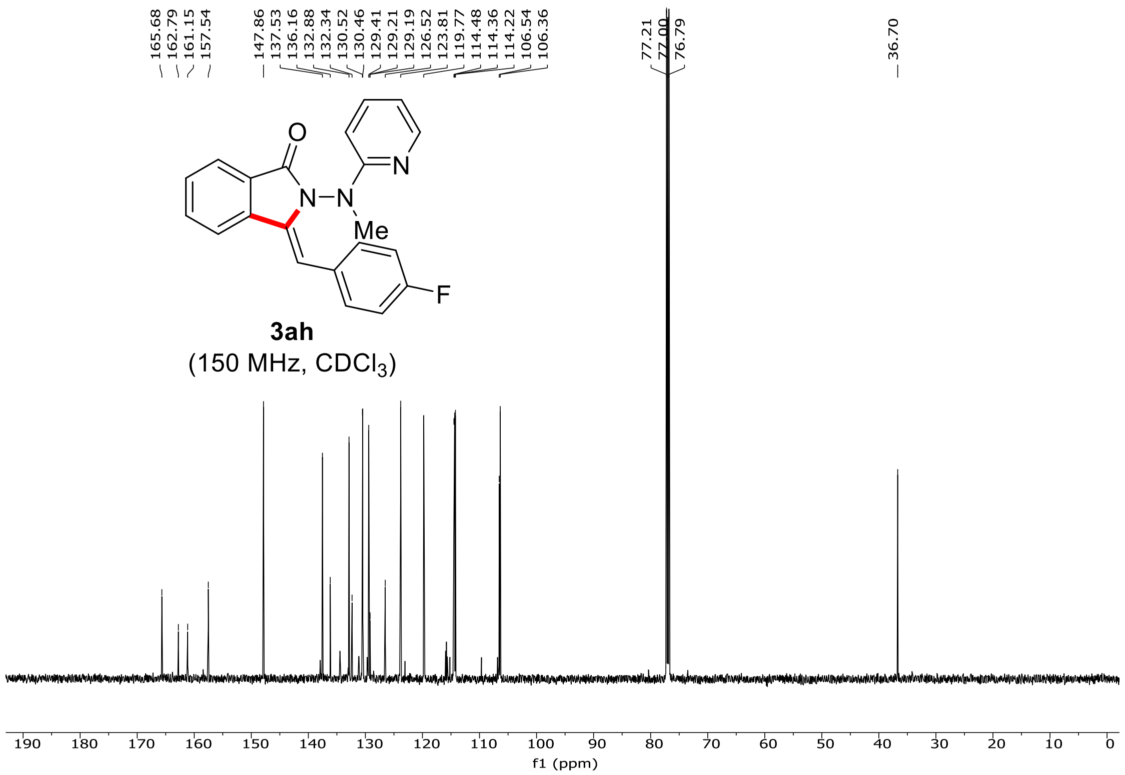
165.68
162.79
161.15
157.54
147.86
137.53
136.16
132.88
132.34
130.52
130.46
129.41
129.21
129.19
126.52
123.81
119.77
114.48
114.36
114.22
106.54
106.36
77.21
77.00
76.79
36.70
O
N
N–N
Me
F
3ah
(150 MHz, $CDCl_3$)
190 180 170 160 150 140 130 120 110 100 90 80 70 60 50 40 30 20 10 0
f1 (ppm)

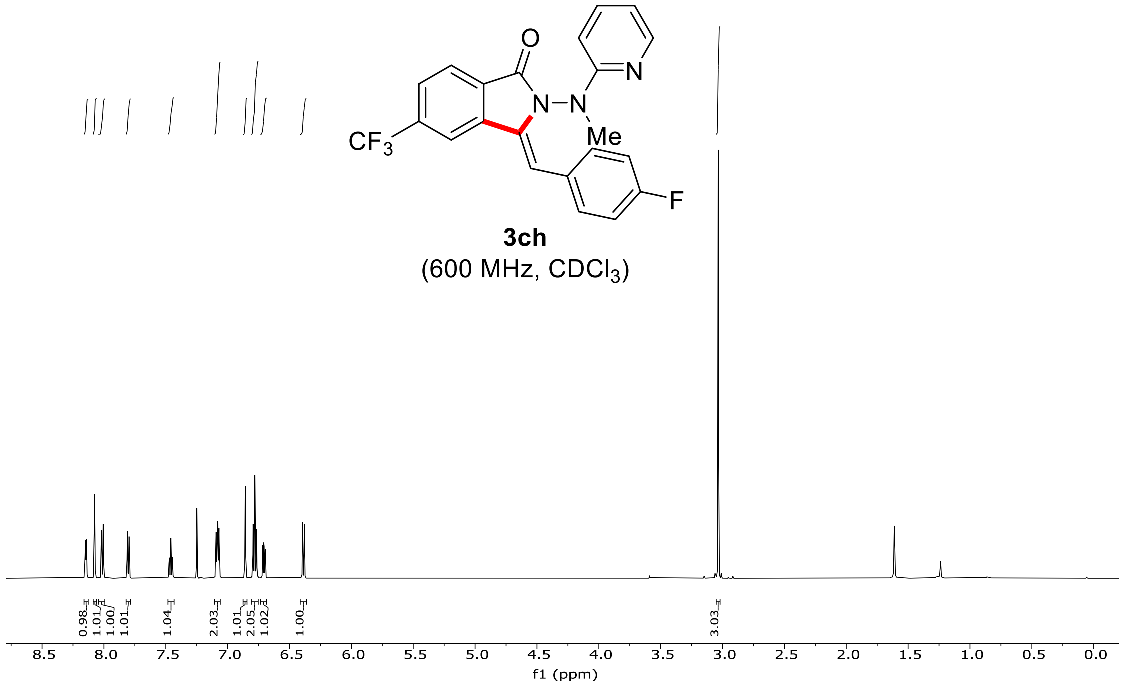
O
N
N–N
Me
CF_3
F
3ch
(600 MHz, $CDCl_3$)
0.98
1.01
1.00
1.01
1.04
2.03
1.01
2.05
1.02
1.00
3.03
8.5 8.0 7.5 7.0 6.5 6.0 5.5 5.0 4.5 4.0 3.5 3.0 2.5 2.0 1.5 1.0 0.5 0.0
f1 (ppm)

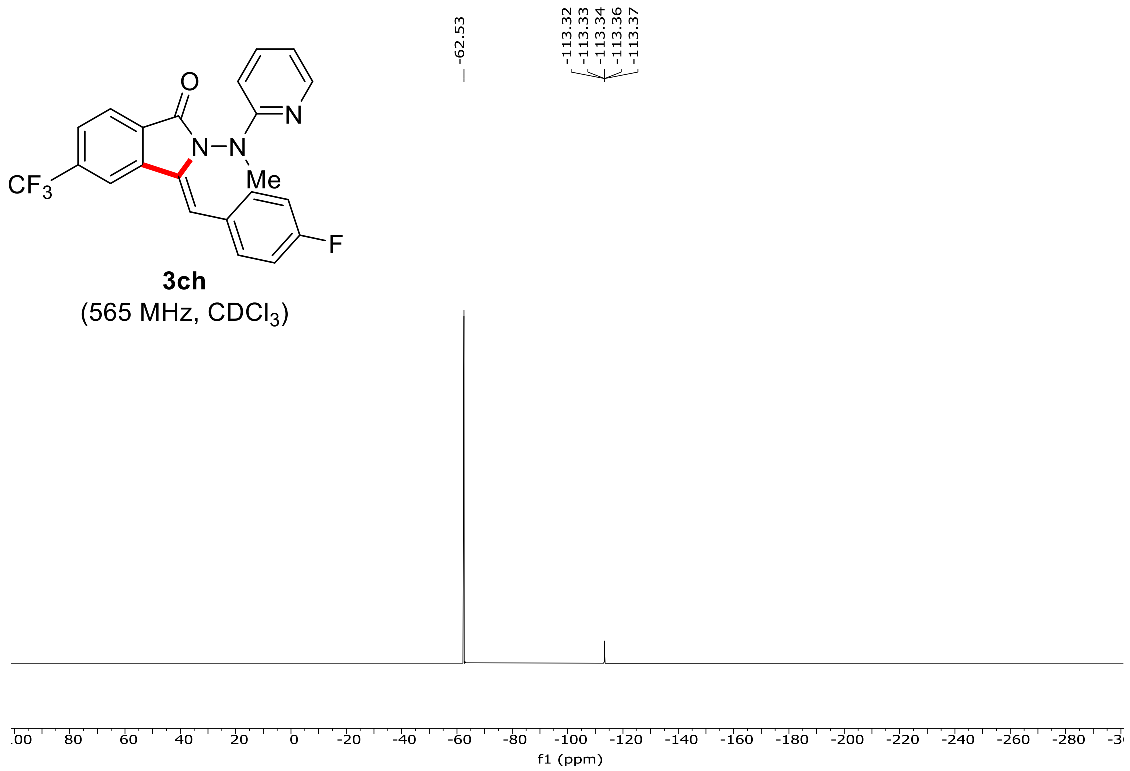
-62.53
-113.32
-113.33
-113.34
-113.36
-113.37
CF3
O
N
N
Me
F
3ch
(565 MHz, CDCl3)
f1 (ppm)

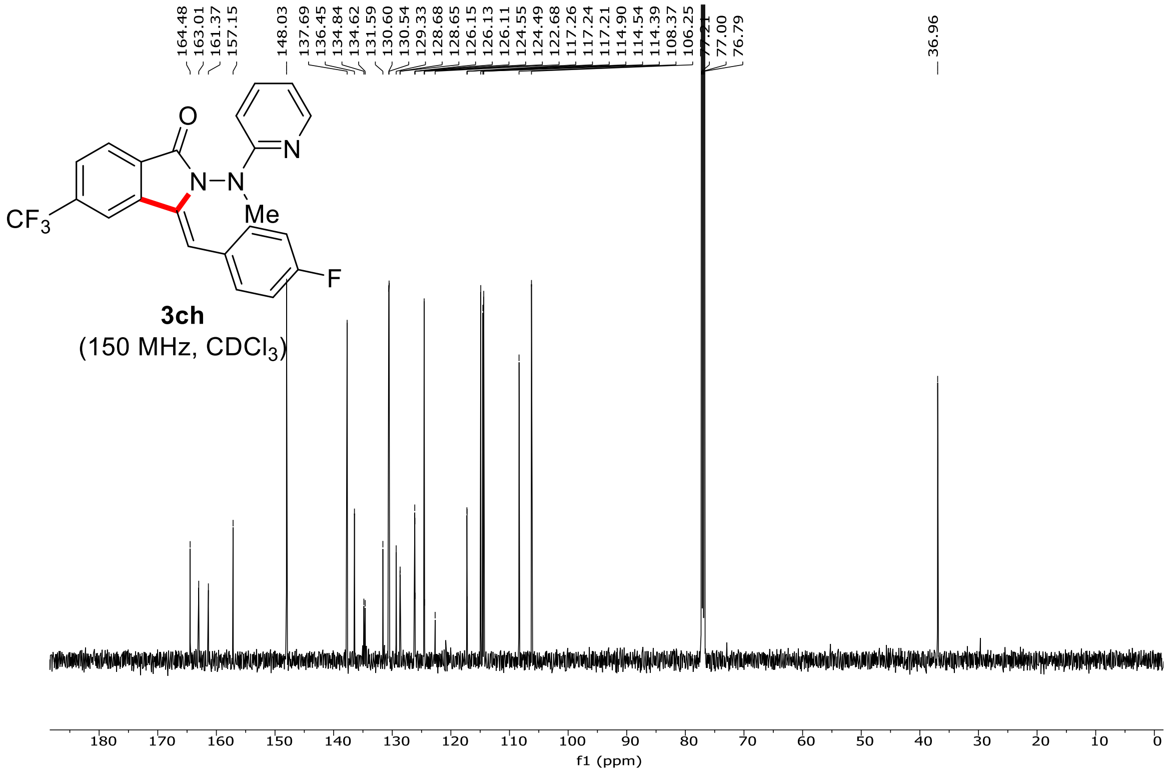
164.48
163.01
161.37
157.15
148.03
137.69
136.45
134.84
134.62
131.59
130.60
130.54
129.33
128.68
128.65
126.15
126.13
126.11
124.55
124.49
122.68
117.26
117.24
117.21
114.90
114.54
114.39
108.37
106.25
77.21
77.00
76.79
36.96
CF3
O
N
N
Me
F
3ch
(150 MHz, CDCl3)
f1 (ppm)

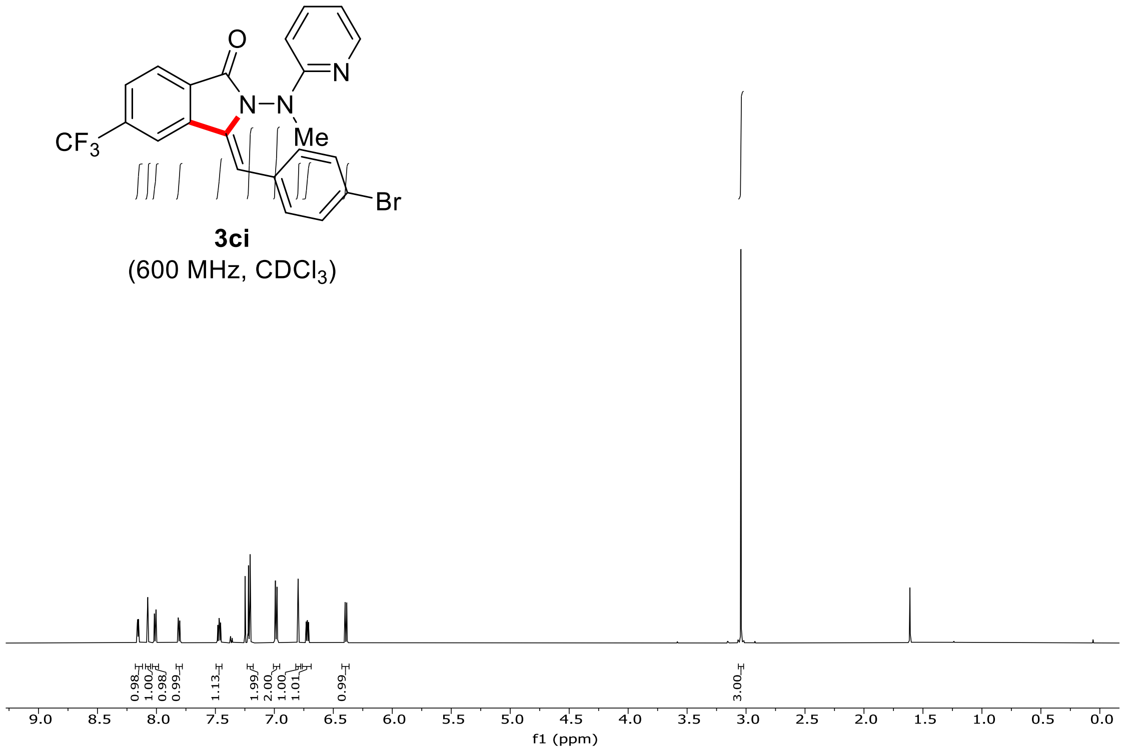
3ci
(600 MHz, CDCl3)
f1 (ppm)

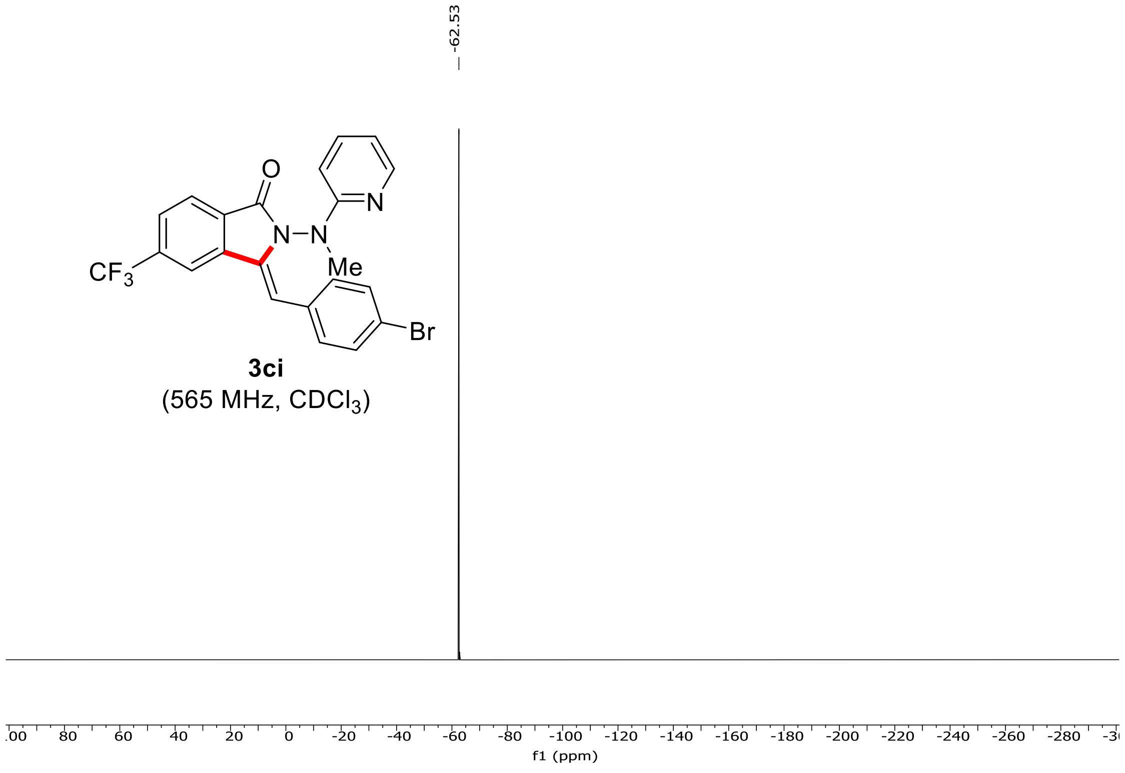
-62.53
3ci
(565 MHz, CDCl3)
f1 (ppm)

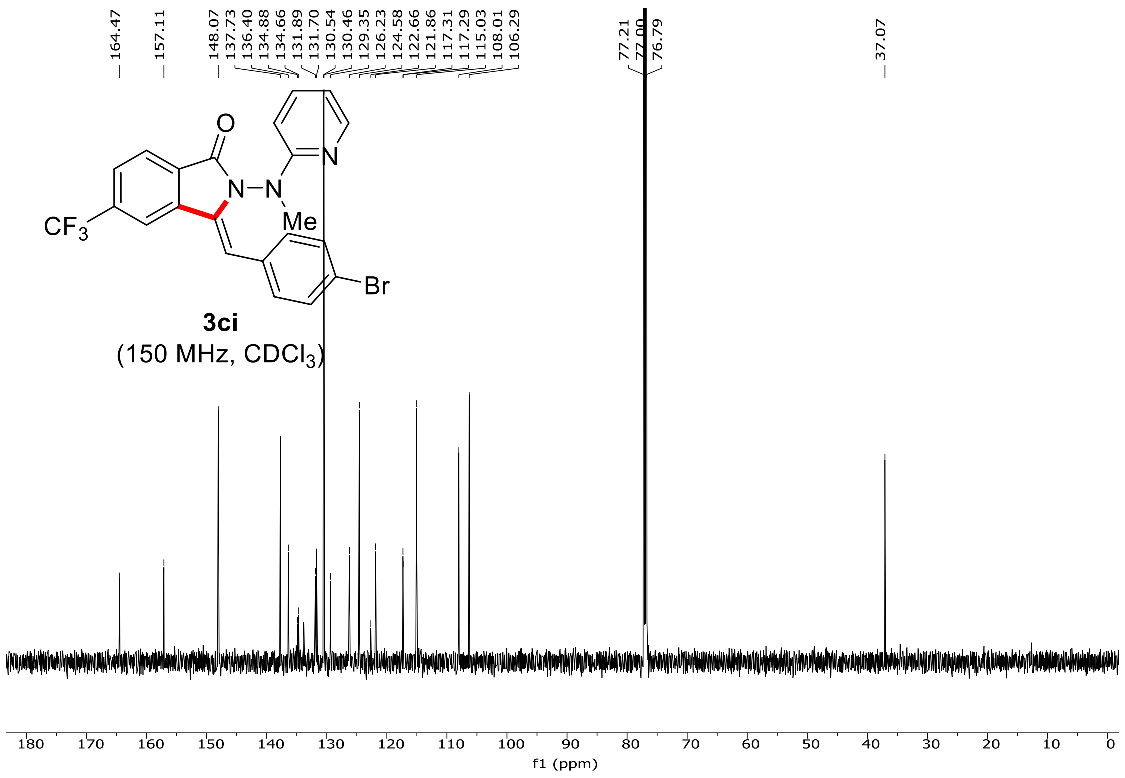

3ci
(150 MHz, $CDCl_3$)

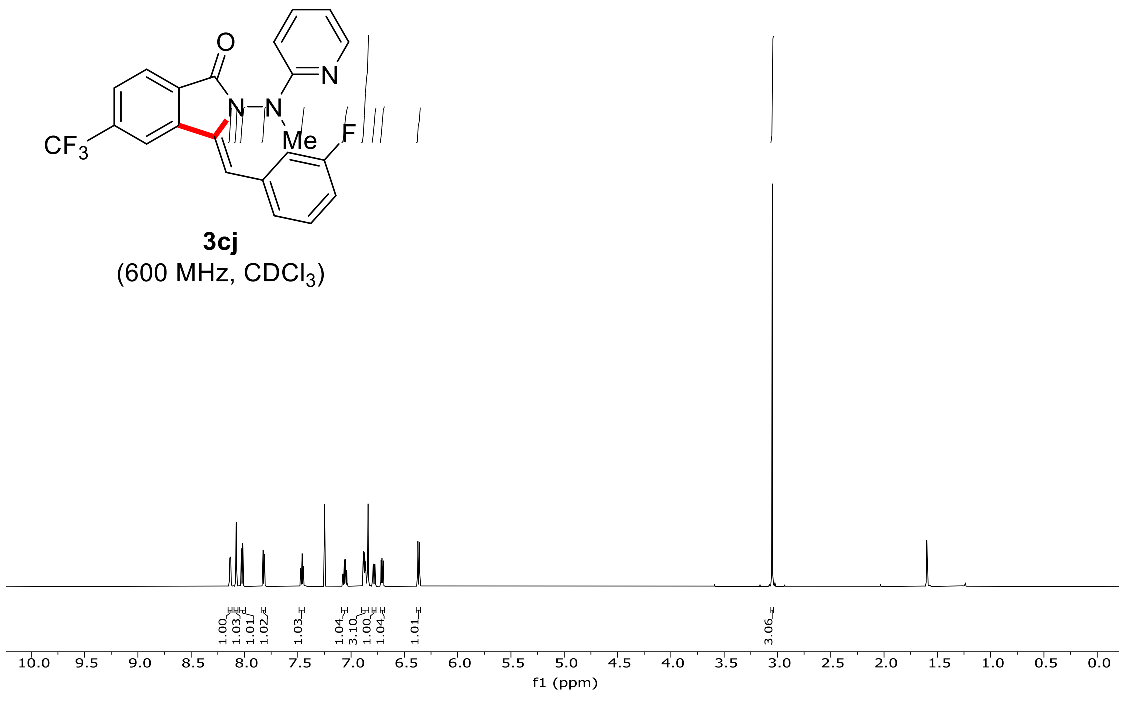

3cj
(600 MHz, $CDCl_3$)

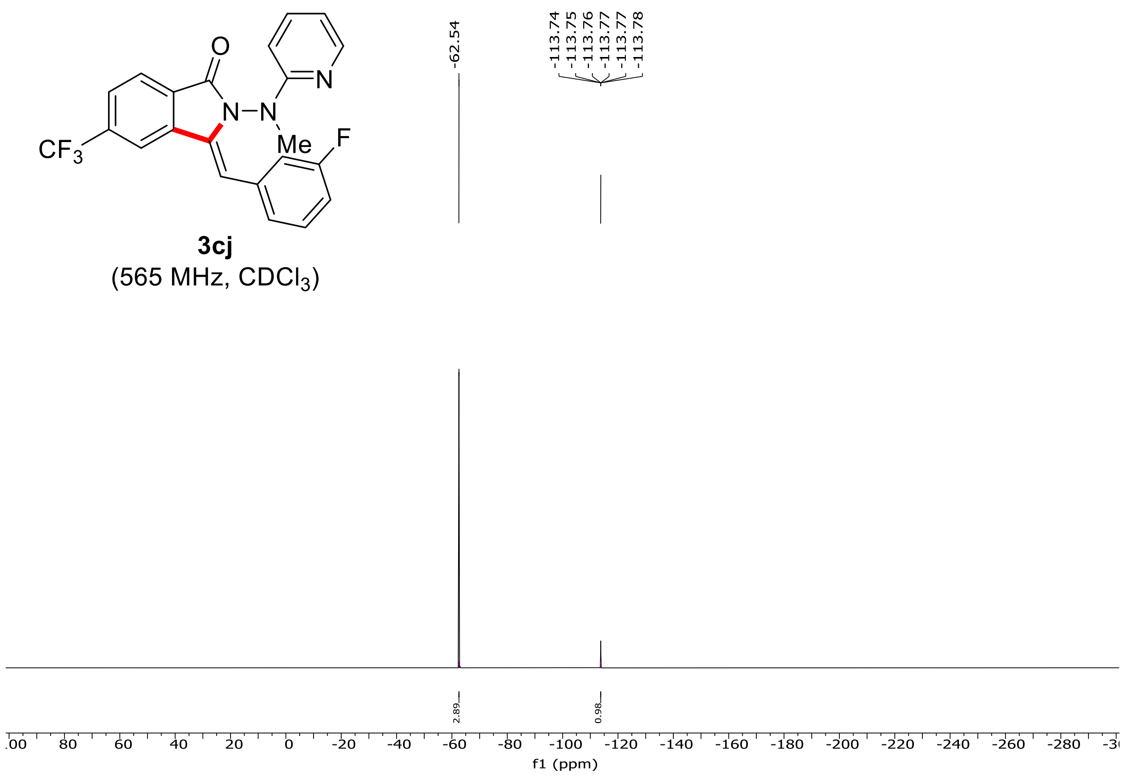

3cj
(565 MHz, CDCl3)
-62.54
-113.74
-113.75
-113.76
-113.77
-113.77
-113.78
2.89
0.98
f1 (ppm)

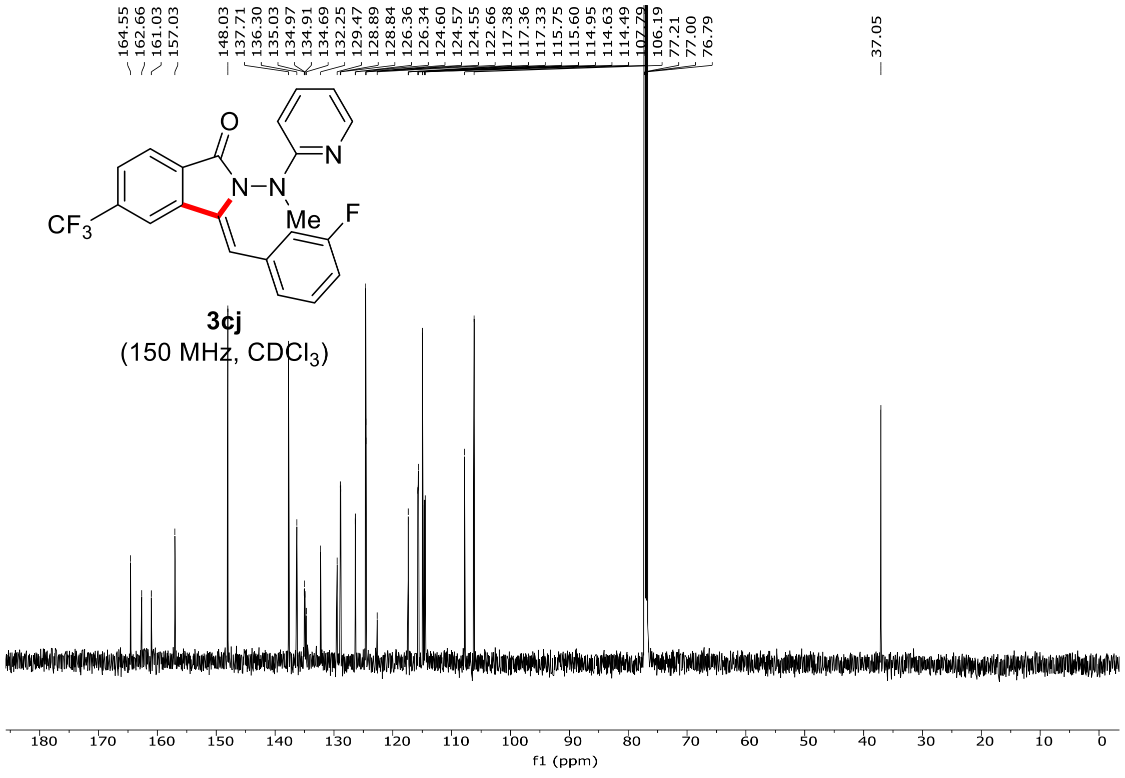

3cj
(150 MHz, CDCl3)
164.55
162.66
161.03
157.03
148.03
137.71
136.30
135.03
134.97
134.91
134.69
132.25
129.47
128.89
128.84
126.36
126.34
124.60
124.57
124.55
122.66
117.38
117.36
117.33
115.75
115.60
114.95
114.63
114.49
107.79
106.19
77.21
77.00
76.79
37.05
f1 (ppm)

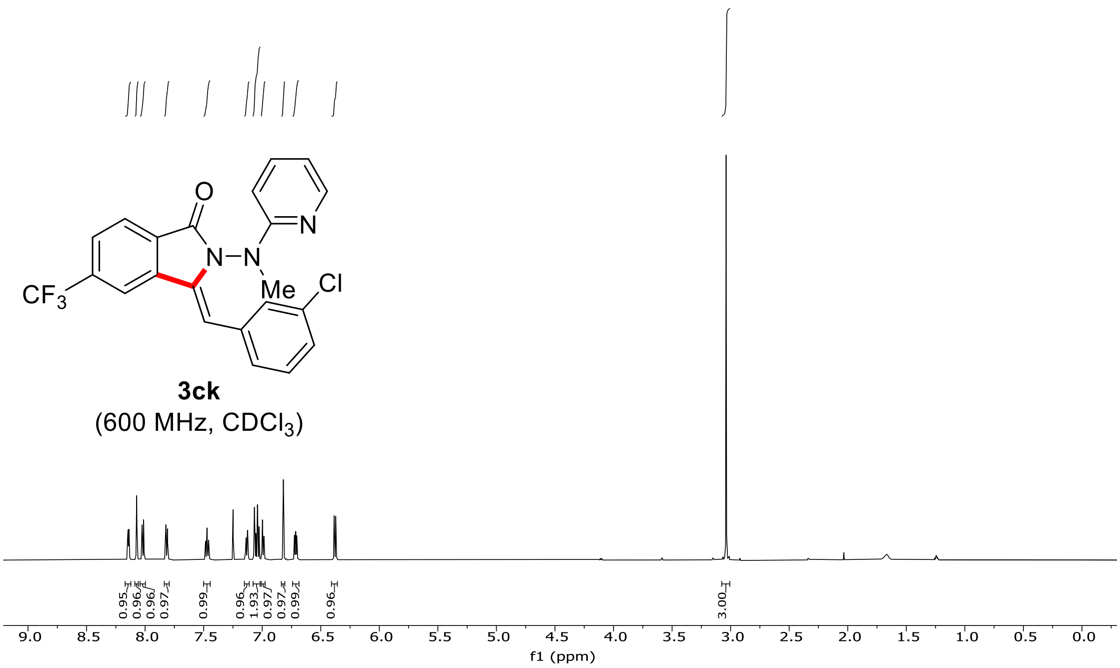
3ck
(600 MHz, $CDCl_3$)
0.95
0.96
0.96
0.97
0.99
0.96
1.93
0.97
0.97
0.99
0.96
3.00
9.0 8.5 8.0 7.5 7.0 6.5 6.0 5.5 5.0 4.5 4.0 3.5 3.0 2.5 2.0 1.5 1.0 0.5 0.0
f1 (ppm)

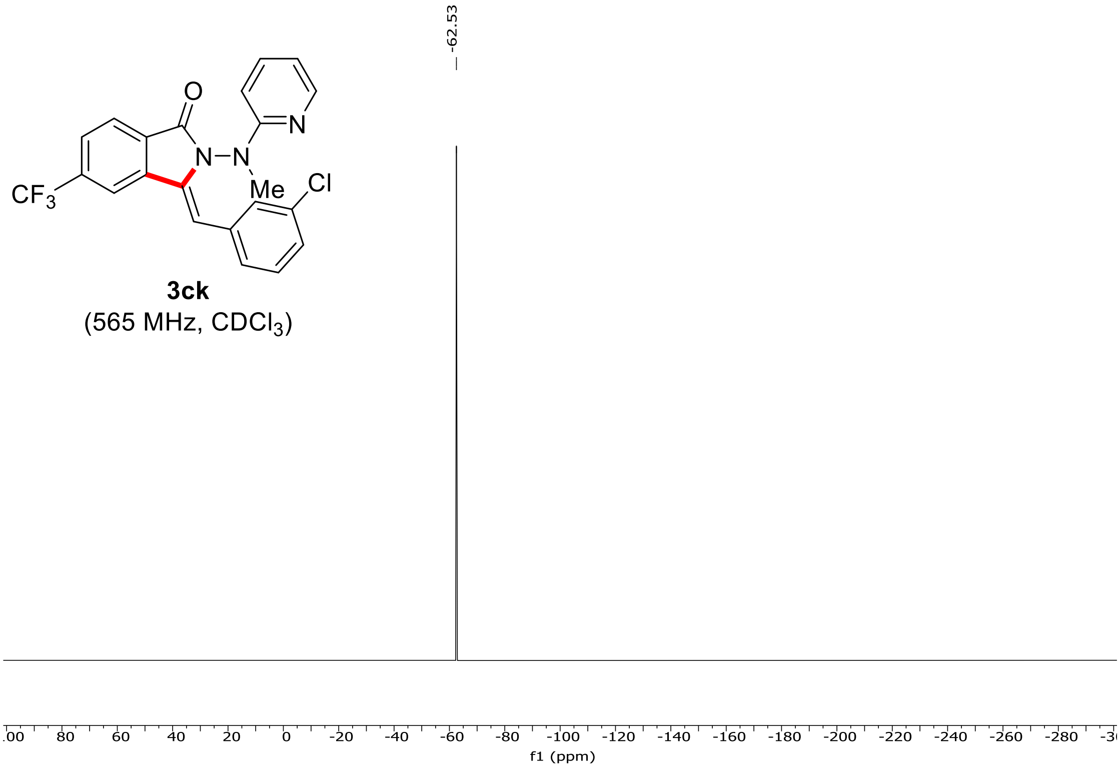
-62.53
3ck
(565 MHz, $CDCl_3$)
80 60 40 20 0 -20 -40 -60 -80 -100 -120 -140 -160 -180 -200 -220 -240 -260 -280
f1 (ppm)

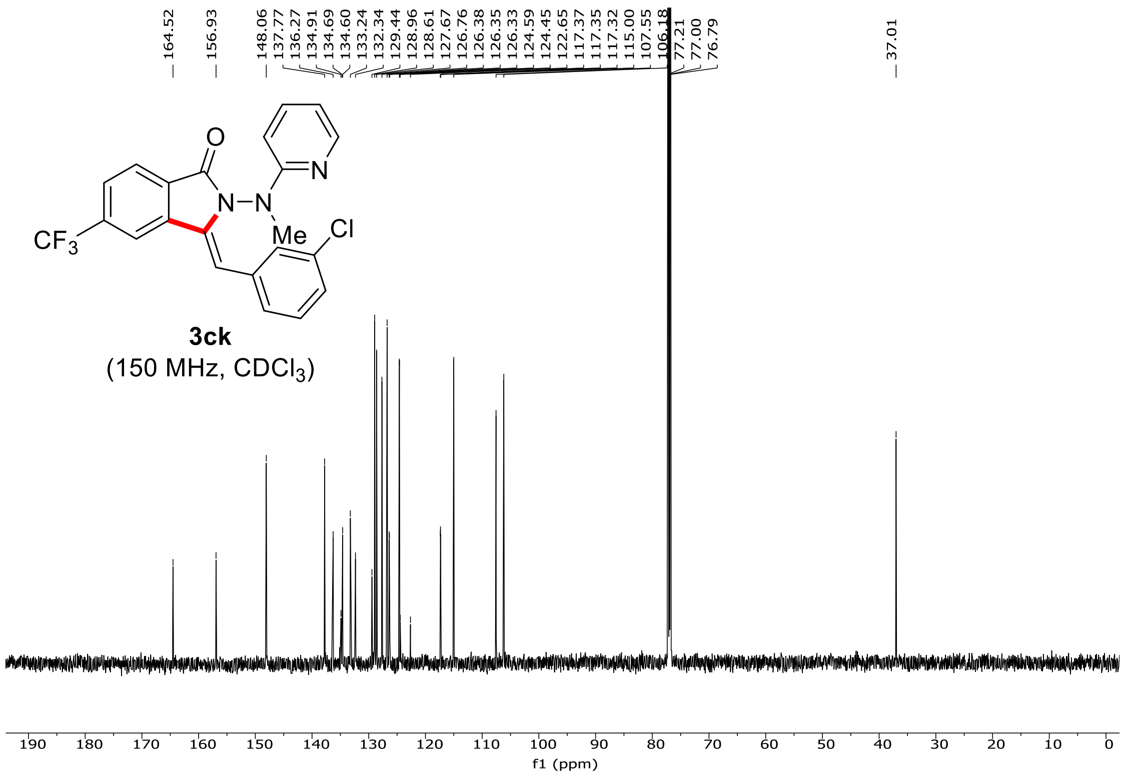
164.52
156.93
148.06
137.77
136.27
134.91
134.69
134.60
133.24
132.34
129.44
128.96
128.61
127.67
126.76
126.38
126.35
126.33
124.59
124.45
122.65
117.37
117.35
117.32
115.00
107.55
106.18
77.21
77.00
76.79
37.01
O
N
N–N
Me
Cl
CF3
3ck
(150 MHz, CDCl3)
f1 (ppm)

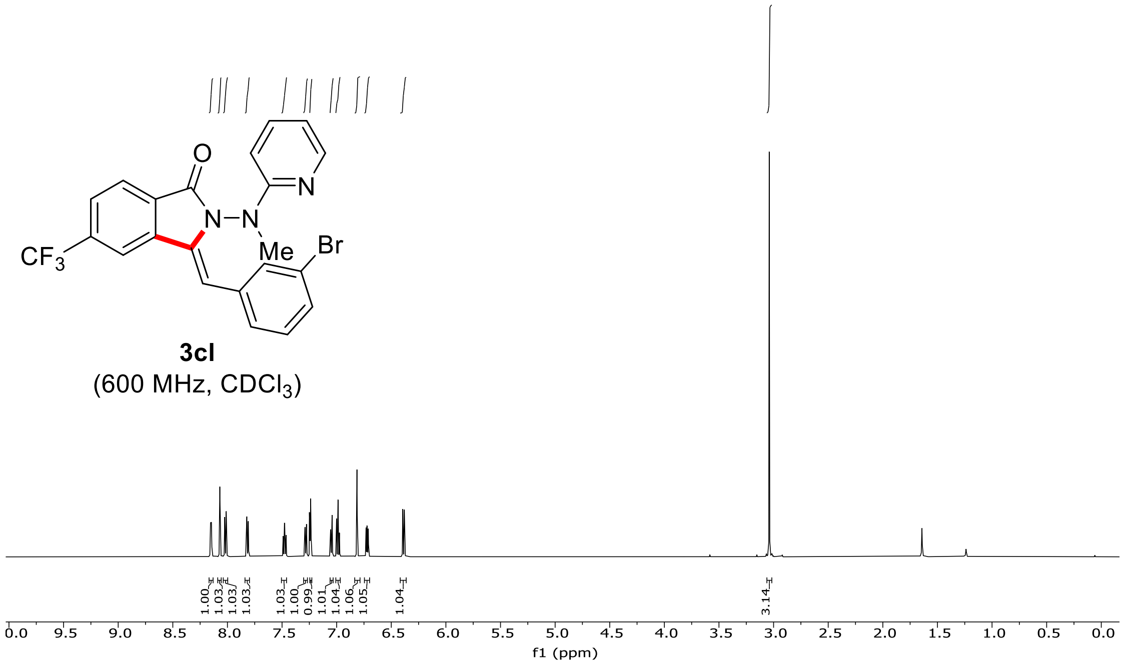
O
N
N–N
Me
Br
CF3
3cl
(600 MHz, CDCl3)
1.00
1.03
1.03
1.03
1.03
1.00
0.99
1.01
1.04
1.06
1.05
1.04
3.14
f1 (ppm)

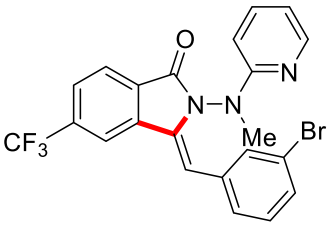

3cl

(565 MHz, $CDCl_3$)

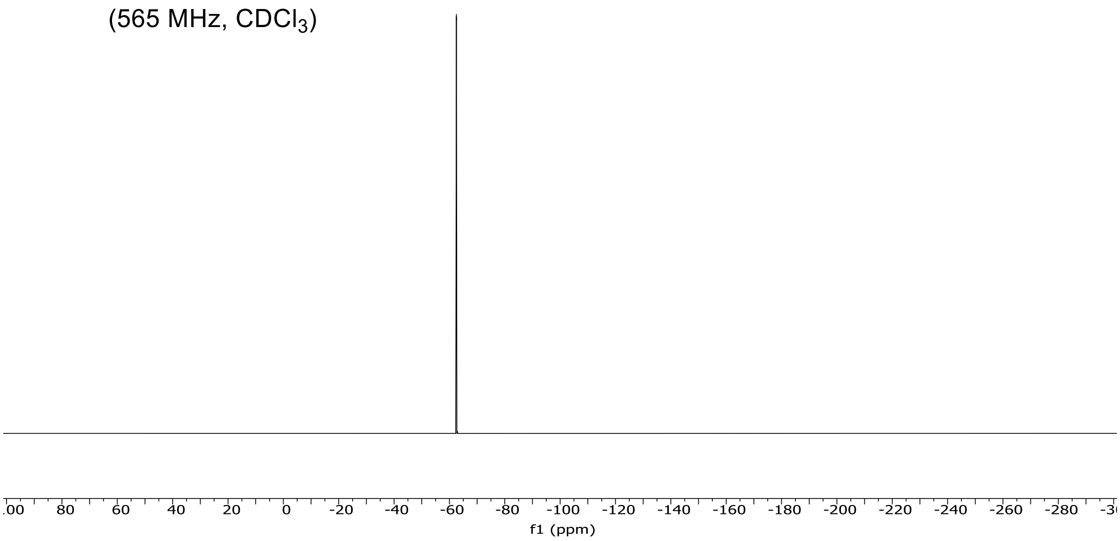

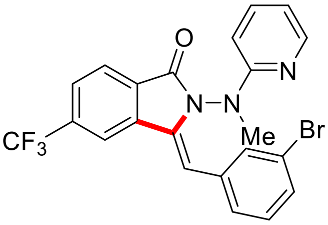

3cl

(150 MHz, $CDCl_3$)

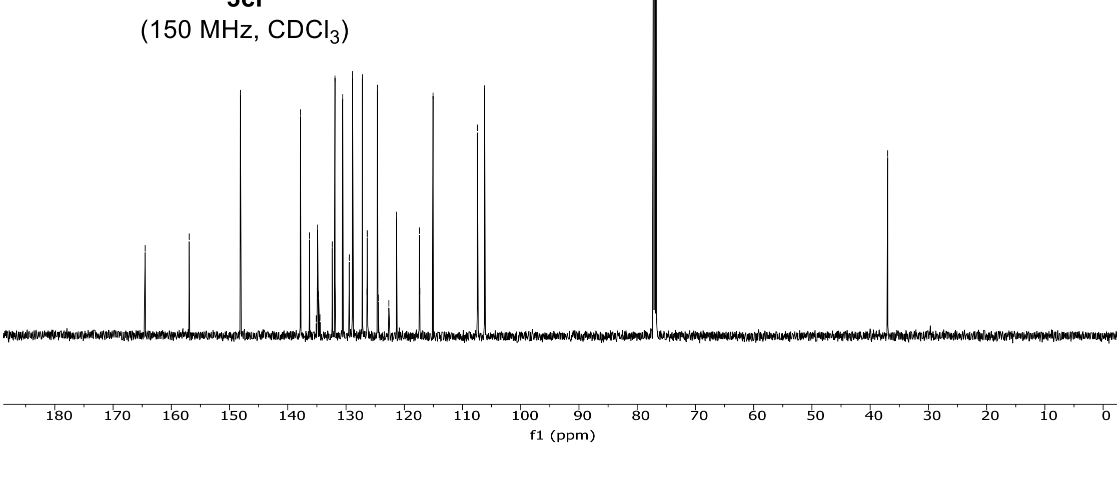

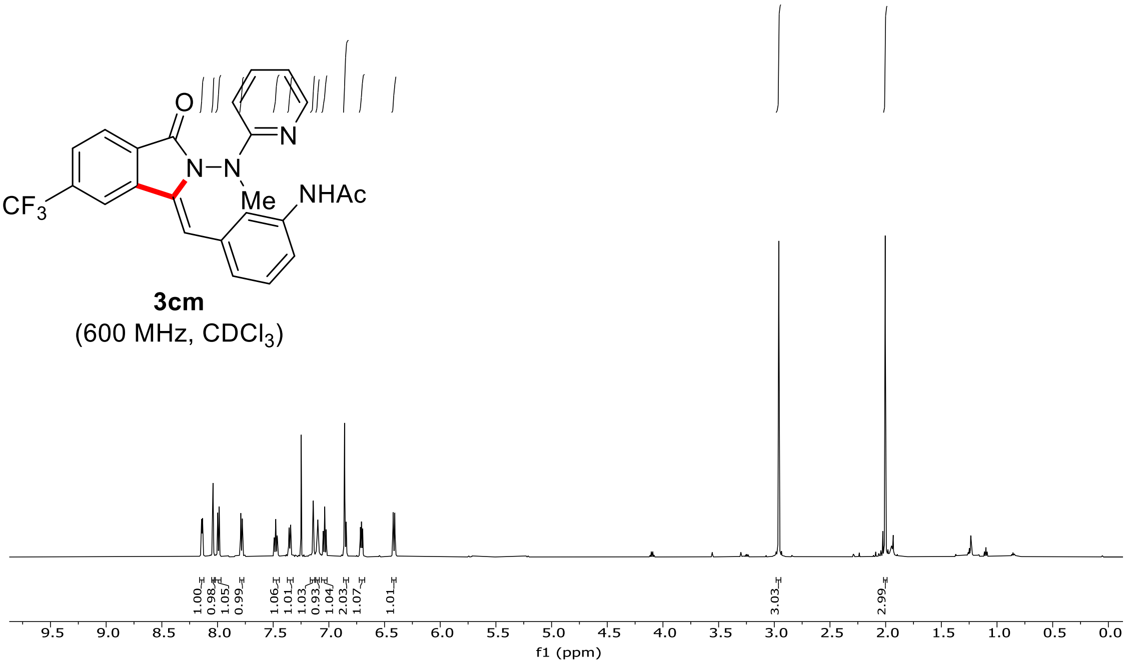
O
N
N–N
Me
NHAc
CF3
3cm
(600 MHz, CDCl3)
1.00
0.98
1.05
0.99
1.06
1.01
1.03
0.93
1.04
2.03
1.07
1.01
3.03
2.99
9.5 9.0 8.5 8.0 7.5 7.0 6.5 6.0 5.5 5.0 4.5 4.0 3.5 3.0 2.5 2.0 1.5 1.0 0.5 0.0
f1 (ppm)

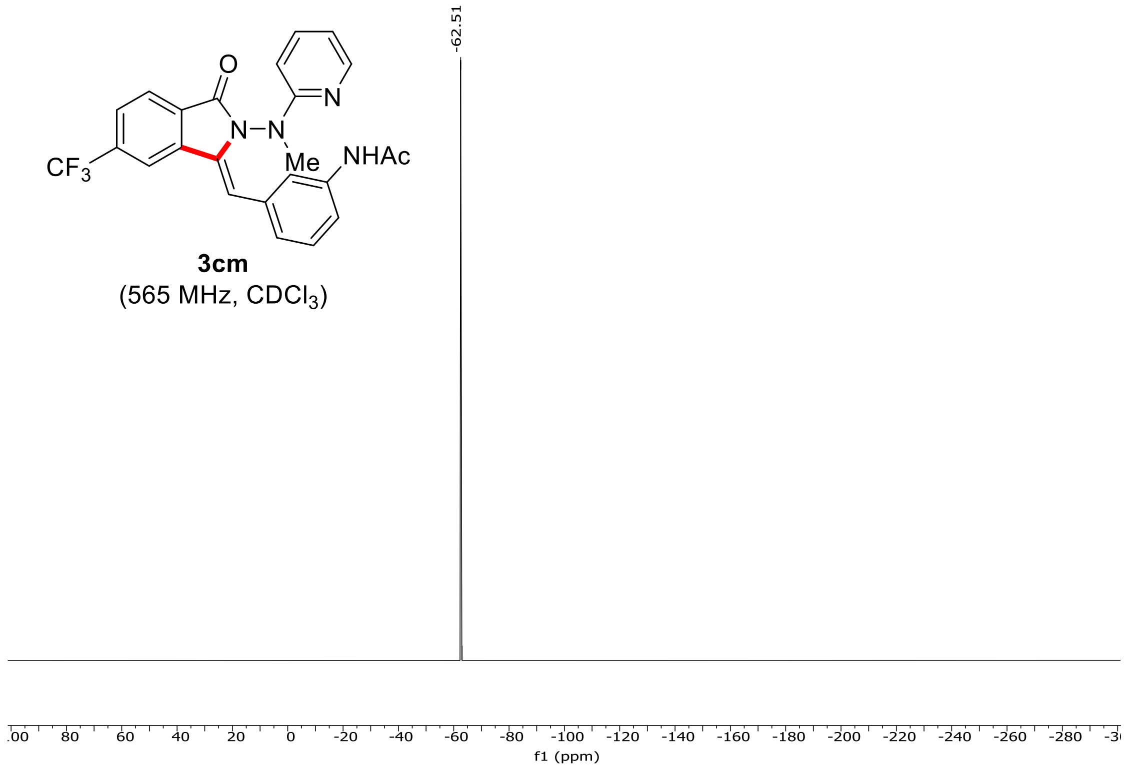
-62.51
O
N
N–N
Me
NHAc
CF3
3cm
(565 MHz, CDCl3)
80 60 40 20 0 -20 -40 -60 -80 -100 -120 -140 -160 -180 -200 -220 -240 -260 -280
f1 (ppm)

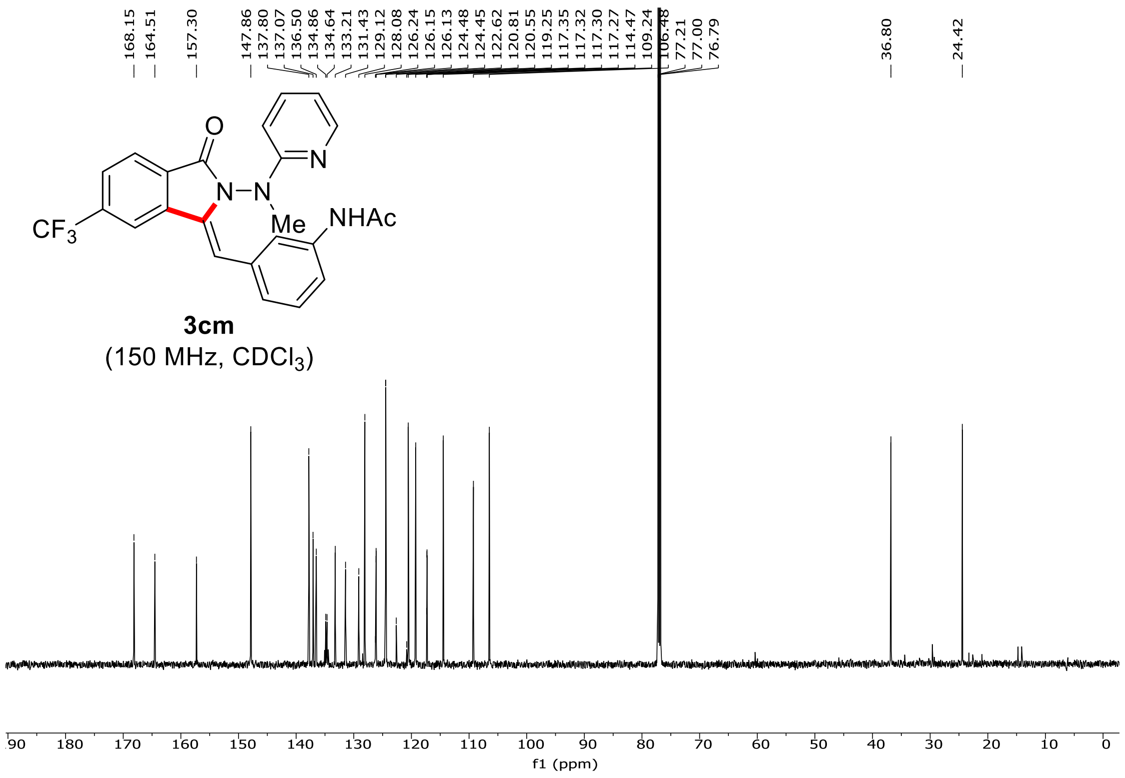
168.15
164.51
157.30
147.86
137.80
137.07
136.50
134.86
134.64
133.21
131.43
129.12
128.08
126.24
126.15
126.13
124.48
124.45
122.62
120.81
120.55
119.25
117.35
117.32
117.30
117.27
114.47
109.24
106.48
77.21
77.00
76.79
36.80
24.42
O
N
N–N
Me
NHAc
CF3
3cm
(150 MHz, CDCl3)
f1 (ppm)

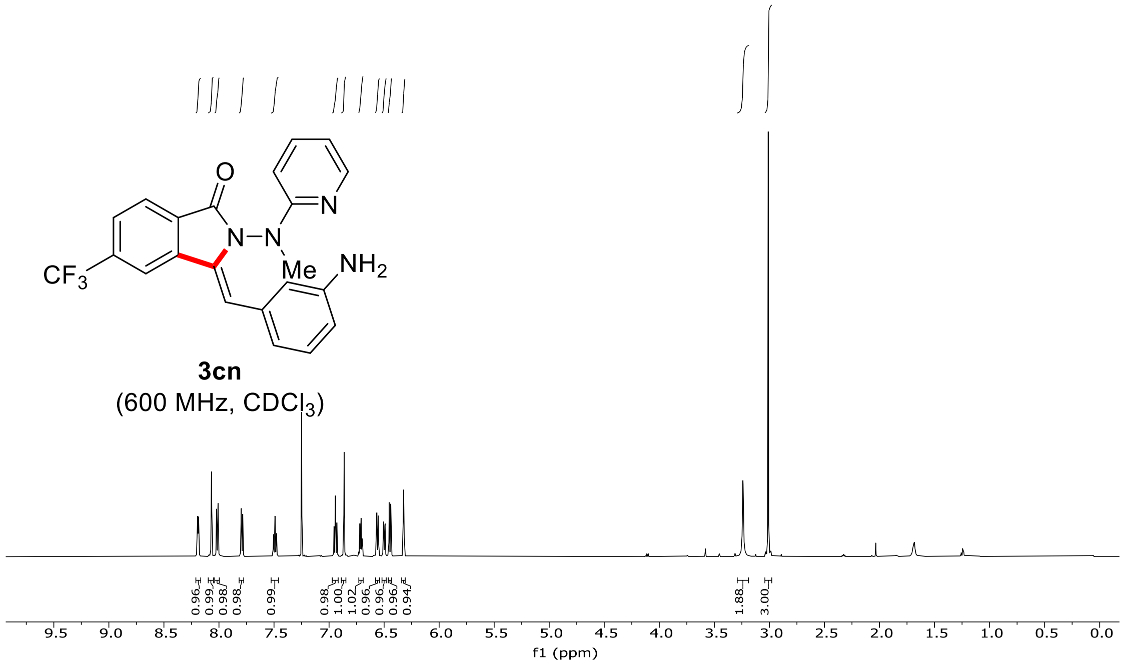
O
N
N–N
Me
NH2
CF3
3cn
(600 MHz, CDCl3)
0.96
0.99
0.98
0.98
0.99
0.98
1.00
1.02
0.96
0.96
0.96
0.94
1.88
3.00
f1 (ppm)

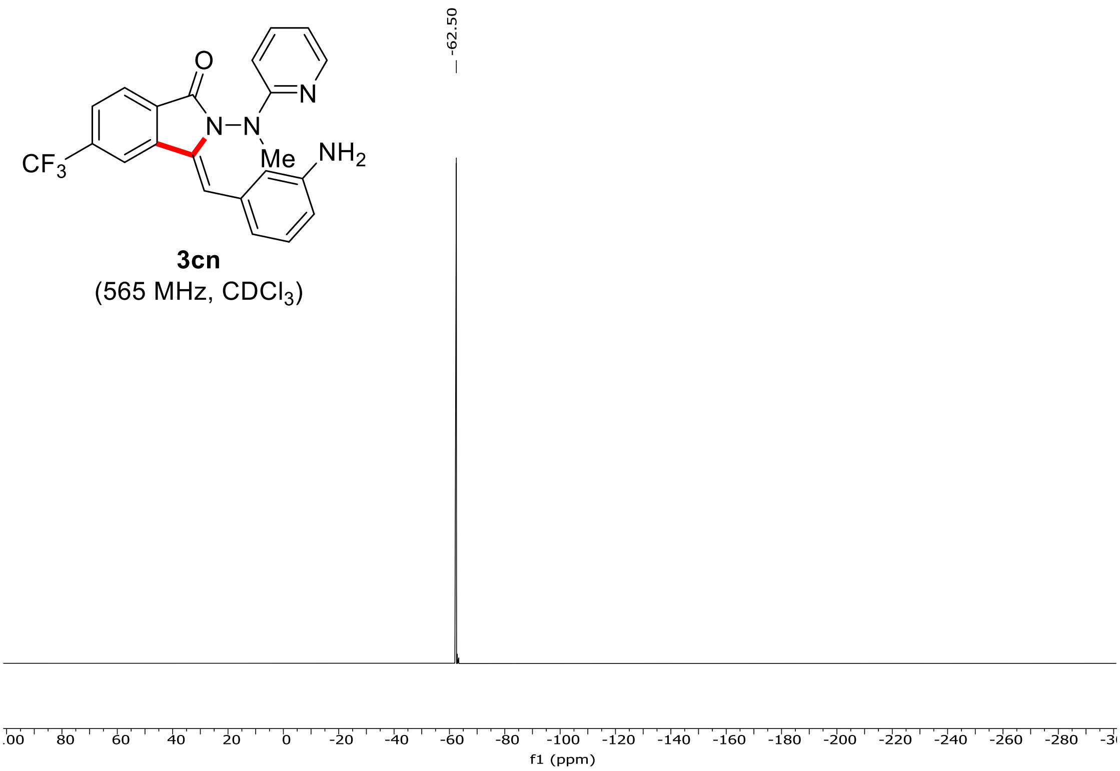
-62.50
CF3
NH2
Me
3cn
(565 MHz, CDCl3)
f1 (ppm)

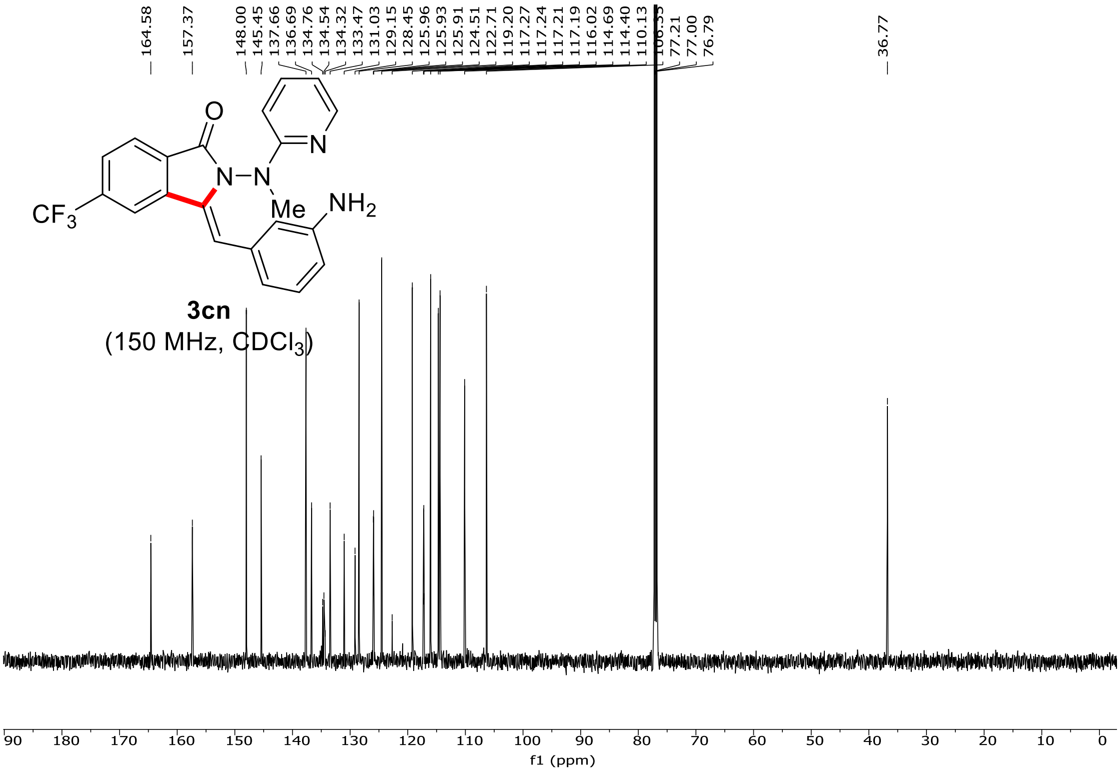
164.58
157.37
148.00
145.45
137.66
136.69
134.76
134.54
134.32
133.47
131.03
129.15
128.45
125.96
125.93
125.91
124.51
122.71
119.20
117.27
117.24
117.21
117.19
116.02
114.69
114.40
110.13
77.21
77.00
76.79
36.77
CF3
NH2
Me
3cn
(150 MHz, CDCl3)
f1 (ppm)

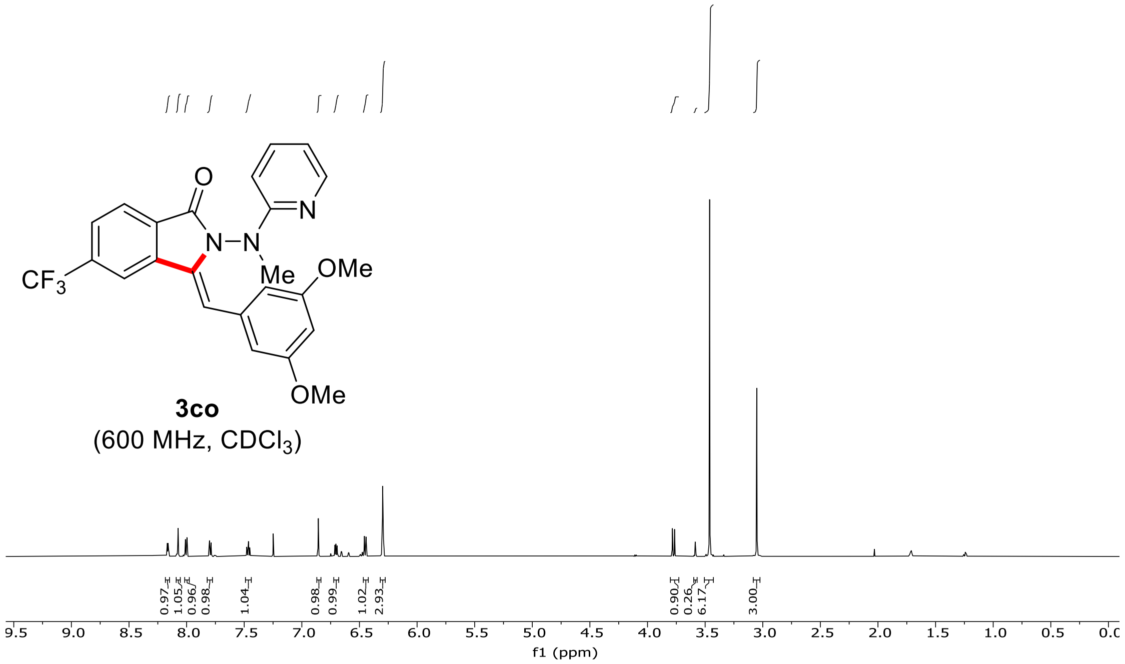

O
N
N–N
Me
OMe
CF3
OMe
3co
(600 MHz, CDCl3)
0.97
1.05
0.96
0.98
1.04
0.98
0.99
1.02
2.93
0.90
0.26
6.17
3.00
9.5
9.0
8.5
8.0
7.5
7.0
6.5
6.0
5.5
5.0
4.5
4.0
3.5
3.0
2.5
2.0
1.5
1.0
0.5
f1 (ppm)

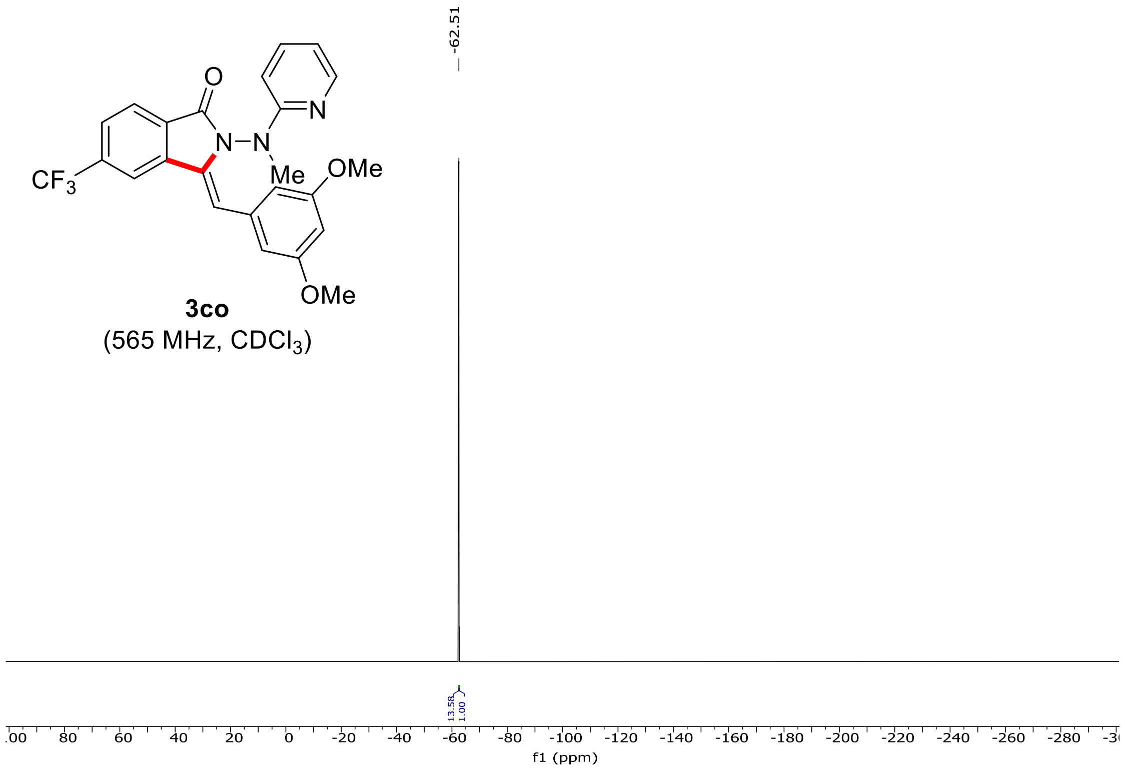

–62.51
O
N
N–N
Me
OMe
CF3
OMe
3co
(565 MHz, CDCl3)
13.58
1.00
80
60
40
20
0
-20
-40
-60
-80
-100
-120
-140
-160
-180
-200
-220
-240
-260
-280
f1 (ppm)

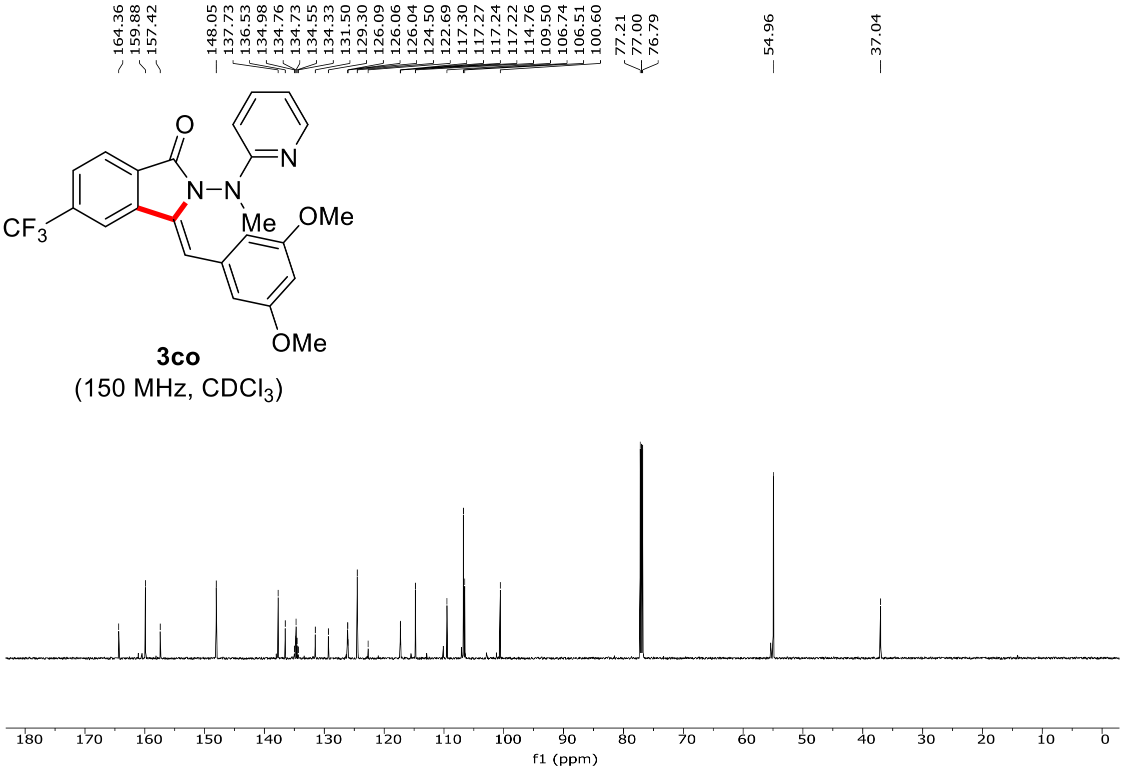

3co
(150 MHz, $CDCl_3$)

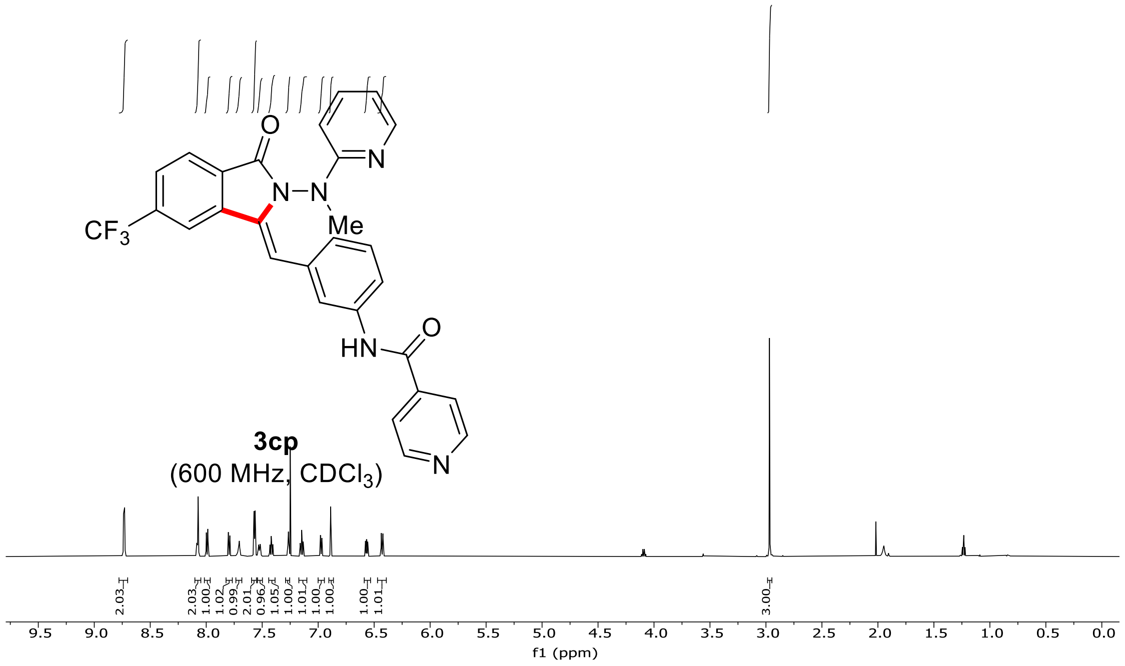

3cp
(600 MHz, $CDCl_3$)

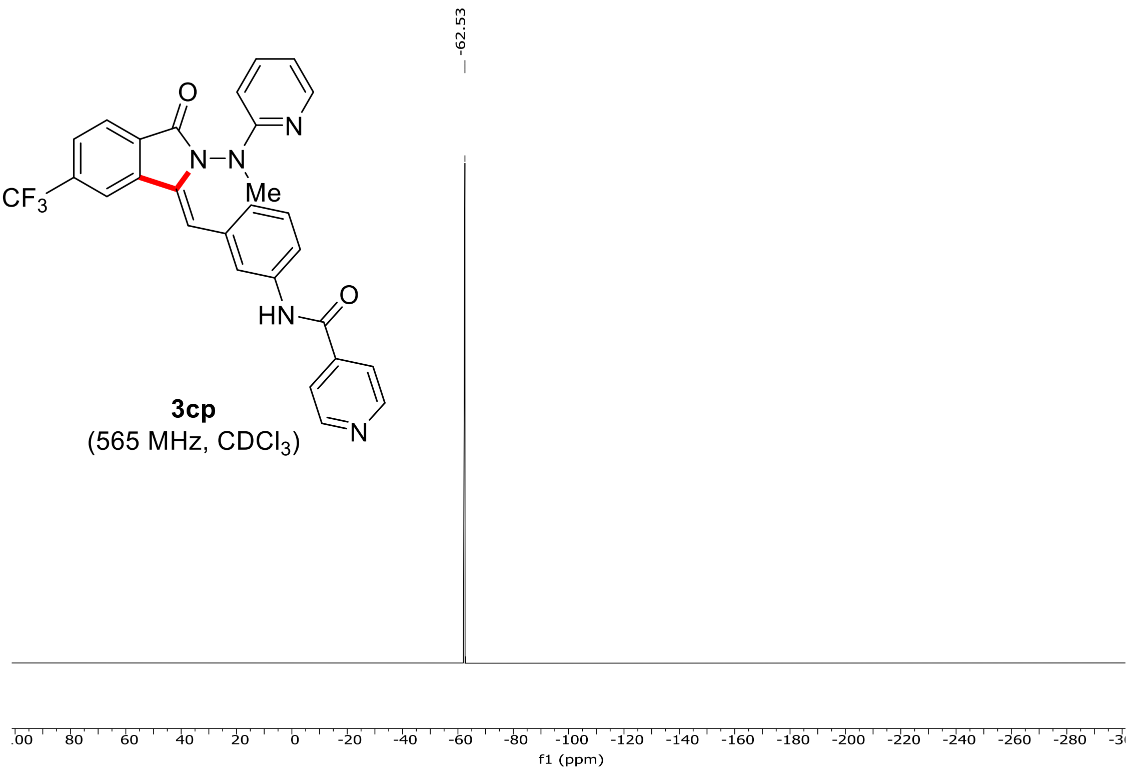
-62.53
O
N
N–N
Me
CF3
HN
O
N
3cp
(565 MHz, CDCl3)
f1 (ppm)

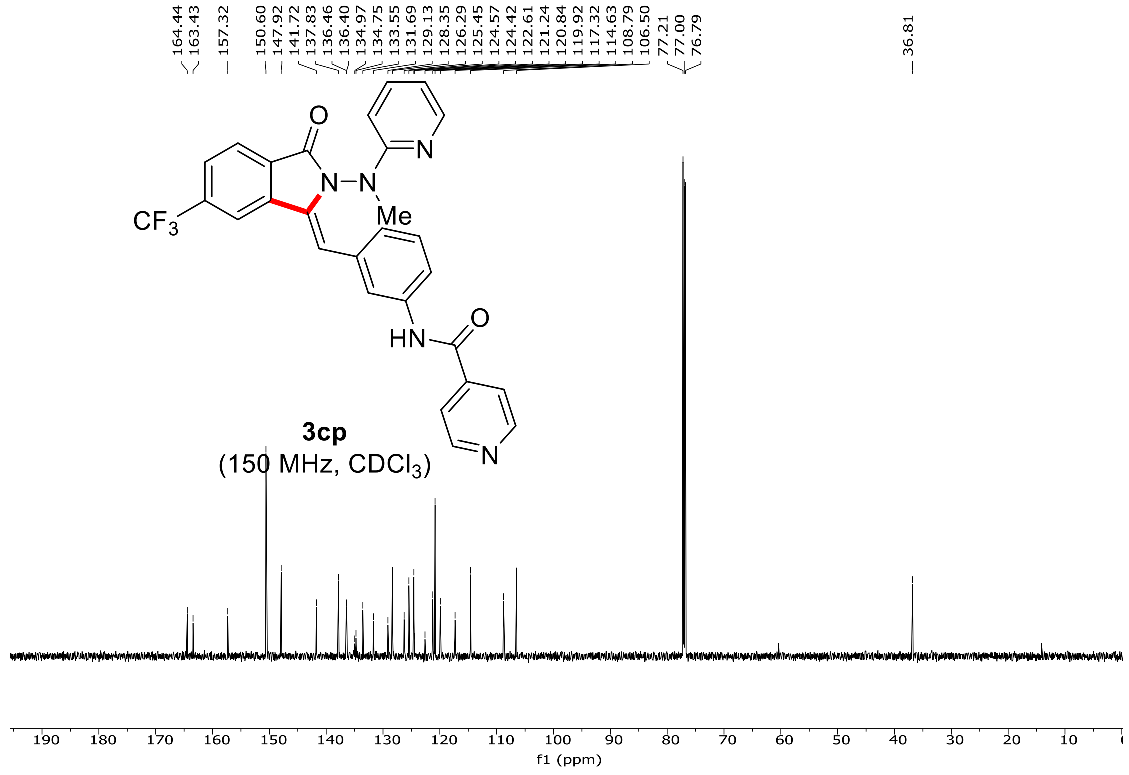
164.44
163.43
157.32
150.60
147.92
141.72
137.83
136.46
136.40
134.97
134.75
133.55
131.69
129.13
128.35
126.29
125.45
124.57
124.42
122.61
121.24
120.84
119.92
117.32
114.63
108.79
106.50
77.21
77.00
76.79
36.81
O
N
N–N
Me
CF3
HN
O
N
3cp
(150 MHz, CDCl3)
f1 (ppm)

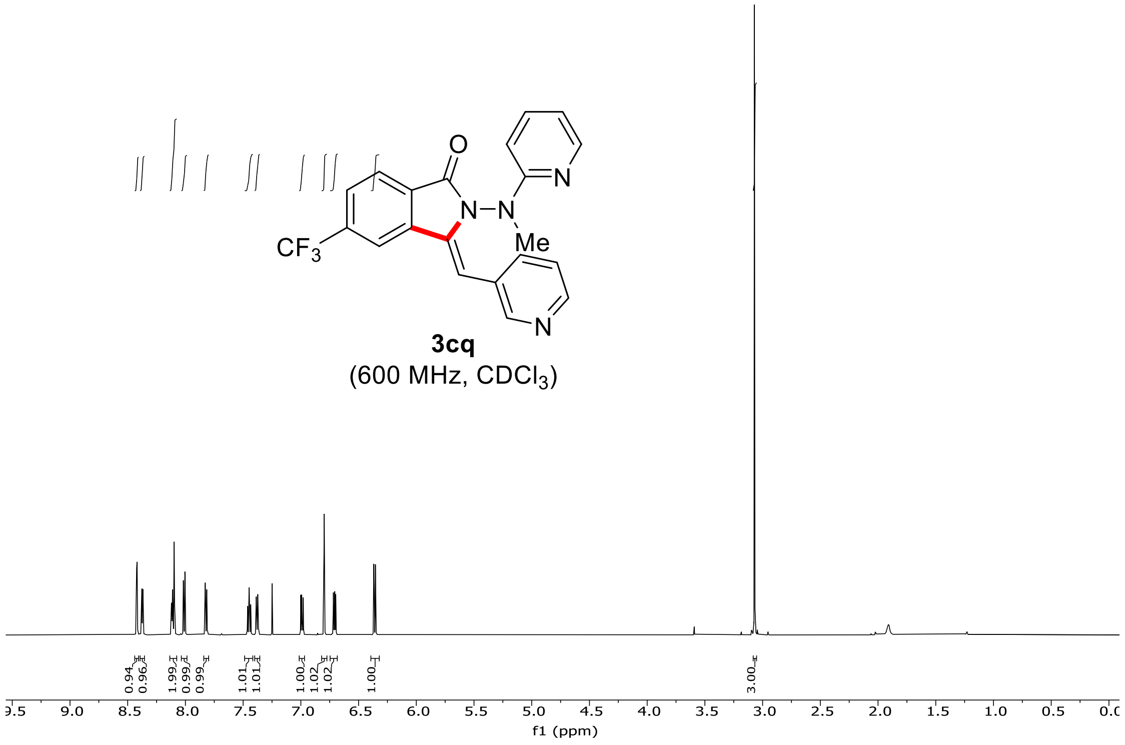
O
N
N–N
Me
CF3
3cq
(600 MHz, CDCl3)
0.94
0.96
1.99
0.99
0.99
1.01
1.01
1.00
1.02
1.02
1.00
3.00
f1 (ppm)

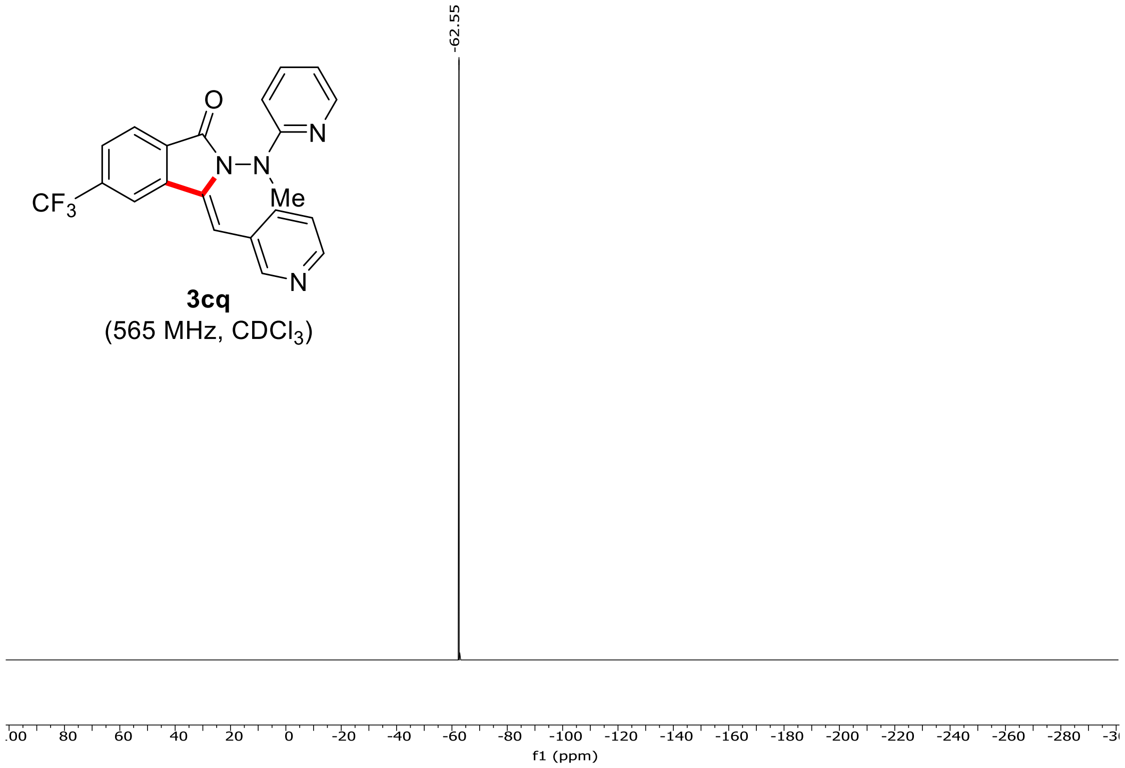
-62.55
O
N
N–N
Me
CF3
3cq
(565 MHz, CDCl3)
f1 (ppm)

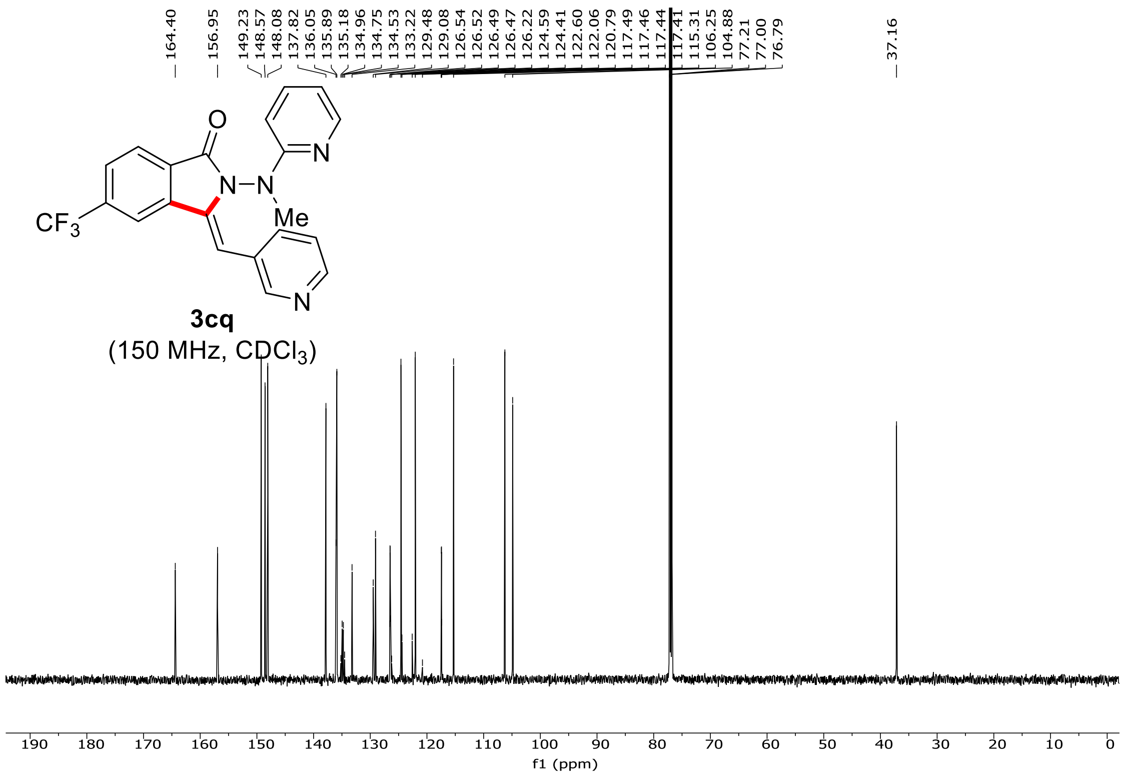
164.40
156.95
149.23
148.57
148.08
137.82
136.05
135.89
135.18
134.96
134.75
134.53
133.22
129.48
129.08
126.54
126.52
126.49
126.47
126.22
124.59
124.41
122.60
122.06
120.79
117.49
117.46
117.44
117.41
115.31
106.25
104.88
77.21
77.00
76.79
37.16
O
N
N
N
Me
CF3
N
3cq
(150 MHz, CDCl3)
f1 (ppm)

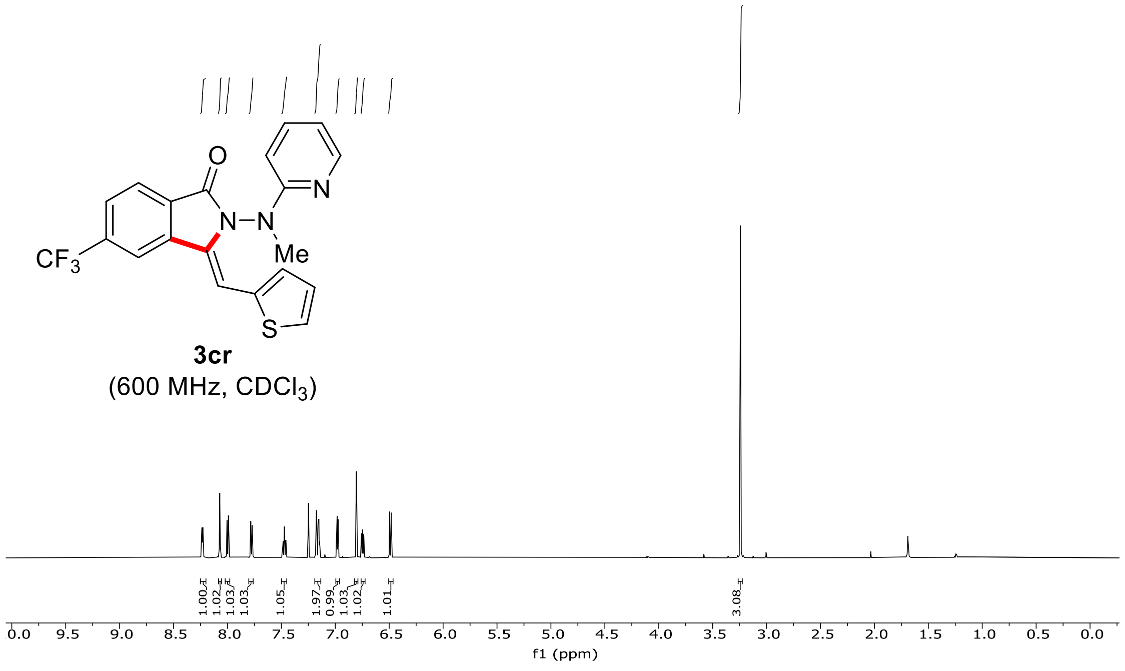
O
N
N
N
Me
CF3
S
3cr
(600 MHz, CDCl3)
1.00
1.02
1.03
1.03
1.05
1.97
0.99
1.03
1.02
1.01
3.08
f1 (ppm)

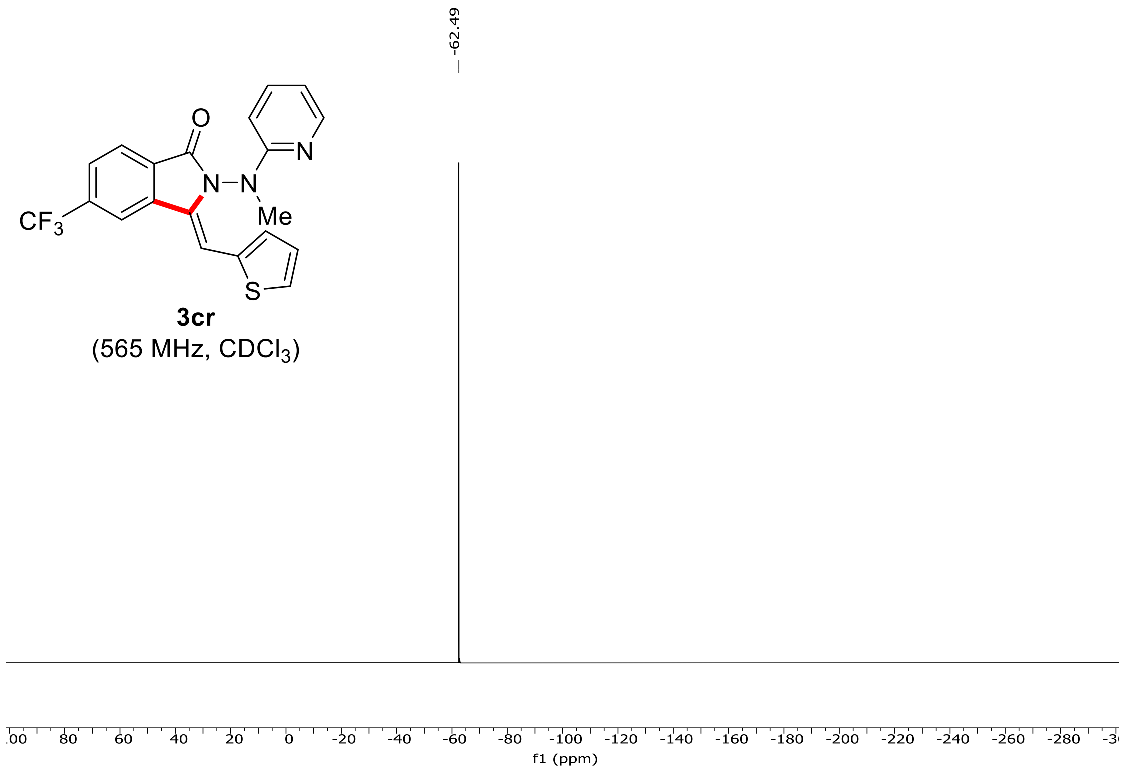

-62.49
O
N
N–N
Me
CF3
S
3cr
(565 MHz, CDCl3)
f1 (ppm)

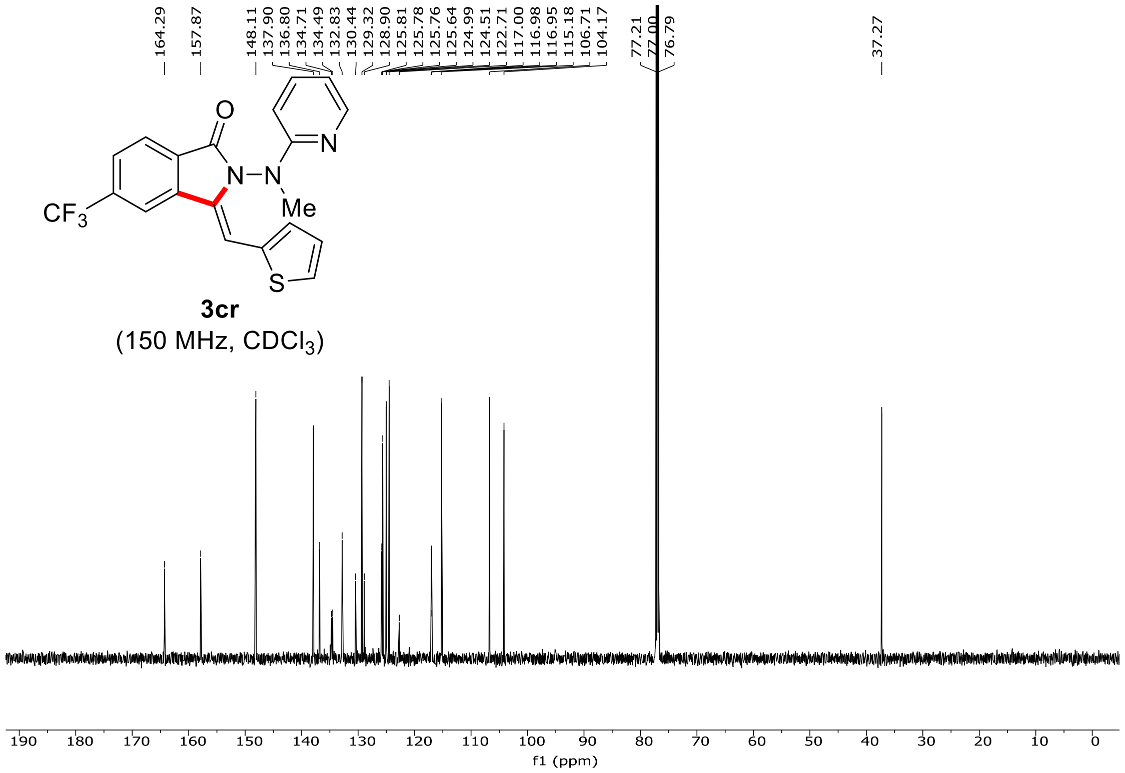

164.29
157.87
148.11
137.90
136.80
134.71
134.49
132.83
130.44
129.32
128.90
125.81
125.78
125.76
125.64
124.99
124.51
122.71
117.00
116.98
116.95
115.18
106.71
104.17
77.21
77.00
76.79
37.27
O
N
N–N
Me
CF3
S
3cr
(150 MHz, CDCl3)
f1 (ppm)

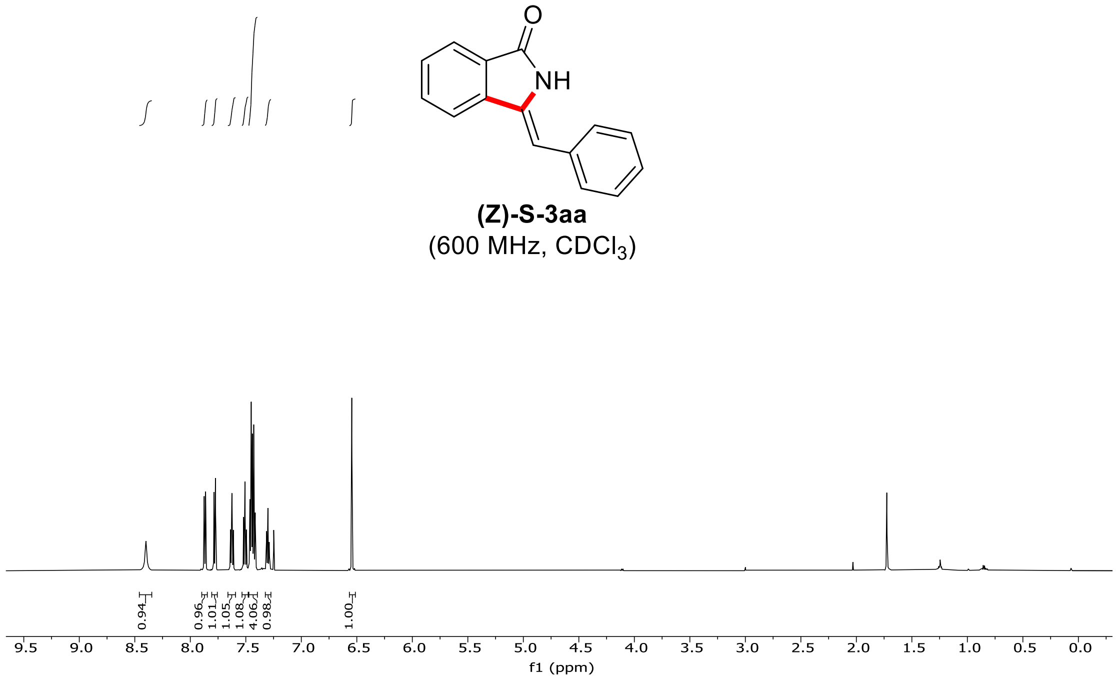
O
NH
(Z)-S-3aa
(600 MHz, $CDCl_3$)
0.94
0.96
1.01
1.05
1.08
4.06
0.98
1.00
9.5 9.0 8.5 8.0 7.5 7.0 6.5 6.0 5.5 5.0 4.5 4.0 3.5 3.0 2.5 2.0 1.5 1.0 0.5 0.0
f1 (ppm)

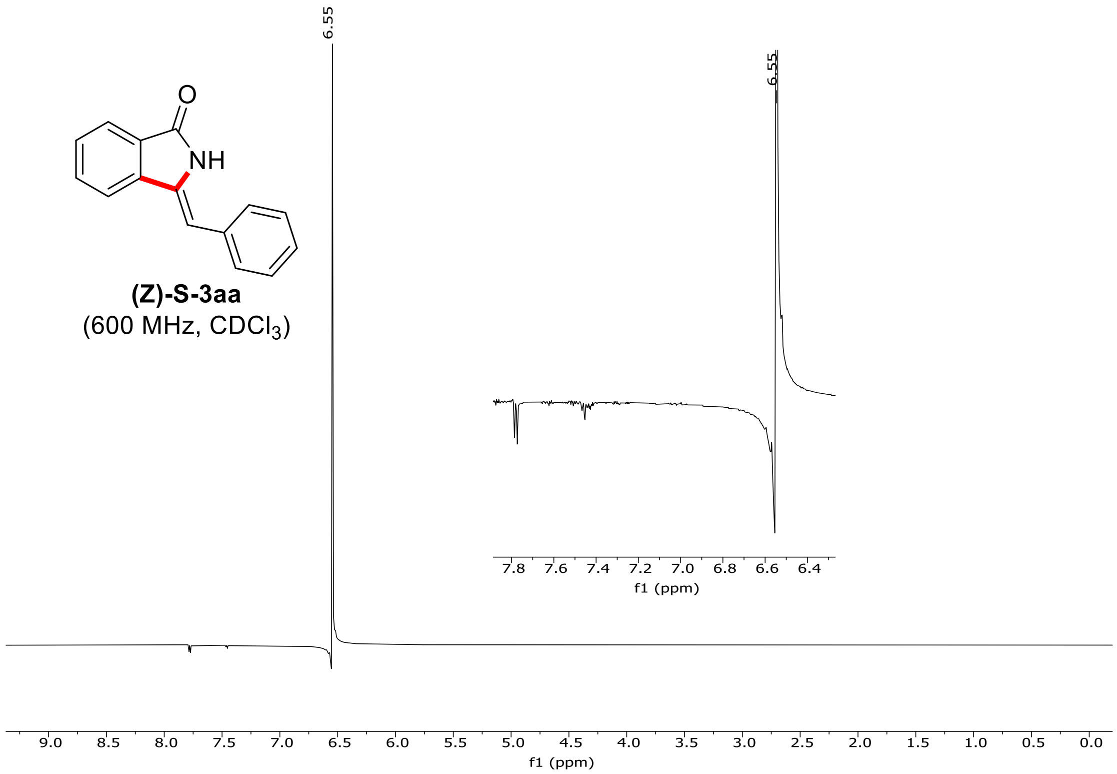
6.55
O
NH
(Z)-S-3aa
(600 MHz, $CDCl_3$)
6.55
7.8 7.6 7.4 7.2 7.0 6.8 6.6 6.4
f1 (ppm)
9.0 8.5 8.0 7.5 7.0 6.5 6.0 5.5 5.0 4.5 4.0 3.5 3.0 2.5 2.0 1.5 1.0 0.5 0.0
f1 (ppm)

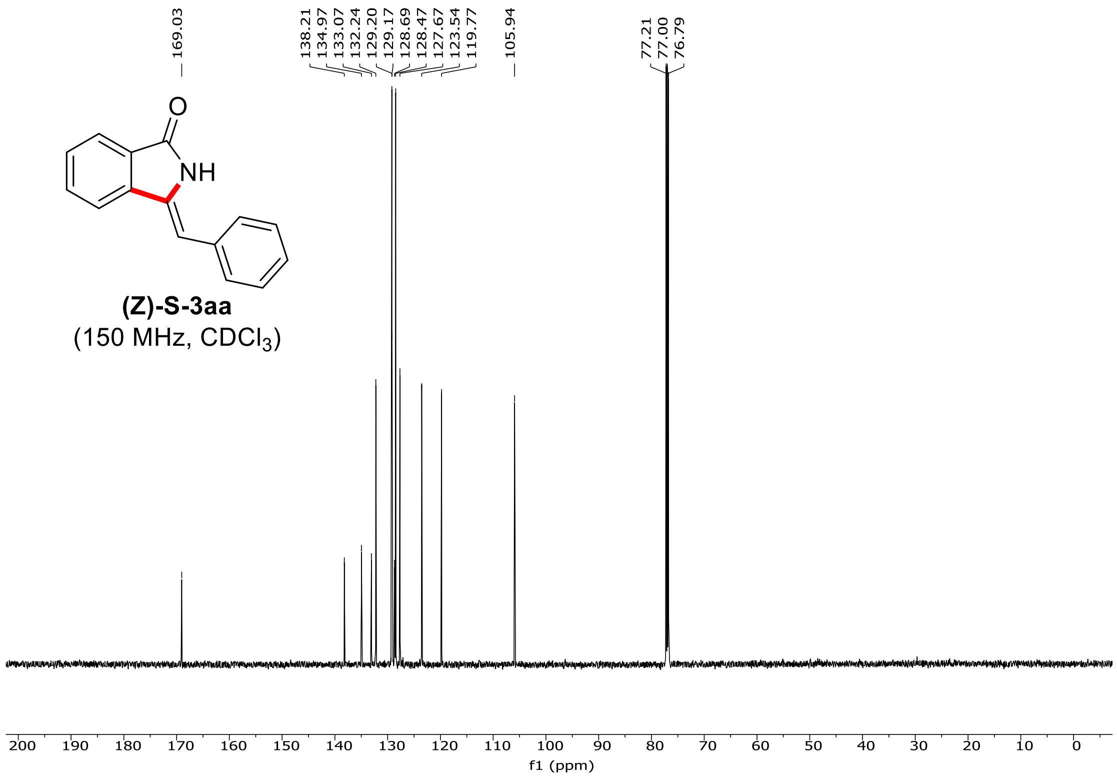

169.03
138.21
134.97
133.07
132.24
129.20
129.17
128.69
128.47
127.67
123.54
119.77
105.94
77.21
77.00
76.79
O
NH
(Z)-S-3aa
(150 MHz, CDCl3)
200 190 180 170 160 150 140 130 120 110 100 90 80 70 60 50 40 30 20 10 0
f1 (ppm)

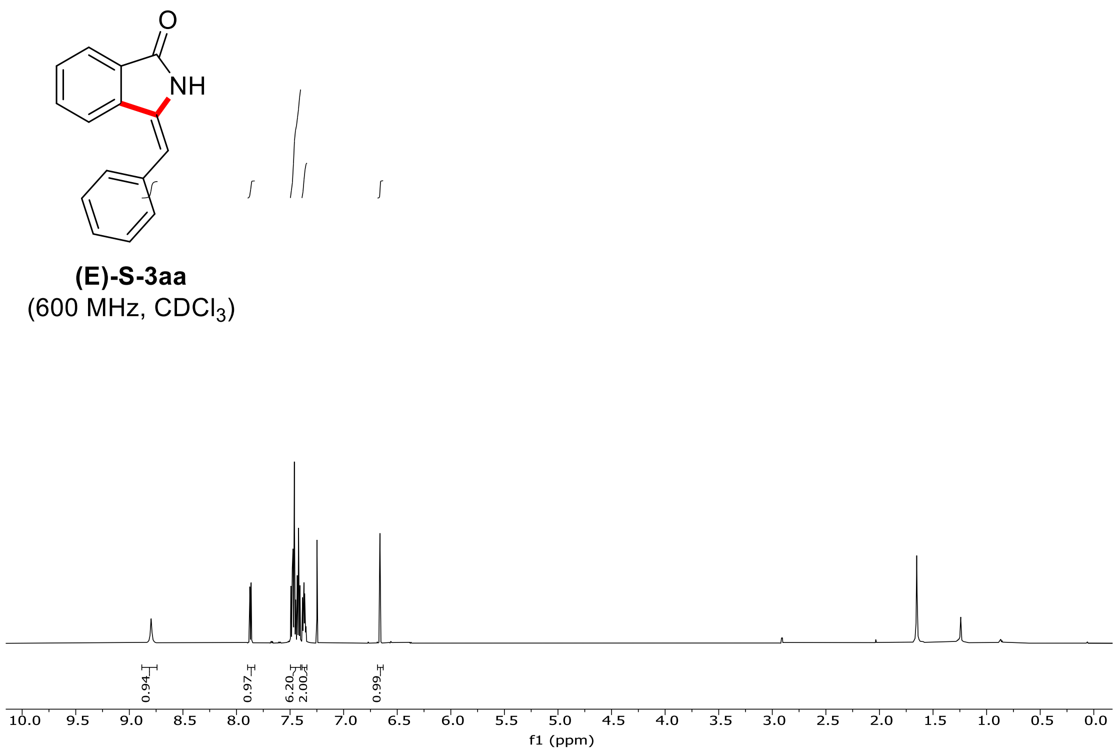

O
NH
(E)-S-3aa
(600 MHz, CDCl3)
0.94
0.97
6.20
2.00
0.99
10.0 9.5 9.0 8.5 8.0 7.5 7.0 6.5 6.0 5.5 5.0 4.5 4.0 3.5 3.0 2.5 2.0 1.5 1.0 0.5 0.0
f1 (ppm)

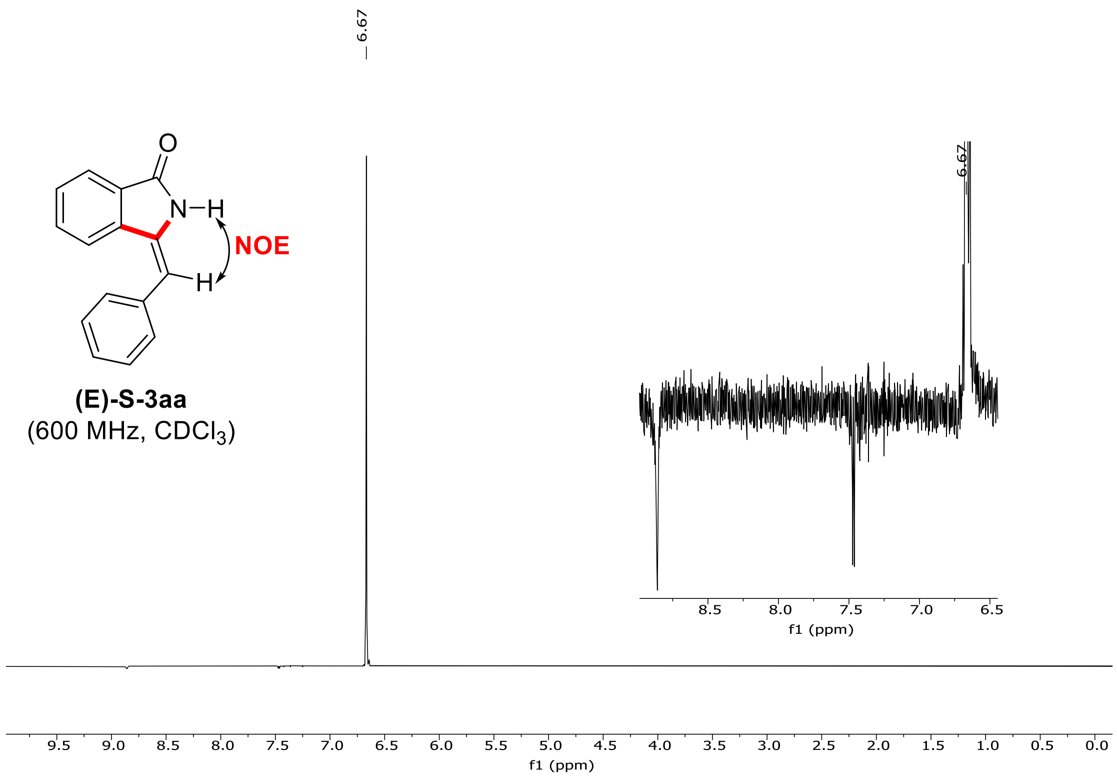

6.67
O
N−H
NOE
H
(E)-S-3aa
(600 MHz, CDCl3)
6.67
8.5
8.0
7.5
7.0
6.5
f1 (ppm)
9.5
9.0
8.5
8.0
7.5
7.0
6.5
6.0
5.5
5.0
4.5
4.0
3.5
3.0
2.5
2.0
1.5
1.0
0.5
0.0
f1 (ppm)

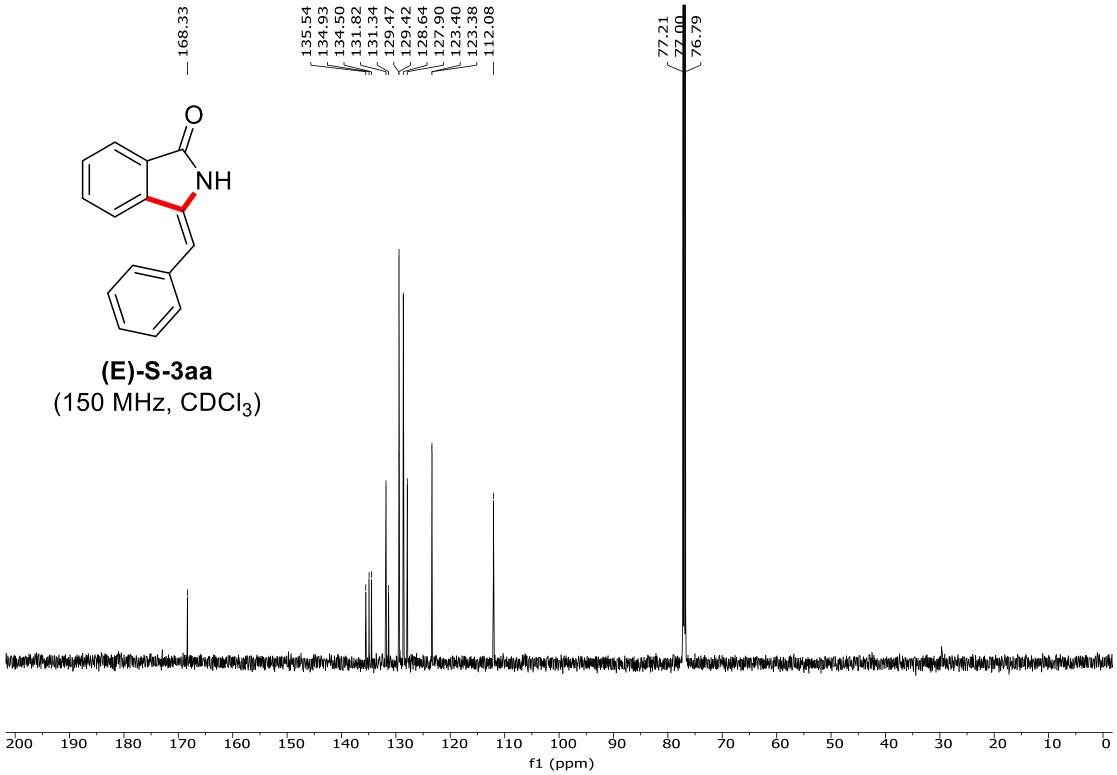

168.33
135.54
134.93
134.50
131.82
131.34
129.47
129.42
128.64
127.90
123.40
123.38
112.08
77.21
77.00
76.79
O
NH
(E)-S-3aa
(150 MHz, CDCl3)
200
190
180
170
160
150
140
130
120
110
100
90
80
70
60
50
40
30
20
10
0
f1 (ppm)
